# Clavukoellians G–K, New Nardosinane and Aristolane Sesquiterpenoids with Angiogenesis Promoting Activity from the Marine Soft Coral *Lemnalia* sp.

**DOI:** 10.3390/md18030171

**Published:** 2020-03-20

**Authors:** Qi Wang, Xuli Tang, Hui Liu, Xiangchao Luo, Ping Jyun Sung, Pinglin Li, Guoqiang Li

**Affiliations:** 1 Key Laboratory of Marine Drugs, Chinese Ministry of Education, School of Medicine and Pharmacy, Ocean University of China, Qingdao 266003, China; wangqi@hmfl.ac.cn (Q.W.);; 2 Laboratory of Marine Drugs and Biological Products, National Laboratory for Marine Science and Technology, Qingdao 266235, China; 3 College of Chemistry and Chemical Engineering, Ocean University of China, Qingdao 266100, China; tangxuli@ouc.edu.cn; 4 Institutes of Chronic Disease, Qingdao University, Qingdao 266003, China; 5 National Museum of Marine Biology and Aquarium, Pingtung 94450, Taiwan; pjsung@nmmba.gov.tw; 6 Graduate Institute of Marine Biology, National Dong Hwa University, Pingtung 94450, Taiwan

**Keywords:** *Lemnalia* sp., clavukoellian, nardosinane, aristolane, sesquiterpenoid, angiogenesis promoting activity

## Abstract

The chemical examination of the marine soft coral *Lemnalia* sp., collected at the Xisha islands in the South China Sea, resulted in the isolation of four new nardosinane-type sesquiterpenoids, namely clavukoellians G–J (**1**–**4**), and one new aristolane sesquiterpene, namely clavukoellian K (**5**), together with five known compounds, **6**–**10**. The structure elucidation of the isolated natural products was based on various spectroscopic techniques including HRESIMS and NMR, while their absolute configurations were resolved on the basis of comparisons of the ECD spectra with the calculated ECD data. The isolated new compounds **1**–**5** were evaluated for their anti- and pro- angiogenesis activities in a transgenic fluorescent zebrafish (Tg(vegfr2:GFP)) model. Quantitative analysis revealed that compound **5** displayed pro-angiogenesis activity in a PTK787-induced vascular injury zebrafish model at 2.5 μM. Data showed that compound **5** significantly promoted the angiogenesis in a dose-dependent manner.

## 1. Introduction

Nardosinane-type sesquiterpenoids have been recognized as hot-spot marine natural products with novel structures and wide biological activities in recent years [1,2]. These compounds have special structural diversity. The oxidation occurring at various sites on the 6-isopropyl dimethyl-decahydronaphthalene core of nardosinanes generates hydroxy [3], keto [4], cyclic ether [5], and lactone functionalities [6]. Most of these compounds are isolated from soft corals, such as the genus *Lemnalia*, *Paralemnalia*, *Nephthea*, and *Rhytisma* [1,2,3,4,5,6]. These coral species have interesting habitat specificity. Some of the corals, including those of *Lemnalia flava*, *Paralemnalia thyrsoides*, *Nephthea elongata*, and *Nephthea armata* were collected from the Taiwan Ocean [5,6], while *Lemnalia africana*, *Paralemnalia clavata*, and *Rhytisma fulvum* were obtained from the Indian Ocean [2]. However, our recent research on *Clavularia koellikeri* collected from Xisha islands in China has led to the isolation of six novel nardosinane compounds, clavukoellians A–F, with a highly rearranged carbon skeleton [7]. Together with the recent founding of new polyoxygenated nardosinanes (xishaflavalin A–C) [8] from Xisha *Lemnalia flava*, it is indicated that the Xisha soft corals have a remarkable novelty of this compound family. 

**Figure 1:**
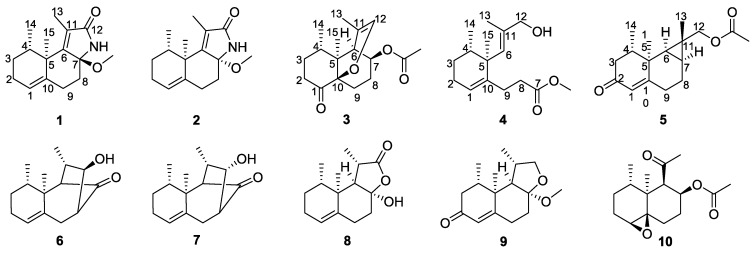
Chemical structures of compounds **1**–**10**.

In the present study, four new nardosinane sesquiterpenes clavukoellians G–J (**1**–**4**), one aristolane sesquiterpene clavukoellian K (**5**), and five known compounds (**6**–**10**) (Figure 1) were isolated from a Xisha soft coral *Lemnalia sp*. In addition, on the basis of angiogenic-related activities of clavukoellian A [7], the anti- and pro-angiogenic in zebrafish models were carried out to explore the activities of clavukoellians G–K (**1**–**5**). Clavukoellian K (**5**) displayed significant angiogenesis promoting activity in zebrafish models. Herein, details of the isolation, structure elucidation, and the biosynthetic pathway of compounds **1**–**5** are presented.

## 2. Results

**Figure 2:**
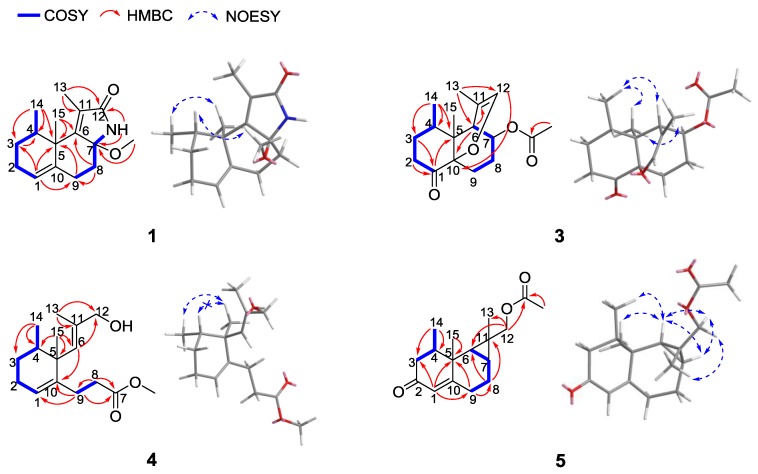
Key: COSY (bolds, blue), HMBC (arrows, red), and NOESY (dashed arrows, blue) correlations of **1**, **3**, **4**, and **5**.

**Figure 3:**
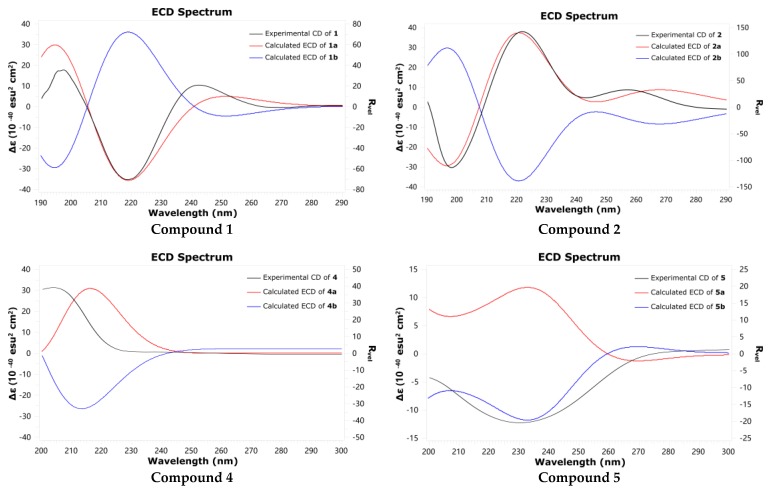
Experimental and calculated ECD spectra of compounds **1**, **2**, **4**, and **5**.

Clavukoellian G (**1**) was isolated as a white powder. Its molecular formula was determined to be C_16_H_23_NO_2_ by HRESIMS, requiring six degrees of unsaturation. The characteristic three methyl signals at *δ*_H_ 1.99 (3H, s), *δ*_H_ 1.34 (3H, s), and *δ*_H_ 1.00 (3H, d, *J* = 6.9 Hz), together with the four methylenes at *δ*_H_ 2.21, 2.04 (2H, m), *δ*_H_ 2.20, 2.04 (2H, m), *δ*_H_ 2.15 (2H, m), and *δ*_H_ 1.61, 1.54 (2H, m), disclosed the nardosinane-type sesquiterpene skeleton of clavukoellian G (**1**). The nitrogenated proton at *δ*_H_ 5.94 (1H, brs) in H NMR spectra further revealed that **1** was a member of the rare N-containing nardosinane family. Careful analysis of 1D and 2D NMR data indicated **1** was very similar to clavukoellian B, with the only difference of an additional methoxy group at *δ*_H_ 3.00 (3H, s). The methoxy group was placed at C-7 because of the obvious HMBC signal from 7-OCH_3_ to C-7. Finally, clavukoellian G (**1**) was determined to be an oxidation product of clavukoellian B and its planar structure was shown in Figure 1. To determine the relative configuration of **1**, NOESY experiments (Figure 2) were carried out to afford the related correlation signals. In the NOESY spectrum of **1**, H_3_-15 showed a correlation to H_3_-14, but no correlations were observed between H-4 and H_3_-15, revealing the co-facial orientation of H_3_-14 and H_3_-15. Furthermore, the NOESY correlation from 7-OCH_3_ to H-4, indicates that 7-OCH_3_ had an orientation different from those of H_3_-14 and H_3_-15. Thus, the relative configuration of **1** was clarified. Based on the determination of relative configuration, there are two probable forms of its final stereo configuration, **1a** (4*S*, 5*R*, 7*S*) and **1b** (4*R*, 5*S*, 7*R*), respectively. ECD calculation of these two possibilities were performed using the time-dependent density functional theory (TDDFT) at the B3LYP/APFD level. By comparison of the experimental and calculated ECD spectra of **1**, the absolute configuration of clavukoellian G (**1**) was determined to be 4*S*, 5*R*, 7*S* (**1a**), eventually (Figure 3). So, the planar and stereo structure of **1** was unambiguously assigned.

Clavukoellian H (**2**), also isolated as a white powder, gave the same molecular formula, C_16_H_23_NO_2_, as **1**, based on its HRESIMS *m/z* 262.1808 [M + H]^+^ (calcd for C_16_H_24_NO_2_, 262.1802). Detailed NMR data of **2** (Table 1 and Table 2) was almost identical to those of **1**, in addition to some slight numerical floating in chemical shifts, indicating that **1** and **2** are a pair of isomers. In order to figure out the difference between them, the NOESY experiment of **2** was conducted. The correlations of H_3_-14/H_3_-15 and 7-OCH_3_/H_3_-15 in the NOESY spectrum, indicate the co-facial orientation of H_3_-14, H_3_-15, and 7-OCH_3_ (Figure S1). Next, two model compounds, (4*S*,5*R*,7*R*)-2 (**2a**) and (4*R*,5*S*,7*S*)-2 (**2b**), were used for the ECD calculations. Finally, the assignment of the (4*S*,5*R*,7*R*) configuration for **2** (Figure 3) was confirmed due to the good agreement between the calculated overall pattern ECD curve for the **2a** stereoisomer and the experimental curve of **2**. It is worth mentioning that the chirality of C-7 in **2** was different from **1** and they are a pair of epimer isomers.

Clavukoellian I (**3**), an amorphous white powder, with a molecular formula of C_17_H_24_O_4,_ was determined by HRESIMS. From detailed analysis of the 1D and 2D NMR data (Table 1 and Table 2, Figure 1), **3** was suspected to be a 6/6/6 nardosinane tricyclic sesquiterpene with a 10,12-bridged structure, similar to clavukoellian E [7]. The main differences between **3** and clavukoellian E are in that compound **3** has the signals for two more methylene groups and one more methyl group but lacks the hydroxy proton signal. The consecutive COSY correlations of H_2_-2/H_2_-3/H-4/H_3_-14 and H-6/H-7/H_2_-8/H_2_-9, together with the HMBC correlations of H-6/C-5, C-7, C-8, and C-10; H_3_-14/C-3, C-4, and C-5; as well as H_3_-15/C-4, C-5, C-6, and C-10 (Figure 2), established the 4,4a-dimethyloctahydronaphthalen-1(2*H*)-one ring system. The HMBC correlations of H-6/C-11, C-12, and C-13; H-12/C-6, C-10, C-11, and C-13; and H_3_-13/C-6, C-11, and C-12, indicate the presence of a -CH-C(Me)=CH-O- moiety, connecting the 4,4a-dimethyloctahydronaphthalen-1(2*H*)-one ring system from C-6 to C-10. In addition, the acetoxy group was determined to be substituted at C-7 based on the related HMBC correlations. Thus, the planar structure of **3** was established. The relative configuration of **3** was determined according to the NOESY correlations from H-6 to H_3_-13, H_3_-14 and H_3_-15, from H-12 to H_3_-13, from H_3_-14 to H-6, H_3_-13 and H_3_-15, and from H_3_-15 to H-7 (Figure 2). ECD calculation was performed to assign the absolute configuration of **3** as 4*S*, 5*S*, 6*R*, 7*S,* 10*R* (Figure S2).

Clavukoellian J (**4**) was obtained as white powder and was determined to have a molecular formula of C_16_H_26_O_3_ based on the HRESIMS *m/z* 289.1782 [M + Na]^+^ (calcd for C_16_H_26_O_3_Na, 289.1774). The analysis of ^1^H NMR, ^13^C NMR, COSY, HSQC, and HMBC data (Table 1 and Table 2, Figure 2) indicated that clavukoellian J (**4**) was very similar to clavukoellian F [7], a C-6/C-7 cleavage seco-nardosinane sesquiterpenoid, with the exception that compound **4** lacks the aldehyde group but has one more methylene and one more methoxy group. Detailed 2D NMR data analysis (Figure 2), allowed the construction of the planar structure of **4**. ECD calculations of the respective **4a** (4*S*,5*S*) and **4b** (4*R*,5*R*) were performed according to the relative configuration established by the ^1^D-NOE. In the ^1^D-NOE spectrum, irradiation of H_3_-15 enhanced the signal at H_3_-14 but there was no observation of a signal at H-4, indicating that H_3_-14 and H_3_-15 are on the same side. The experimental ECD spectrum of **4** was in good agreement with the calculated ECD spectrum of **4a** (Figure 3) and it is worth mention that **4** has the opposite configuration (4*S*,5*S*) from that of clavukoellian F (4*R*,5*R*).

Clavukoellian K (**5**) was also obtained as a white powder and its molecular formula was determined to be C_17_H_24_O_3_ by HRESIMS *m/z* 277.1806 [M + H]^+^ (calcd for C_17_H_25_O_3_, 277.1798). Different from the nardosinane-type sesquiterpene of **1**–**4**, **5** was determined to be an aristolane-type sesquiterpenoid according to the 1D and 2D NMR data, which is very similar to the known compound 12-Acetoxy-1(10)-aristolene [9]. The only difference between them is the extra carbonyl moiety at C-2 in compound **5**. The relative configuration of **5** was determined by a NOESY experiment. In the NOESY spectrum of **5**, H-6 (*δ*_H_ 0.91) correlated to H-7 (*δ*_H_ 1.14), H_3_-14 (*δ*_H_ 1.08), and H_3_-15 (*δ*_H_ 1.26), H-6 and H-7 correlated to H_2_-12a, and H_3_-13 correlated to H_2_-12b—indicating that H_3_-13 had an orientation different from those of H-6, H-7, H_3_-14, and H_3_-15. The absolute configuration of compound **5** was determined by ECD calculations as 1*R*, 4*S*, 5*S*, 6*S*, 7*S*, and 11*R* (Figure 3).

**Scheme 1:**
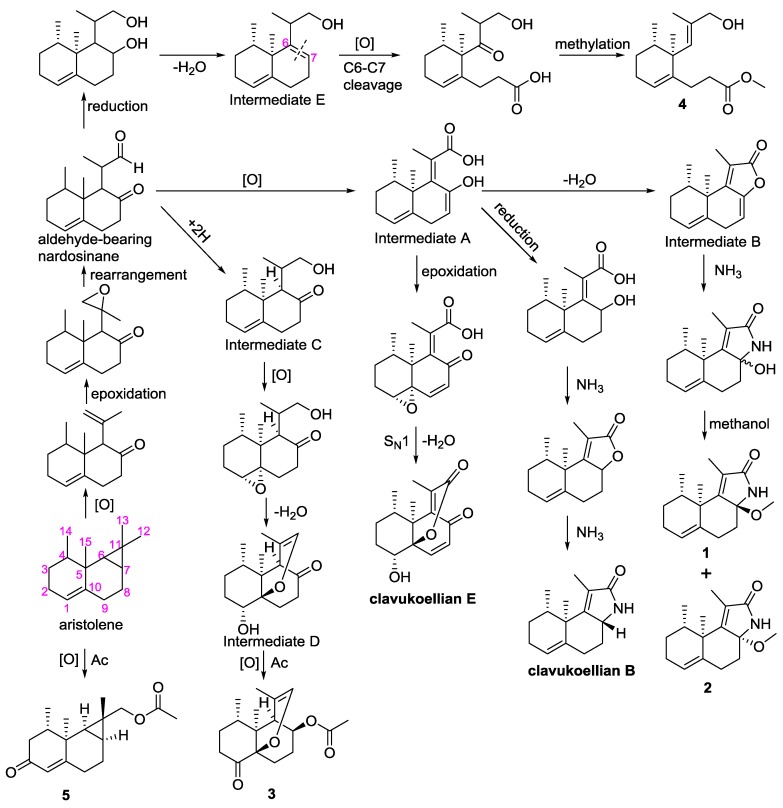
Plausible biosynthetic pathway of compounds **1**–**5** and related clavukoellians.

Starting from aristolene [2,10], the key original aldehyde-bearing nardosinane is likely to be the precursor of clavukoellians G–K (**1**–**5**). (Scheme 1). First, aldehyde-bearing nardosinane may be oxidized to intermediate A. It is suggested that compounds **1** and **2** are obtained by the methylation of the amino product of intermediate B [11], which is similar to clavukoellian B. Compound **3** is probably obtained by an oxidization of intermediate D, which is a dehydrated product by an S_N_2 attack on C-10 of the epoxidation product of intermediate C, as the clavukoellian E is probably obtained by an S_N_2 attack on C-10 of the epoxidation product of intermediate A [2]. In addition, aldehyde-bearing nardosinane can also be reduced and dehydrated to produce intermediate E, which will generate compound **4** after a C6-C7 cleavage and reduction. Compound **5** is an oxidation product of 12-acetoxy-1(10)-aristolane.

**Figure 4:**
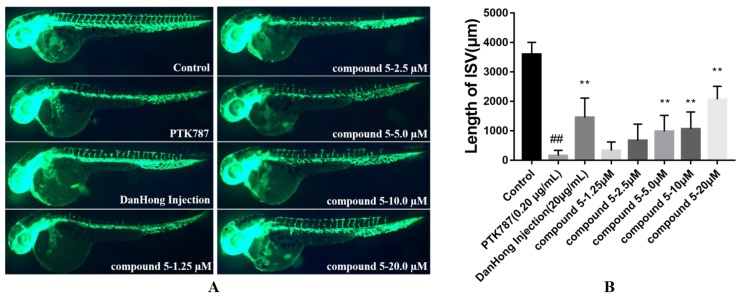
A: Images of intersomitic vessels (ISV) in transgenic fluorescent zebrafish (Tg(vegfr2:GFP)) treated with PTK787 and different concentrations (1.25, 2.5, 5, 10, and 20 μM) of **5**, using Danhong injection as a positive control. B: Quantitative analysis of the length of ISV in zebrafish treated with **5**. Data represented as mean ± SD. ^##^ *P* < 0.01 compared to the control group; ** *P* < 0.01 compared to the PTK787-induced group.

Since the angiogenesis-related activities shown in clavukoellians [7], we are committed to find more nardosinane analogues with angiogenesis activity. The anti- and pro- angiogenesis activities of compounds **1-5** were evaluated in a transgenic fluorescent zebrafish (Tg(vegfr2:GFP)) model [12,13,14,15]. Quantitative analysis revealed that compound **5** displayed pro-angiogenesis activity in a PTK787-induced vascular injury zebrafish model at 2.5 μM (Figure 4). Data showed that compound **5** significantly promoted the angiogenesis in a dose-dependent manner.

## 3. Discussion

Soft corals of the genus *Lemnalia* are a rich source of nardosinane sesquiterpenoids. However, the previous study showed a notable habitat specificity of this coral exclusively found in the Indian [2], and Taiwan [5,6] Ocean. Recently, new analogues of N-containing (clavukoellians A–D) [7] and seco- (clavukoellian F) [7] nardosinanes were isolated from *Lemnalia flava* in the South China Sea, which showed the research significance of the nardosinanes in this ocean area. The present discovery of four new nardosinane-type sesquiterpenoids, clavukoellians G–J (**1**–**4**), and one new aristolane sesquiterpene, clavukoellian K (**5**) further proves the existence of characteristic nardosinanes in the South China Sea. Clavukoellian G (**1**) and clavukoellian H (**2**) are the fifth and sixth members of the nitrogen-containing nardosinane sesquiterpenes family so far. Clavukoellian I (**3**) represents a 6/6/6 tricyclic skeleton with a ∆^11,12^ double bond which is rare in this structure family. Clavukoellian J (**4**) is a C6-C7 cleavage seco-nardosinane-type sesquiterpene related to clavukoellian E. Clavukoellian K (**5**) is a new aristolane-type sesquiterpenoid. In addition, the angiogenesis-related activities shown in clavukoellians A and K prompt researchers to explore the activity of this kind of compounds in the future.

## 4. Materials and Methods

### 4.1. General Experimental Procedures

Optical rotations were measured on a JASCO P-1020 digital polarimeter. UV spectra were recorded on a Beckman DU640 spectrophotometer. ECD spectra were obtained on an Applied Photophysics Chirascan spectropolarimeter. IR spectra were taken on a Nicolet NEXUS 470 spectrophotometer in KBr disks. NMR spectra were measured on a Bruker AVANCE 500 spectrometer. The 7.26 ppm and 77.2 ppm resonances of CDCl_3_ were used as internal references for ^1^H and ^13^C NMR spectra, respectively. HRESIMS data were measured on Micromass Q-Tof Ultima Global GAA076LC and Thermo Scientific LTQ orbitrap XL mass spectrometers. Semi-preparative HPLC utilized an ODS column (YYMC-Pack ODS-A, 10 × 250 mm, 5 µm, 1.5 mL/min). Silica gel (200-300 mesh, Qingdao) was used for column chromatography, and precoated silica gel plates (GF254, Qingdao) were used for TLC, and spots visualized by heating SiO_2_ plates sprayed with 5% H_2_SO_4_ in EtOH.

### 4.2. Soft Coral Material

The marine soft coral *Lemnalia* sp. was collected from Xisha Island in the South China Sea in December 2014, and was frozen immediately after collection. The specimen was identified by Nicole J. de Voogd, National Museum of Natural History, Leiden, The Netherlands. The voucher specimen (No. XS-YG-12) was deposited at the State Key Laboratory of Marine Drugs, Ocean University of China, P. R. China.

### 4.3. Extraction and Isolation

*Lemnalia* sp. (1.2 kg, wet weight) was crushed and then extracted with MeOH four times (three days each time) at room temperature. The combined solutions were concentrated in vacuo and the residue was subsequently desalted to yield the organic extract (40.7 g). The extract was subjected to silica gel vacuum liquid chromatography (VLC), eluting with a gradient of petroleum ether/EtOAc (from 50:0 to 1:1, v:v) and subsequently CH_2_Cl_2_/MeOH (from 10:1 to 0:1, v:v) to obtain 12 fractions (Fr.1-Fr.12). Fr.11 (5.2 g) was subjected to a silica gel CC (petroleum ether/acetone, from 20:1 to 1:1, v:v) to give five fractions Fr.11-1–Fr.11-5. Fr.11-4 (500 mg) was then purified by semi-preparative HPLC (ODS, 5 µm, 250 × 10 mm; MeOH/H_2_O, 70:30, v/v; 1.5 mL/min) to afford compound **1** (*t*_R_ = 31.0 min, 5.0 mg) and compound **2** (*t*_R_ = 43.0 min, 3.0 mg). Fr.10 (6.5 g) was subjected to a silica gel CC (petroleum ether/acetone, from 50:1 to 1:1, v:v) to give seven fractions Fr.10-1–Fr.10-7. Fr.10-2 (2.3 g) was subjected to a silica gel CC (petroleum ether/acetone, from 50:1 to 1:1, v:v) to give nine fractions Fr.10-2-1–Fr.10-2-9. Fr.10-2-3 (560.0 mg) was then subjected to a silica gel CC (petroleum ether/EtOAc, from 30:1 to 1:1, v:v) to give four fractions Fr.10-2-3-1–Fr.10-2-3-4. Fr.10-2-3-3 was then purified by semi-preparative HPLC (ODS, 5 µm, 250 × 10 mm; MeOH/H_2_O, 80:20, v/v; 1.5 mL/min) to afford compound **5** (*t*_R_ = 11.3 min, 4.0 mg). Fr.10-2-4 (400 mg) was then subjected to a silica gel CC (petroleum ether/EtOAc, from 30:1 to 1:1, v:v) to give seven fractions Fr.10-2-4-1–Fr.10-2-4-7. Fr.10-2-4-6 was then purified by semi-preparative HPLC (ODS, 5 µm, 250 × 10 mm; MeOH/H_2_O, 80:20, v/v; 1.5 mL/min) to afford compound **3** (*t*_R_ = 21.5 min, 5.0 mg). Fr.10-2-6 (500 mg) was subjected to a silica gel CC (petroleum ether/acetone, from 20:1 to 1:1, v:v) to give five fractions Fr.10-2-6-1–Fr.10-2-6-5. Fr.10-2-6-3 (210 mg) was then purified by semi-preparative HPLC (ODS, 5 µm, 250 × 10 mm; CH_3_CN/H_2_O, 75:25, v/v; 1.5 mL/min) to afford compound **4** (*t*_R_ = 29.0 min, 3.0 mg).

*Clavukoellian G* (**1**): white, amorphous powder; $\left[\alpha \right]{\;}_{D}^{20}$ −27 (*c* 0.2, MeOH); UV (MeOH) λ_max_ 200 nm; ^1^H and ^13^C NMR data, Table 1 and Table 2; HRESIMS *m/z* 262.1808 [M + H]^+^ (calcd for C_16_H_24_NO_2_, 262.1802).

*Clavukoellian H* (**2**): white, amorphous powder; $\left[\alpha \right]{\;}_{D}^{20}$ −29 (*c* 0.2, MeOH); UV (MeOH) λ_max_ 200 nm; ^1^H and ^13^C NMR data, Table 1 and Table 2; HRESIMS *m/z* 262.1808 [M + H]^+^ (calcd for C_16_H_24_NO_2_, 262.1802).

*Clavukoellian I* (**3**): white, amorphous powder; $\left[\alpha \right]{\;}_{D}^{20}$ −21 (*c* 0.2, MeOH); UV (MeOH) λ_max_ 200 nm; ^1^H and ^13^C NMR data, Table 1 and Table 2; HRESIMS *m/z* 293.1745 [M + H]^+^ (calcd for C_17_H_25_O_4_, 273.1747).

*Clavukoellian J* (**4**): white, amorphous powder; $\;\left[\alpha \right]{\;}_{D}^{20}$ −27 (*c* 0.2, MeOH); UV (MeOH) λ_max_ 200 nm; ^1^H and ^13^C NMR data, Table 1 and Table 2; HRESIMS *m/z* 289.1782 [M + Na]^+^ (calcd for C_16_H_26_O_3_Na, 289.1774).

*Clavukoellian K* (**5**): white, amorphous powder; $\;\left[\alpha \right]{\;}_{D}^{20}$ −17 (*c* 0.2, MeOH); UV (MeOH) λ_max_ 200 nm; ^1^H and ^13^C NMR data, Table 1 and Table 2; HRESIMS *m/z* 277.1806 [M + H]^+^ (calcd for C_17_H_25_O_3_, 277.1798).

### 4.4. Computational Section

The ECD computational calculation was carried out as previously described [7].

### 4.5. Promoting Angiogenesis Assay

#### 4.5.1. Zebrafish Maintenance

Adult zebrafish were cultivated by Qilu University of Technology (Jinan, China). Transgenic zebrafish (Tg(vegfr2:GFP)) expressing enhanced green fluorescent protein (EGFP) in intersomitic vessels (ISV) were used in this study. The conditions of the maintenance were complied with guidelines of the Organization for Economic Co-operation and Development (OECD). The zebrafish were maintained under a 14/10 h light/dark cycle at the temperature (28 ± 0.5 °C) in a closed flow-through system with charcoal-filtered tap water to ensure normal spawning [12,13,14,15].

#### 4.5.2. PTK787-Induced Vessel Loss Model of Zebrafish

A model of vascular insufficiency in zebrafish induced by PTK787 was used to evaluate the effect of compound **5** on angiogenesis. Healthy zebrafish larvae were selected into 24-well plates (*n* = 10/well) in a 2 mL final volume of embryo medium at 24 h post fertilization and divided into six groups: a control group (fresh fish water), a model group: 0.2 μg/mL PTK787 (Abcam (Shanghai) Trading Co., Ltd., China), a positive drug group: 20 μg/mL Danhong injection (Shandong Danhong Pharmaceutical Co., Ltd., China) and five compound **5** groups (1.25, 2.5, 5, 10, and 20 μM). VEGFR tyrosine kinase inhibitor PTK787 was added to the drug groups and incubated for 3 h before treatment with different concentrations of compound **5** for 24 h. All treatments were performed in triplicate. Each zebrafish larva was photographed by a fluorescence microscope (AXIO, Zom.V16), and the length of intersomitic vessels (ISV) was calculated through Image-Pro Plus software. One-way analysis of variance was calculated by GraphPad Prism 7.00 software [12,13,14,15].

## 5. Conclusions

In conclusion, four new nardosinane-type sesquiterpenes, clavukoellians G–J (**1**–**4**), one new aristolane sesquiterpene, clavukoellian K (**5**), together with five known compounds, **6**–**10**, were isolated from the soft coral *Lemnalia* sp. Among them, clavukoellian G (**1**) and clavukoellian H (**2**) are a pair of epimer isomers, which are the analogues of the reported nitrogen-containing nardosinane sesquiterpenes clavukoellians A–D, and there are only six members existing in the *N*-containing nardosinane family so far. Clavukoellian I (**3**) is a 6/6/6 tricyclic sesquiterpene with a ∆^11,12^ double bond connecting C6–C10. Clavukoellian J (**4**) is a rare C6–C7 cleavage seco-nardosinane-type sesquiterpene related to clavukoellian E. Clavukoellian K (**5**) is a new member of the aristolane-type sesquiterpenoid family. These compounds include three new skeletons (*N*-containing, seco- and 6,10-bridged) which are closely related to clavukoellians A–F. The discovery of them enriched the structural diversity of the nardosinane sesquiterpene family. Compound **5** showed significant pro-angiogenic activity in the zebrafish model and could provide a model compound to explore angiogenesis-related activities.

## Figures and Tables

**Table 1:** ^1^H NMR (500 MHz) data of compounds **1**–**5** in CDCl_3_ (*δ* in ppm*, J* in Hz).

No.	1	2	3	4	5
NH	5.94, brs	5.91, brs			
1	5.40, s	5.47, d (4.5)		5.38, m	5.78, s
2	2.20, m	1.96, m	2.32, d (5.6)	2.06, m	
	2.04, m				
3	1.61, m	1.96, m	2.19, m	1.44, m	2.28, m
	1.54, m	1.46, m	2.24, m		
4	1.93, m	2.36, m	2.46, m	1.86, m	2.28, m
5					
6			2.39, d (3.5)	5.37, s	0.91, d (9.6)
7			5.41, m		1.14, dd (9.8, 3.2)
8	2.26, m 2.04, m	2.28, d (12.7) 1.49, m	1.91, m	2.43, m	2.22, m 1.58, m
9	2.15, m	2.73, t (14.2) 2.04, m	1.72, m	2.22, t (7.8)	2.47, m 2.09, m
10					
11					
				
12			6.24, s	3.96, s	3.88, d (10.7) 3.63, d (11.0)
13	1.99, s	2.04, s	1.62, s	1.66, s	1.05, s
14	1.00, d (6.9)	1.07, d (6.5)	0.85, d (6.8)	0.86, d (6.8)	1.08, d (4.5)
15	1.34, s	1.31, s	1.25, s	1.05, s	1.26, d (0.6)
Ac			2.04, s		2.06, d (0.6)
OMe-7	3.00, s	3.07, s		3.66, s	

**Table 2:** ^13^C NMR (125 MHz) data of compounds **1**–**5** in CDCl_3_ (*δ* in ppm).

Carbon	1	2	3	4	5
1	120.2, CH	122.8, CH	208.0, C	120.9, CH	125.6, CH
2	25.0, CH_2_	28.9, CH_2_	49.1, CH_2_	25.8, CH_2_	198.9, C
3	26.1, CH_2_	27.0, CH_2_	45.3, CH_2_	26.9, CH_2_	42.6, CH_2_
4	35.6, CH	34.6, CH	33.4, CH	38.3, CH	36.5, CH
5	43.5, C	45.9, C	37.2, C	43.2, C	38.3, C
6	157.1, C	155.8, C	43.9, CH	134.7, CH	31.0, CH
7	90.5, C	90.7, C	72.9, CH	174.5, C	16.7, CH
8	37.4, CH_2_	40.2, CH_2_	23.8, CH_2_	33.6, CH_2_	19.4, CH_2_
9	28.0, CH_2_	25.6, CH_2_	34.7, CH_2_	27.1, CH_2_	30.4, CH_2_
10	142.5, C	142.1, C	80.2, C	143.0, C	172.8, C
11	131.5, C	127.2, C	104.9, C	135.7, C	23.2, C
12	173.1, C	172.4, C	139.3, CH	70.3, CH_2_	74.7, CH_2_
13	11.0, CH_3_	11.0, CH_3_	19.1, CH_3_	14.2, CH_3_	13.3, CH_3_
14	17.5, CH_3_	20.3, CH_3_	14.9, CH_3_	17.4, CH_3_	15.2, CH_3_
15	19.0, CH_3_	16.8, CH_3_	14.7, CH_3_	22.1, CH_3_	21.7, CH_3_
Ac			170.7, C		171.4, C
			21.5, CH_3_		21.2, CH_3_
OMe-7	50.3, CH_3_	50.3, CH_3_		51.7, CH_3_	

## Supplementary Materials

The following are available online at https://www.mdpi.com/1660-3397/18/3/171/s1: 1D and 2D NMR, HERSIMS data for compounds **1**−**5**, and ECD spectra of compounds **3**.
