# Supplementary material for: Clavukoellians G–K, New Nardosinane and Aristolane Sesquiterpenoids with Angiogenesis Promoting Activity from the Marine Soft Coral Lemnalia sp
Source: Mar Drugs. 2020 Mar 20;18(3):171. doi: 10.3390/md18030171 (PMC7143192; doi:10.3390/md18030171)
Supplement: Supplementary file 1 [file marinedrugs-18-00171-s001.pdf]

Supplementary Material

Clavukoellians G-K, New Nardosinane and Aristolane Sesquiterpenoids with Angiogenesis Promoting Activity from the Marine Soft coral *Lemnalia* sp.

Qi Wang [1,2,4,†], Xuli Tang [3,†], Hui Liu [1,2], Xiangchao Luo [1,2], Ping Jyun Sung [5,6], Pinglin Li [1,2,*], and Guoqiang Li [1,2,*]

[1] Key Laboratory of Marine Drugs, Chinese Ministry of Education, School of Medicine and Pharmacy, Ocean University of China, Qingdao 266003, China; wangqi@hmfl.ac.cn (Q.W.); 1498740241@qq.com (H.L.); 517708133@qq.com (X.L.)

[2] Laboratory of Marine Drugs and Biological Products, National Laboratory for Marine Science and Technology, Qingdao 266235, China; wangqi@hmfl.ac.cn (Q.W.); 1498740241@qq.com (H.L.); 517708133@qq.com (X.L.)

[3] College of Chemistry and Chemical Engineering, Ocean University of China, Qingdao 266100, China; tangxuli@ouc.edu.cn (X.T.)

[4] Institutes of Chronic Disease, Qingdao University, Qingdao 266003, China; wangqi@hmfl.ac.cn (Q.W.)

[5] National Museum of Marine Biology and Aquarium, Pingtung 94450, Taiwan; pjsung@nmmba.gov.tw (P.J.S.)

[6] Graduate Institute of Marine Biology, National Dong Hwa University, Pingtung 94450, Taiwan; pjsung@nmmba.gov.tw (P.J.S.)

* Correspondence: lipinglin@ouc.edu.cn (P.L.); liguoqiang@ouc.edu.cn (G.L.); Tel.: +86-532-82033054 (P.L.); +86-532-82032323 (G.L.)

† The authors contributed equally to this work.

Table of Contents

Figure S1	Key COSY (bolds, blue), HMBC (arrows, red), and NOESY (dashed arrows, blue) correlations of **2**.	4
Figure S2	Experimental and calculated ECD spectra of **3**.	4
Figure S3	1H NMR spectrum of calvukoellian G (**1**) in $CDCl_3$ (500 MHz).	5
Figure S4	Enlarged ^1H NMR spectrum of calvukoellian G (**1**) in $CDCl_3$ (500 MHz).	6
Figure S5	^{13}C NMR spectrum of calvukoellian G (**1**) in $CDCl_3$ (125 MHz).	7
Figure S6	^{13}C NMR and DEPT spectrum of calvukoellian G (**1**) in $CDCl_3$ (125 MHz).	8
Figure S7	^1H-^1H COSY spectrum of calvukoellian G (**1**) in $CDCl_3$ (500 MHz).	9
Figure S8	HSQC spectrum of calvukoellian G (**1**) in $CDCl_3$ (500 MHz).	10
Figure S9	HMBC spectrum of calvukoellian G (**1**) in $CDCl_3$ (500 MHz).	11
Figure S10	NOESY spectrum of calvukoellian G (**1**) in $CDCl_3$ (500 MHz).	12
Figure S11	NOESY spectrum of calvukoellian G (**1**) in $CDCl_3$ (500 MHz).	13
Figure S12	HRESIMS data of calvukoellian G (**1**).	14
Figure S13	^1H NMR spectrum of calvukoellian H (**2**) in $CDCl_3$ (500 MHz).	15
Figure S14	Enlarged ^1H NMR spectrum of calvukoellian H (**2**) in $CDCl_3$ (500 MHz).	16
Figure S15	^{13}C NMR spectrum of calvukoellian H (**2**) in $CDCl_3$ (125 MHz).	17
Figure S16	^{13}C NMR and DEPT spectrum of calvukoellian H (**2**) in $CDCl_3$ (125 MHz).	18
Figure S17	^1H-^1H COSY spectrum of calvukoellian H (**2**) in $CDCl_3$ (500 MHz).	19
Figure S18	HSQC spectrum of calvukoellian H (**2**) in $CDCl_3$ (500 MHz).	20
Figure S19	HMBC spectrum of calvukoellian H (**2**) in $CDCl_3$ (500 MHz).	21
Figure S20	NOESY spectrum of calvukoellian H (**2**) in $CDCl_3$ (500 MHz).	22
Figure S21	NOESY spectrum of calvukoellian H (**2**) in $CDCl_3$ (500 MHz).	23
Figure S22	HRESIMS data of calvukoellian H (**2**).	24
Figure S23	^1H NMR spectrum of calvukoellian I (**3**) in $CDCl_3$ (500 MHz).	25
Figure S24	Enlarged ^1H NMR spectrum of calvukoellian I (**3**) in $CDCl_3$ (500 MHz).	26
Figure S25	^{13}C NMR spectrum of calvukoellian I (**3**) in $CDCl_3$ (125 MHz).	27
Figure S26	^{13}C NMR and DEPT spectrum of calvukoellian I (**3**) in $CDCl_3$ (125 MHz).	28
Figure S27	^1H-^1H COSY spectrum of calvukoellian I (**3**) in $CDCl_3$ (500 MHz).	29
Figure S28	HSQC spectrum of calvukoellian I (**3**) in $CDCl_3$ (500 MHz).	30
Figure S29	HMBC spectrum of calvukoellian I (**3**) in $CDCl_3$ (500 MHz).	31
Figure S30	NOESY spectrum of calvukoellian I (**3**) in $CDCl_3$ (500 MHz).	32
Figure S31	NOESY spectrum of calvukoellian I (**3**) in $CDCl_3$ (500 MHz).	33
Figure S32	NOESY spectrum of calvukoellian I (**3**) in $CDCl_3$ (500 MHz).	34
Figure S33	HRESIMS data of calvukoellian I (**3**).	35

Figure S34	^{1}H NMR spectrum of calvukoellian J (**4**) in $CDCl_3$ (500 MHz).	36
Figure S35	Enlarged ^{1}H NMR spectrum of calvukoellian J (**4**) in $CDCl_3$ (500 MHz).	37
Figure S36	^{13}C NMR spectrum of calvukoellian J (**4**) in $CDCl_3$ (500 MHz).	38
Figure S37	^{13}C NMR and DEPT spectrum of calvukoellian J (**4**) in $CDCl_3$ (500 MHz).	39
Figure S38	^{1}H-^{1}H COSY spectrum of calvukoellian J (**4**) in $CDCl_3$ (500 MHz).	40
Figure S39	HSQC spectrum of calvukoellian J (**4**) in $CDCl_3$ (500 MHz).	41
Figure S40	HMBC spectrum of calvukoellian J (**4**) in $CDCl_3$ (500 MHz).	42
Figure S41	1D-NOE spectrum of calvukoellian J (**4**) in $CDCl_3$ (500 MHz).	43
Figure S42	1D-NOE spectrum of calvukoellian J (**4**) in $CDCl_3$ (500 MHz).	44
Figure S43	HRESIMS data of calvukoellian J (**4**).	45
Figure S44	^{1}H NMR spectrum of calvukoellian K (**5**) in $CDCl_3$ (500 MHz).	46
Figure S45	Enlarged ^{1}H NMR spectrum of calvukoellian K (**5**) in $CDCl_3$ (500 MHz).	47
Figure S46	^{13}C NMR spectrum of calvukoellian K (**5**) in $CDCl_3$ (125 MHz).	48
Figure S47	^{13}C NMR and DEPT spectrum of calvukoellian K (**5**) in $CDCl_3$ (125 MHz).	49
Figure S48	^{1}H-^{1}H COSY spectrum of calvukoellian K (**5**) in $CDCl_3$ (500 MHz).	50
Figure S49	HSQC spectrum of calvukoellian K (**5**) in $CDCl_3$ (500 MHz).	51
Figure S50	HMBC spectrum of calvukoellian K (**5**) in $CDCl_3$ (500 MHz).	52
Figure S51	NOESY spectrum of calvukoellian K (**5**) in $CDCl_3$ (500 MHz).	53
Figure S52	NOESY spectrum of calvukoellian K (**5**) in $CDCl_3$ (500 MHz).	54
Figure S53	HRESIMS data of calvukoellian K (**5**).	55

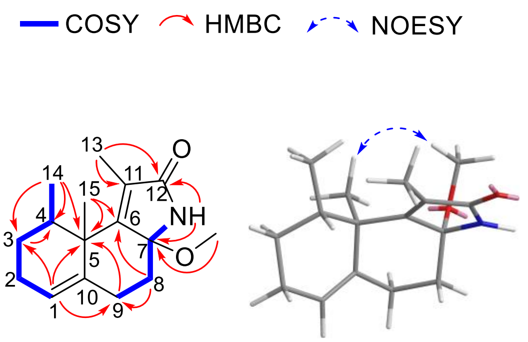

2

Figure S1. Key COSY (bolds, blue), HMBC (arrows, red), and NOESY (dashed arrows, blue) correlations of **2**.

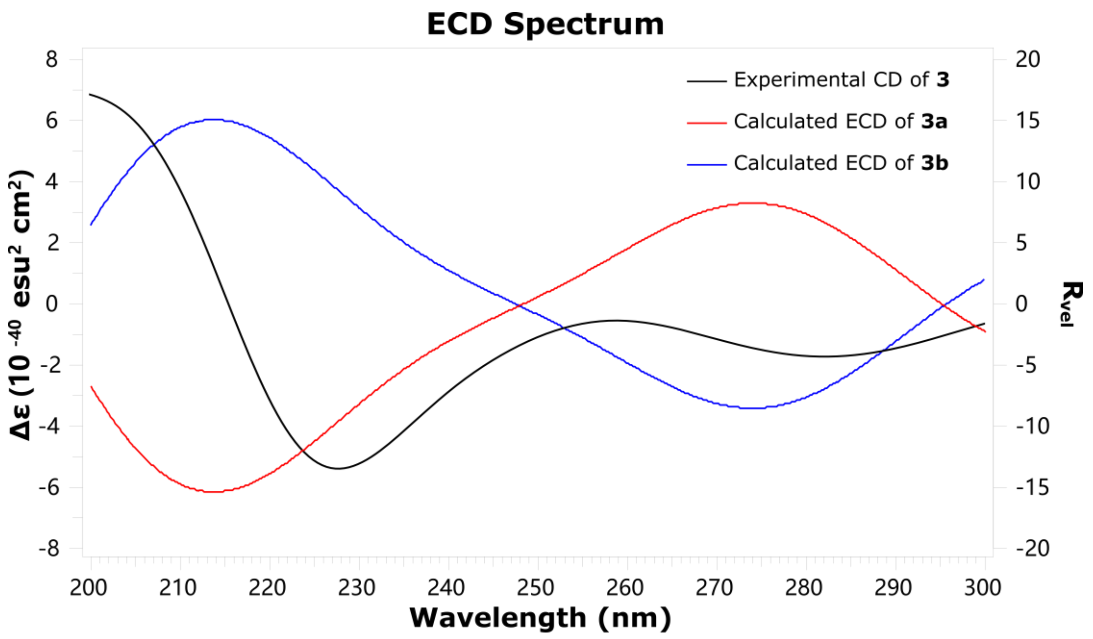

Figure S2. Experimental and calculated ECD spectra of **3**.

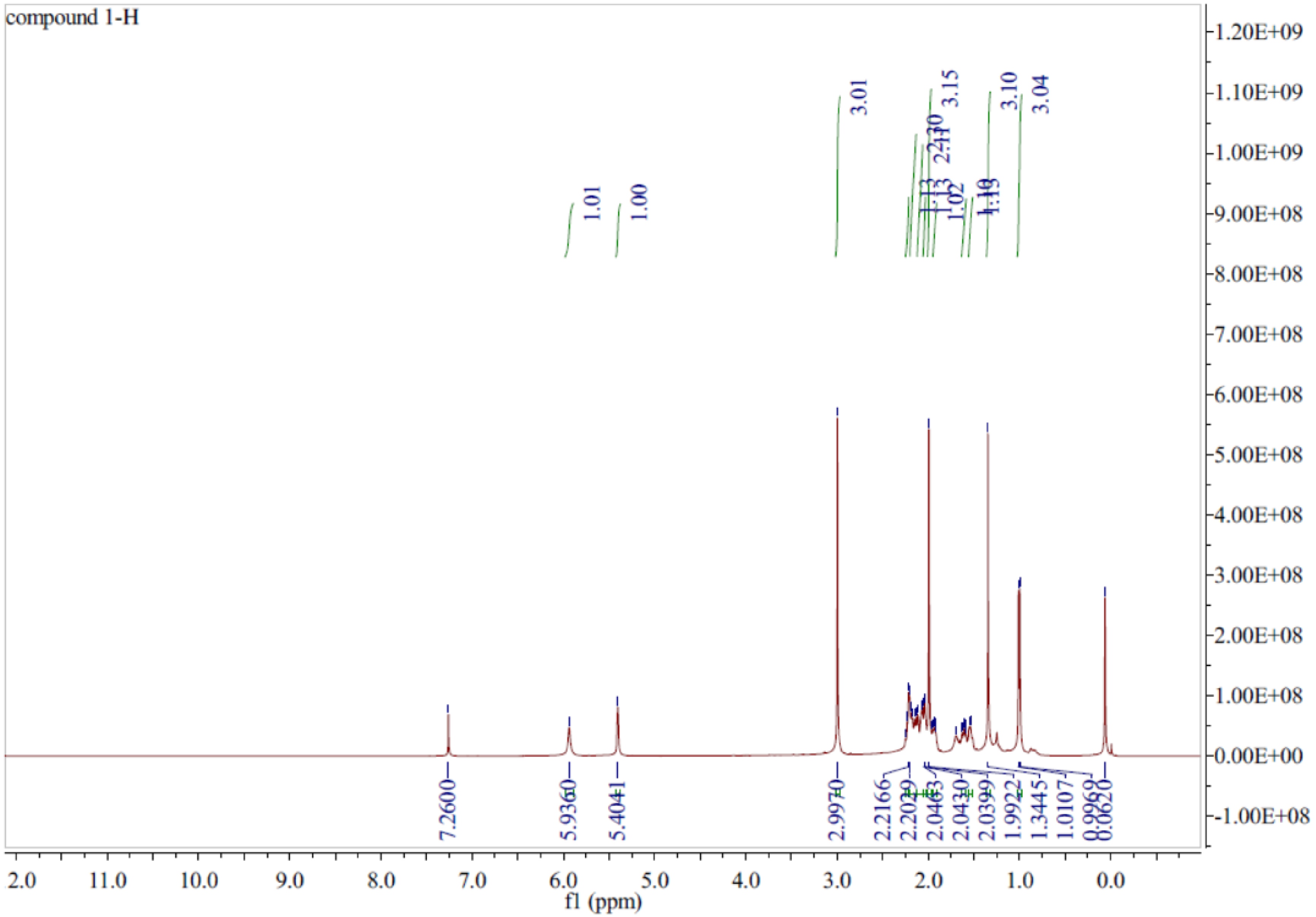

Figure S3. ^{1}H NMR spectrum of calvukoellian G (**1**) in $CDCl_3$ (500 MHz).

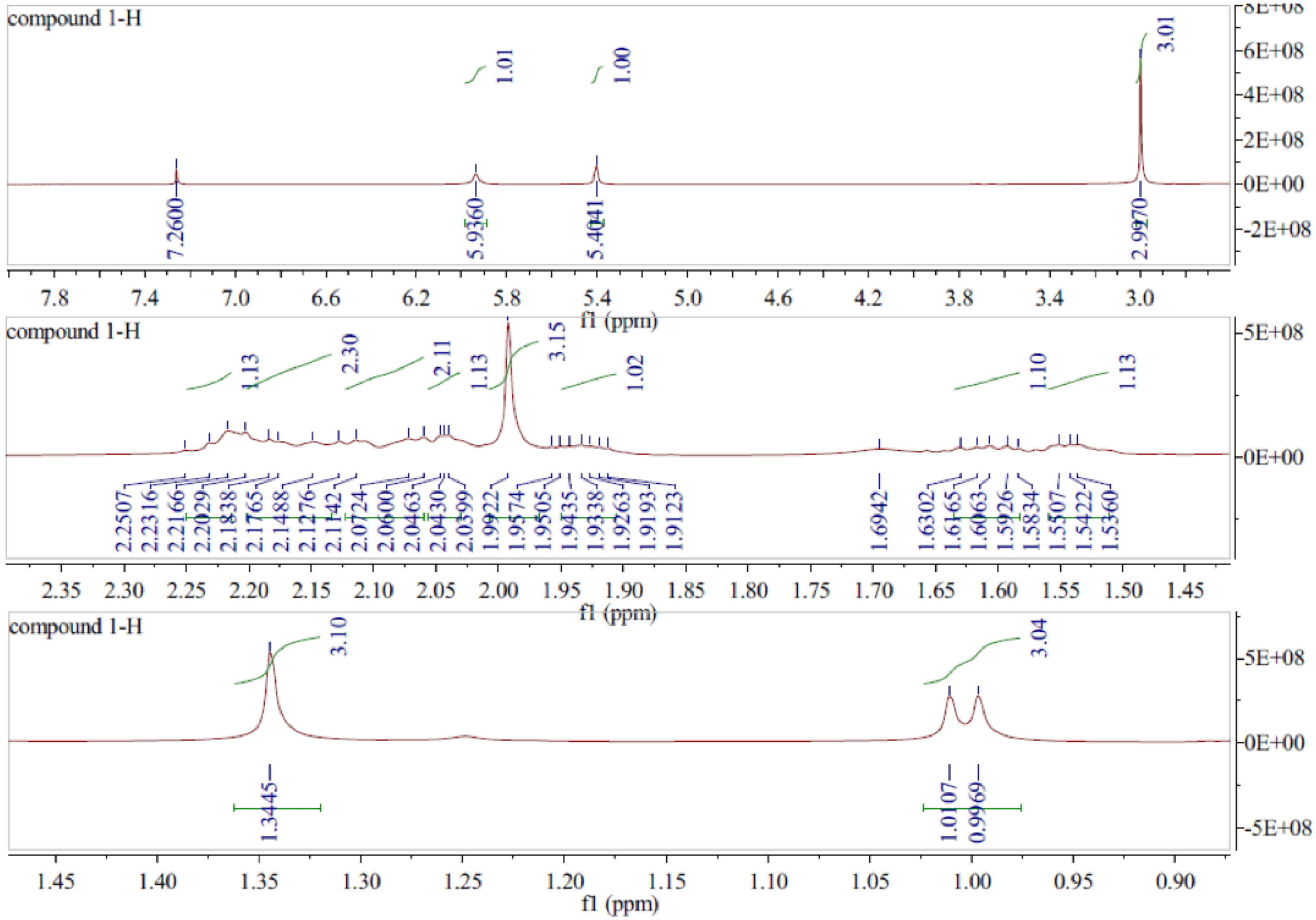

Figure S4. Enlarged ^{1}H NMR spectrum of calvukoellian G (**1**) in $CDCl_3$ (500 MHz).

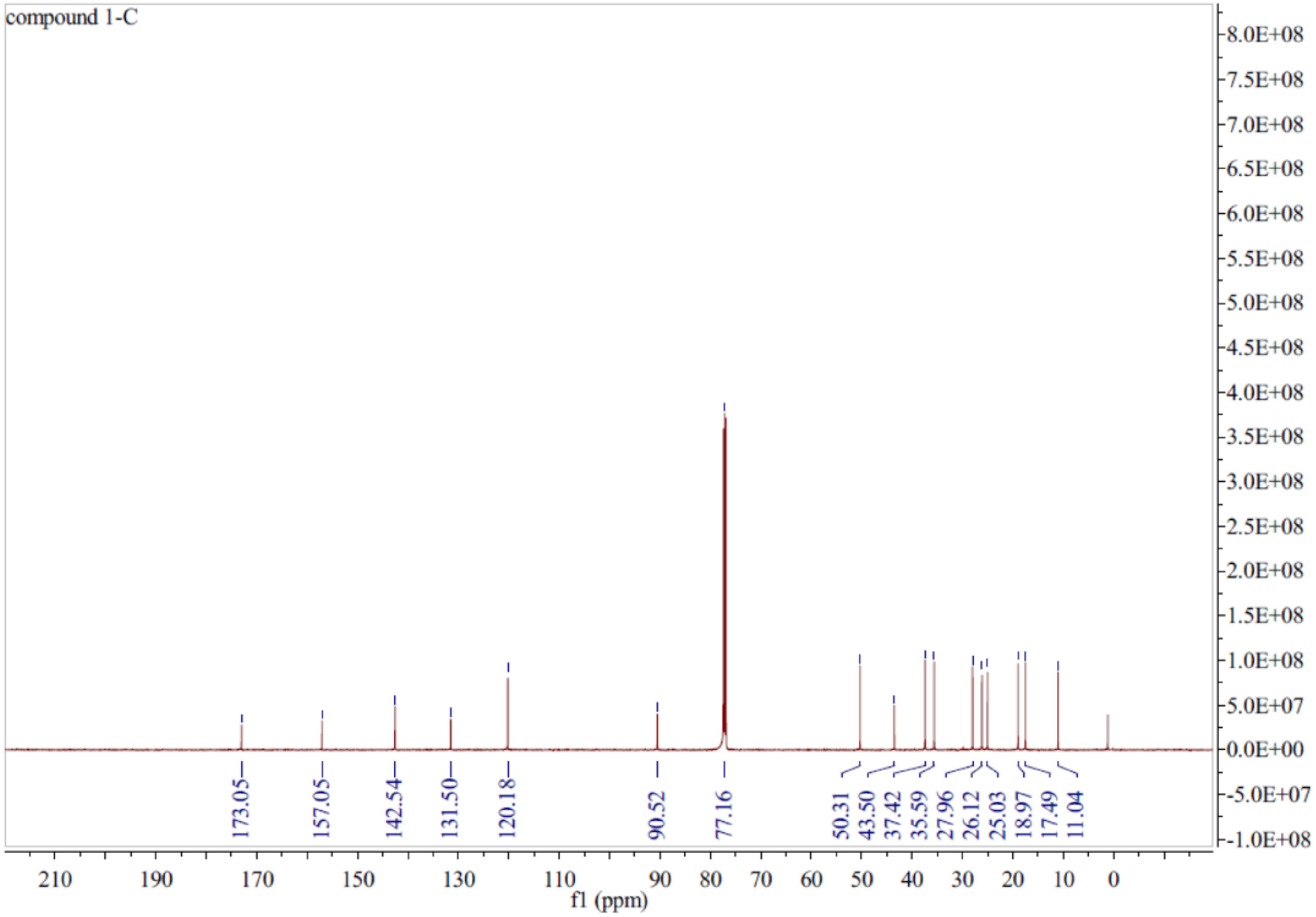

Figure S5. ^{13}C NMR spectrum of calvukoellian G (**1**) in $CDCl_3$ (125 MHz).

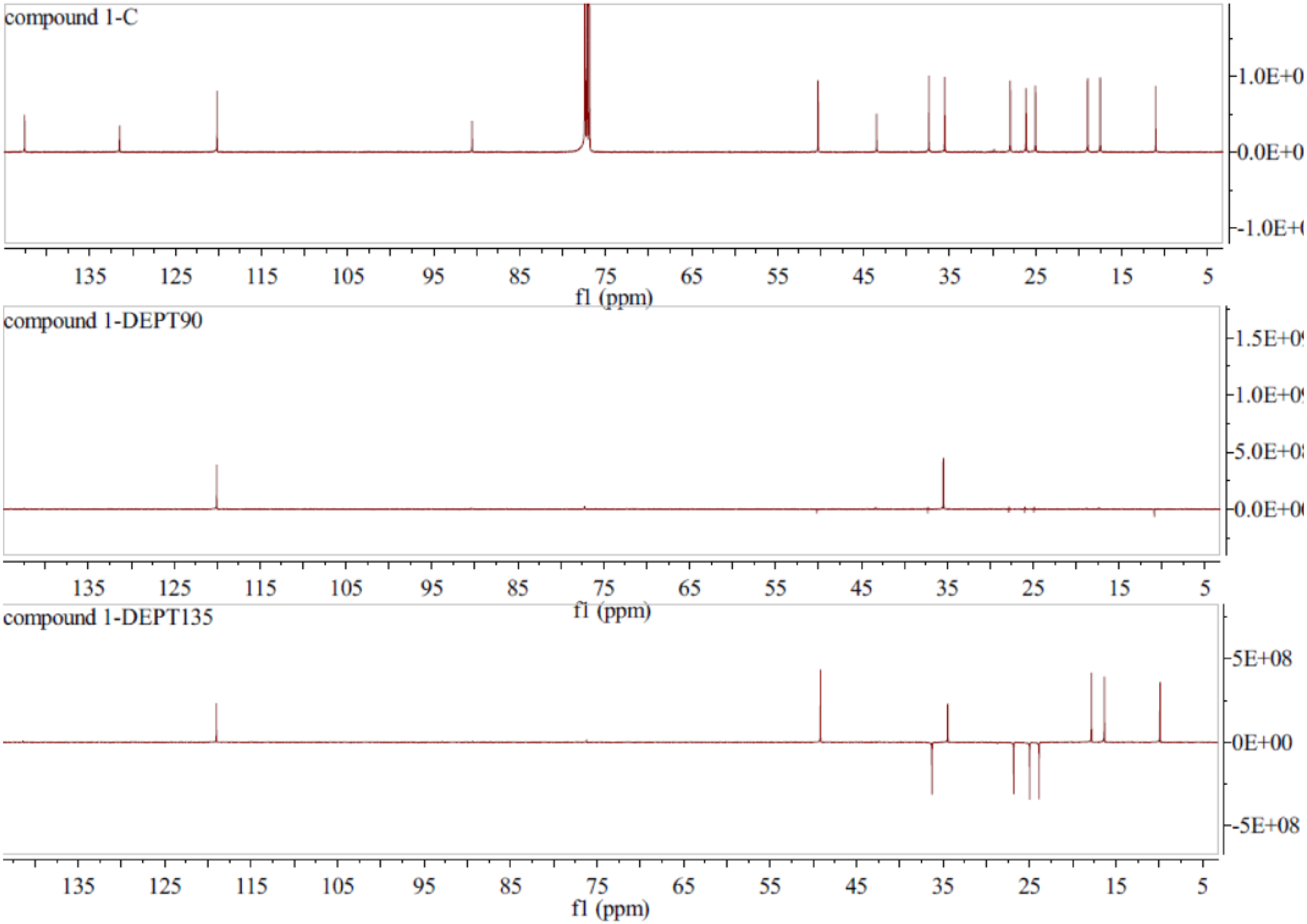

Figure S6. ^{13}C NMR and DEPT spectrum of calvukoellian G (**1**) in $CDCl_3$ (125 MHz).

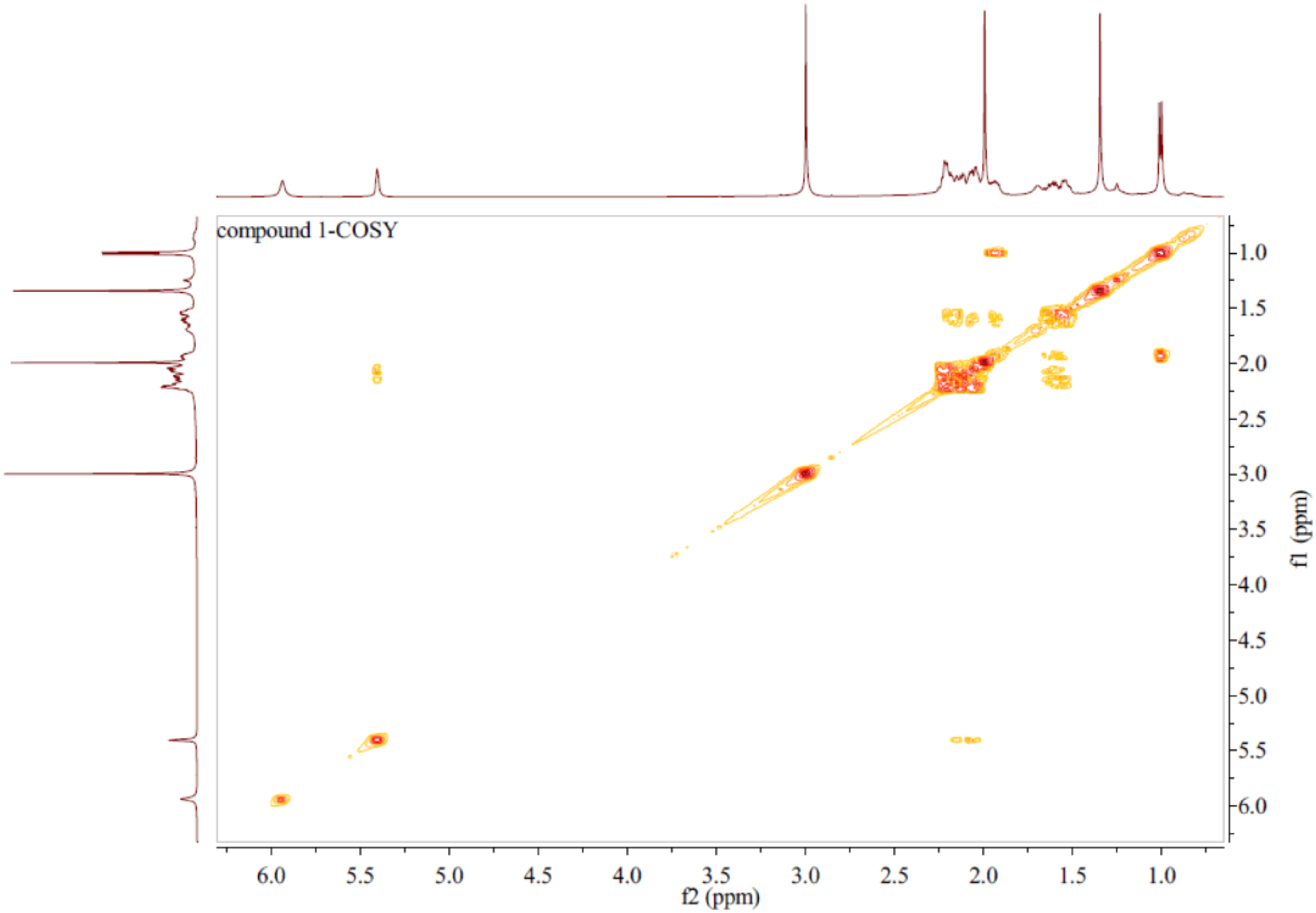

Figure S7. ^{1}H-^{1}H COSY spectrum of calvukoellian G (**1**) in $CDCl_3$ (500 MHz).

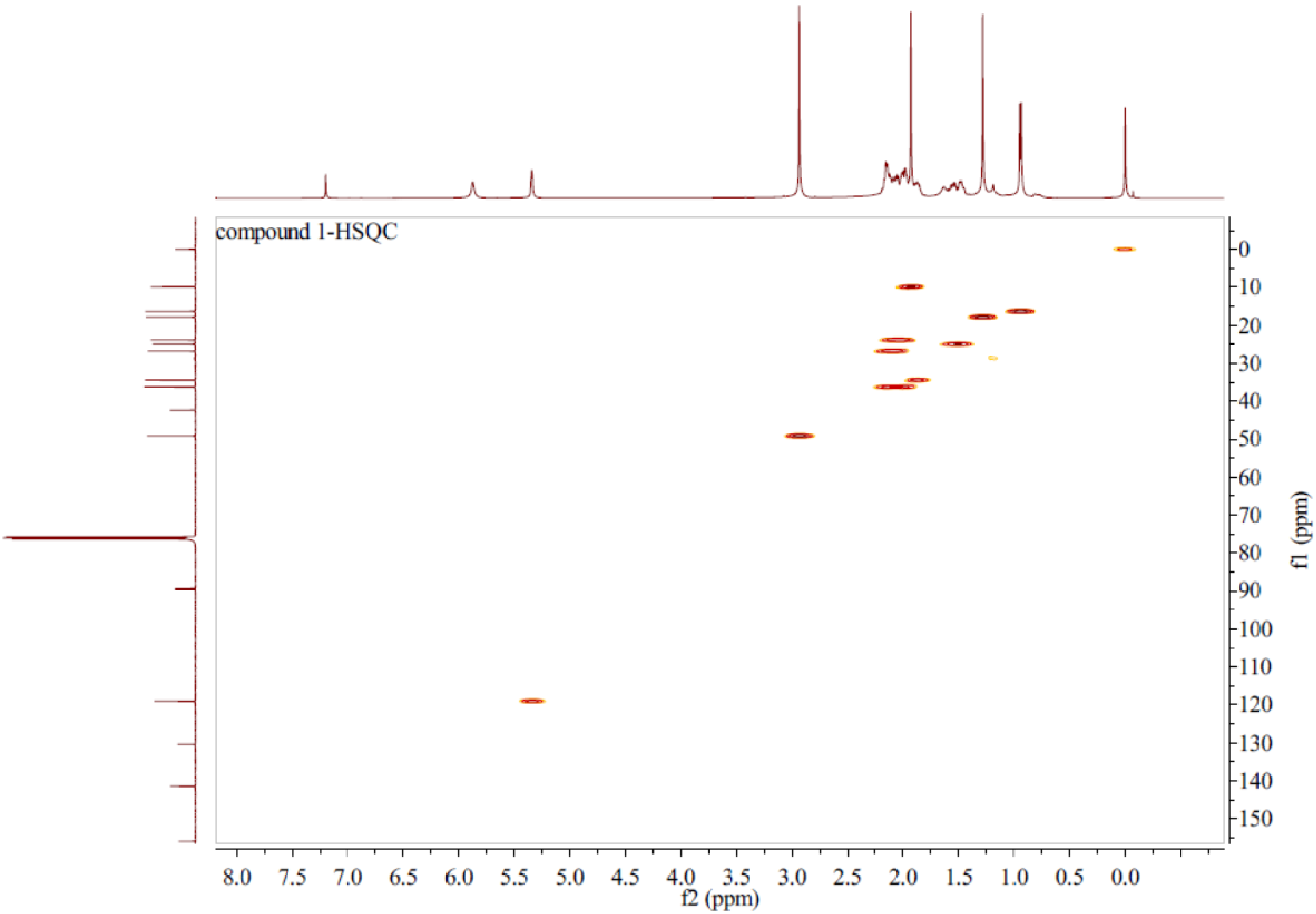

Figure S8. HSQC spectrum of calvukoellian G (**1**) in $CDCl_3$ (500 MHz).

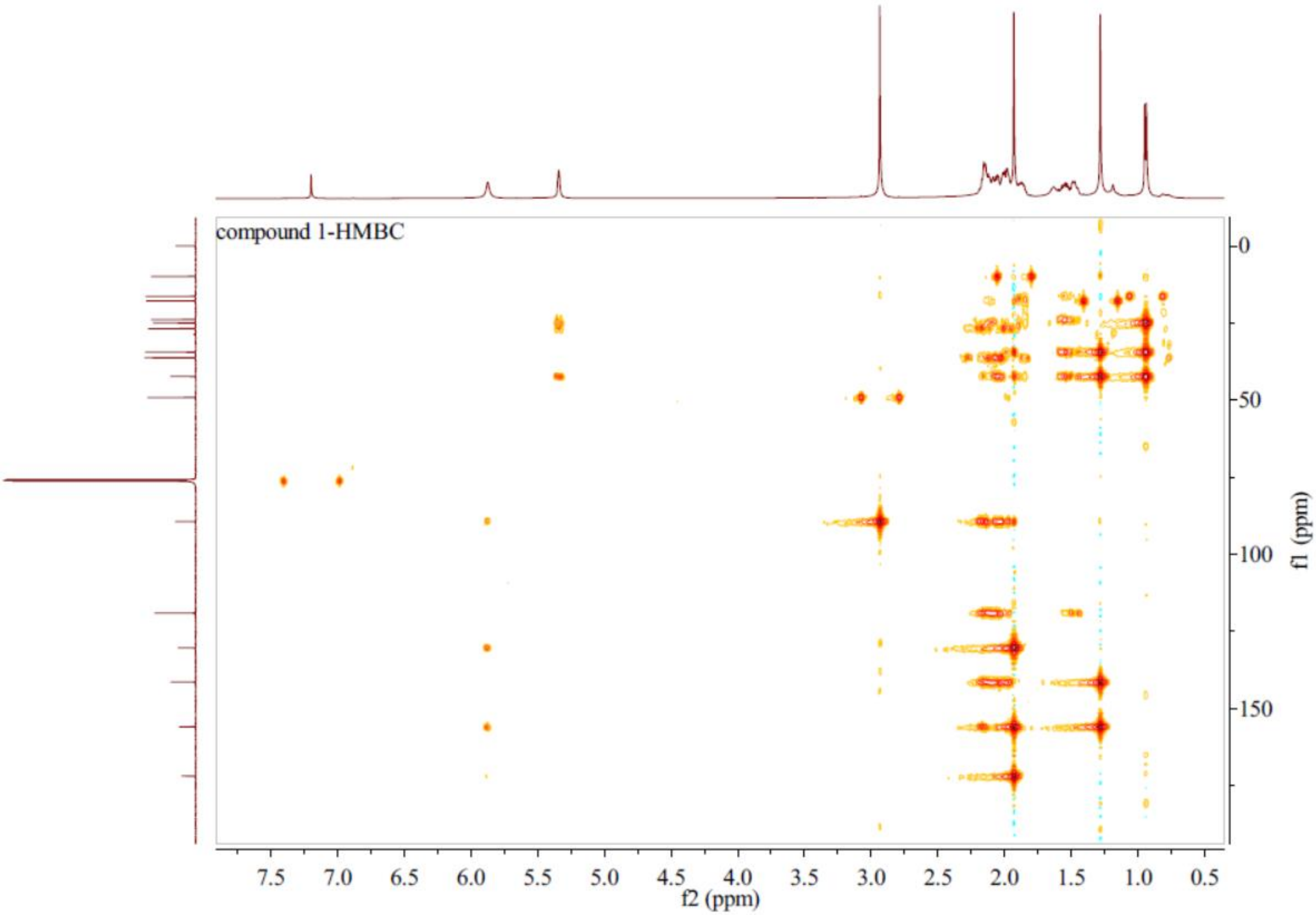

Figure S9. HMBC spectrum of calvukoellian G (**1**) in $CDCl_3$ (500 MHz).

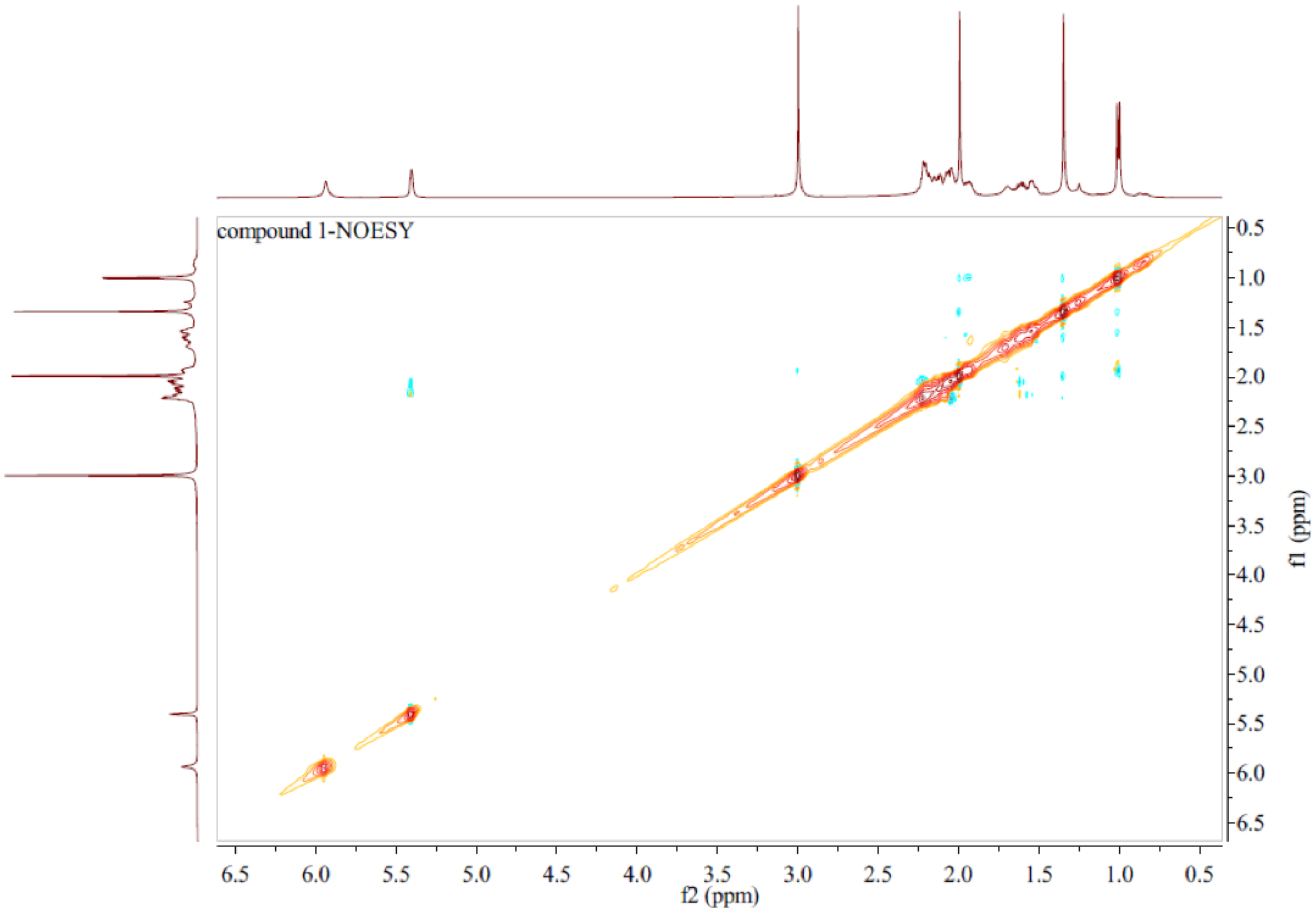

Figure S10. NOESY spectrum of calvukoellian G (**1**) in $CDCl_3$ (500 MHz).

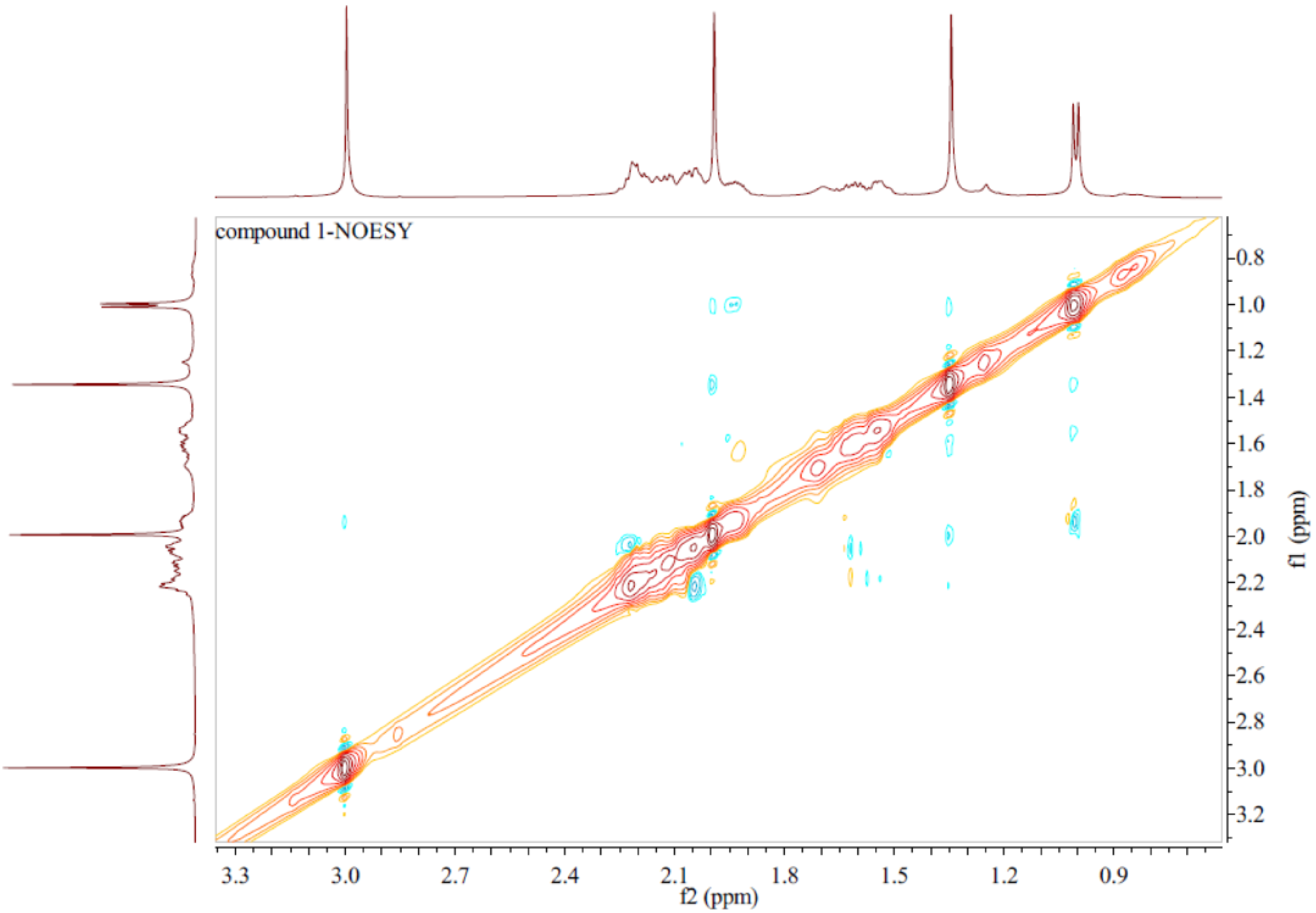

Figure S11. NOESY spectrum of calvukoellian G (**1**) in $CDCl_3$ (500 MHz).

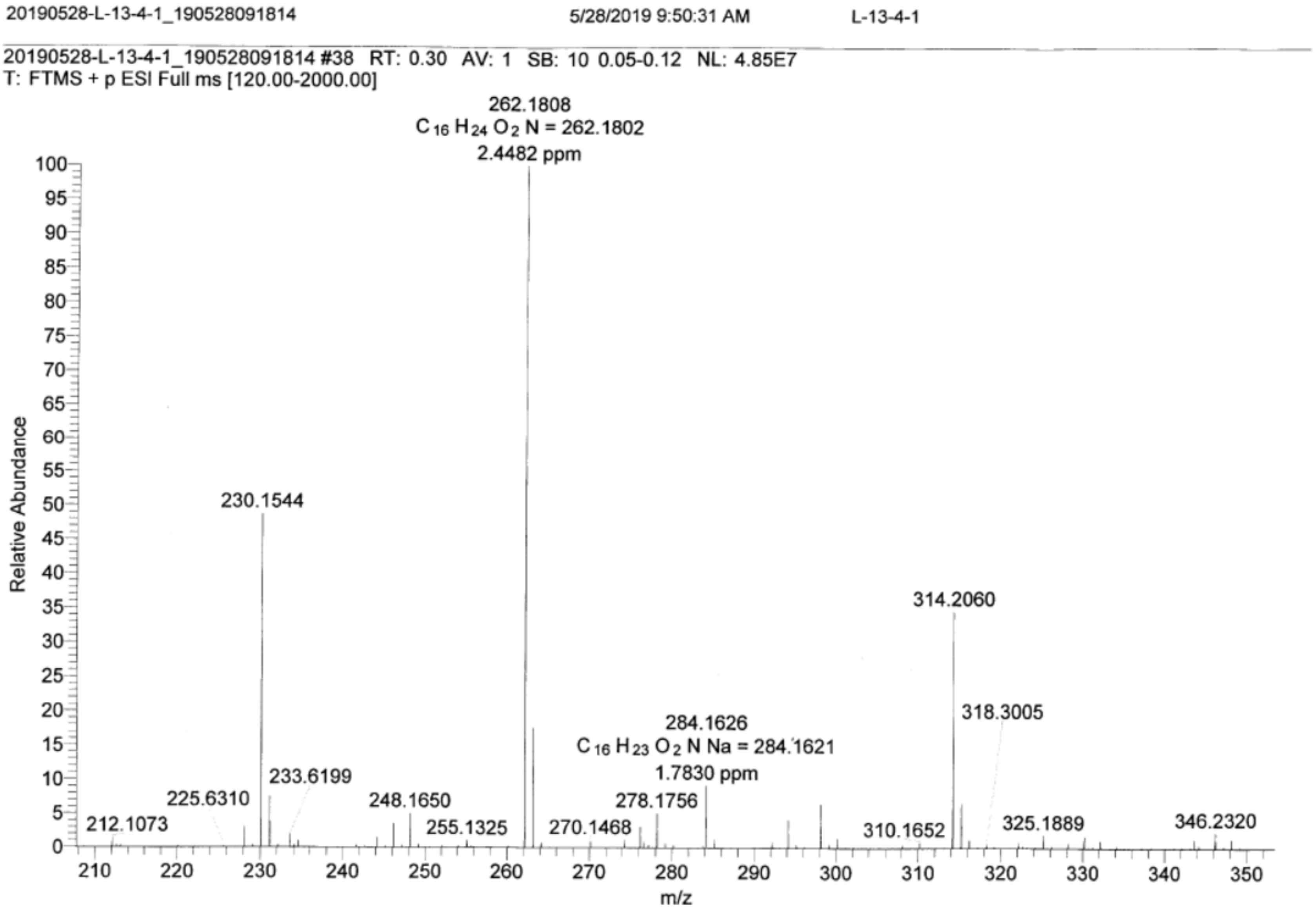

Figure S12. HRESIMS data of calvukoellian G (**1**).

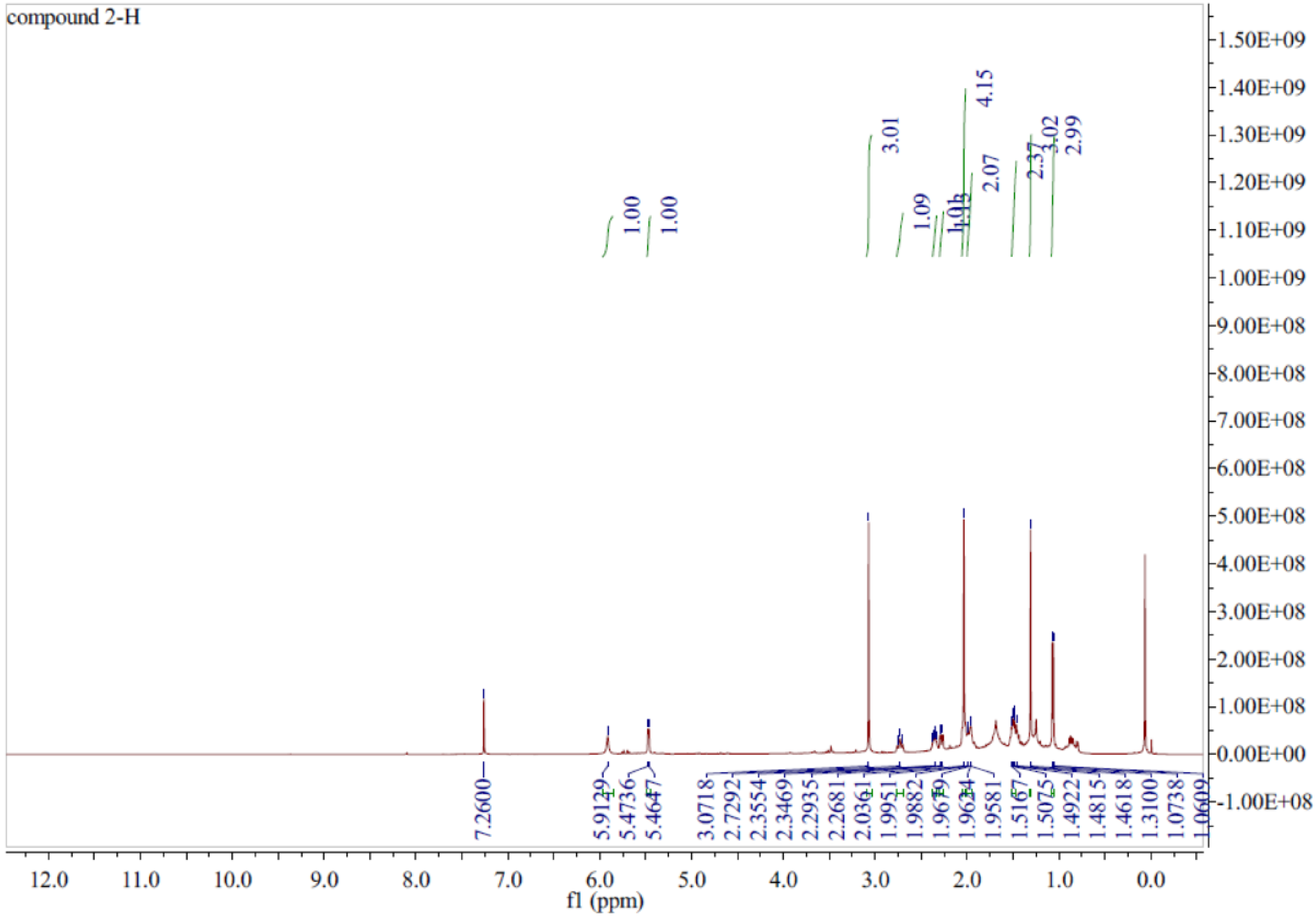

Figure S13. ^{1}H NMR spectrum of calvukoellian H (**2**) in $CDCl_3$ (500 MHz).

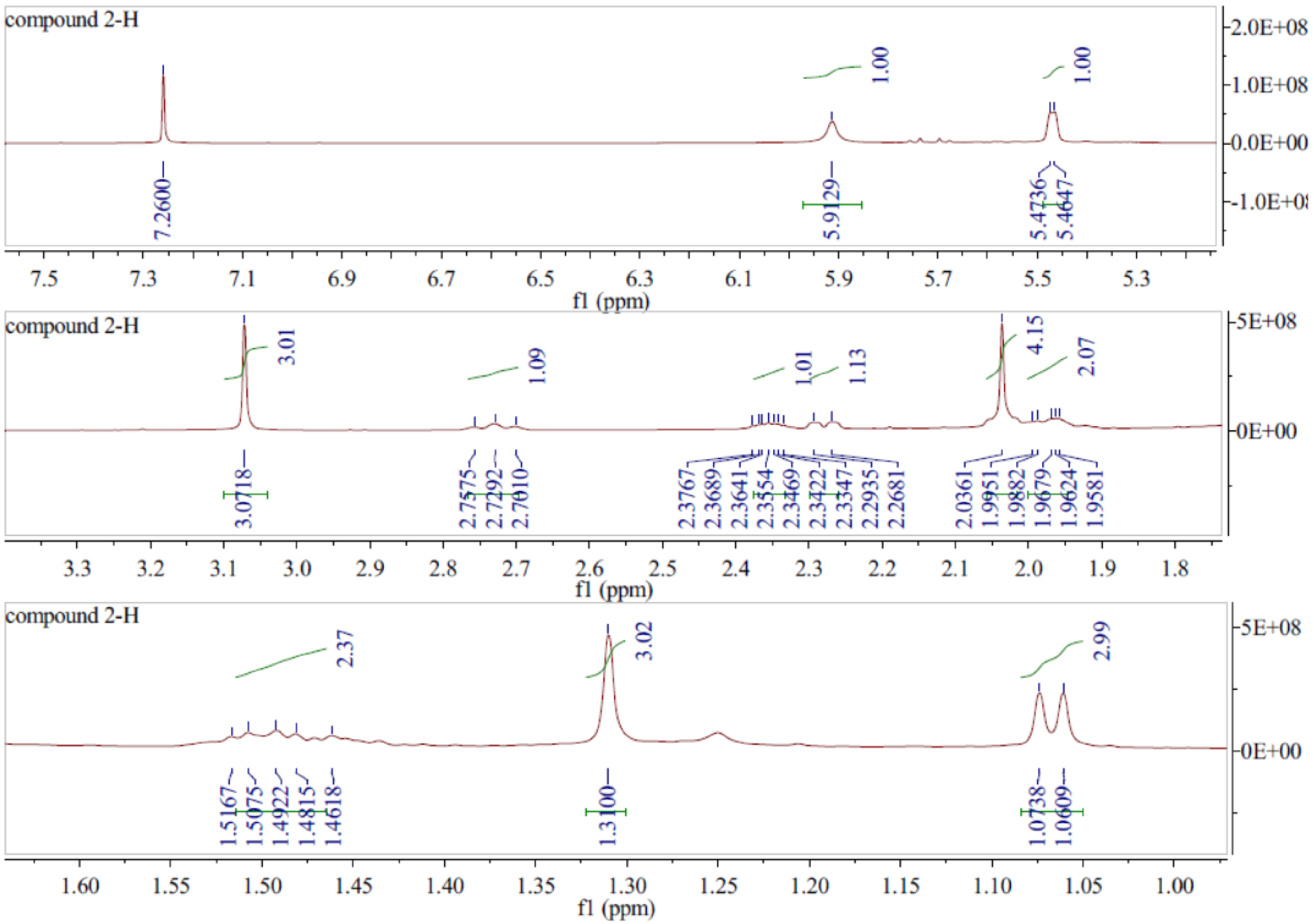

Figure S14. Enlarged ^{1}H NMR spectrum of calvukoellian H (**2**) in $CDCl_3$ (500 MHz).

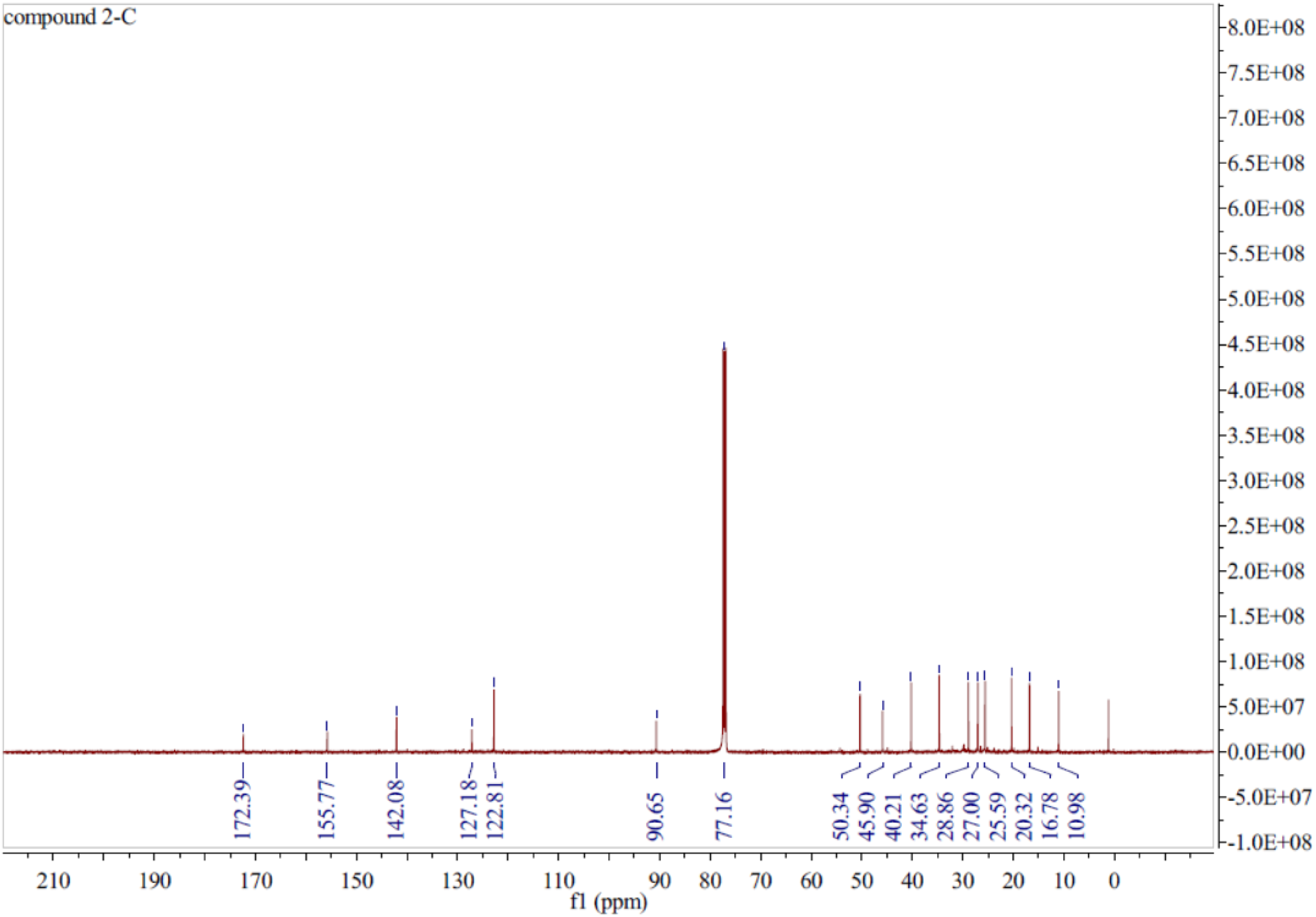

Figure S15. ^{13}C NMR spectrum of calvukoellian H (**2**) in $CDCl_3$ (125 MHz).

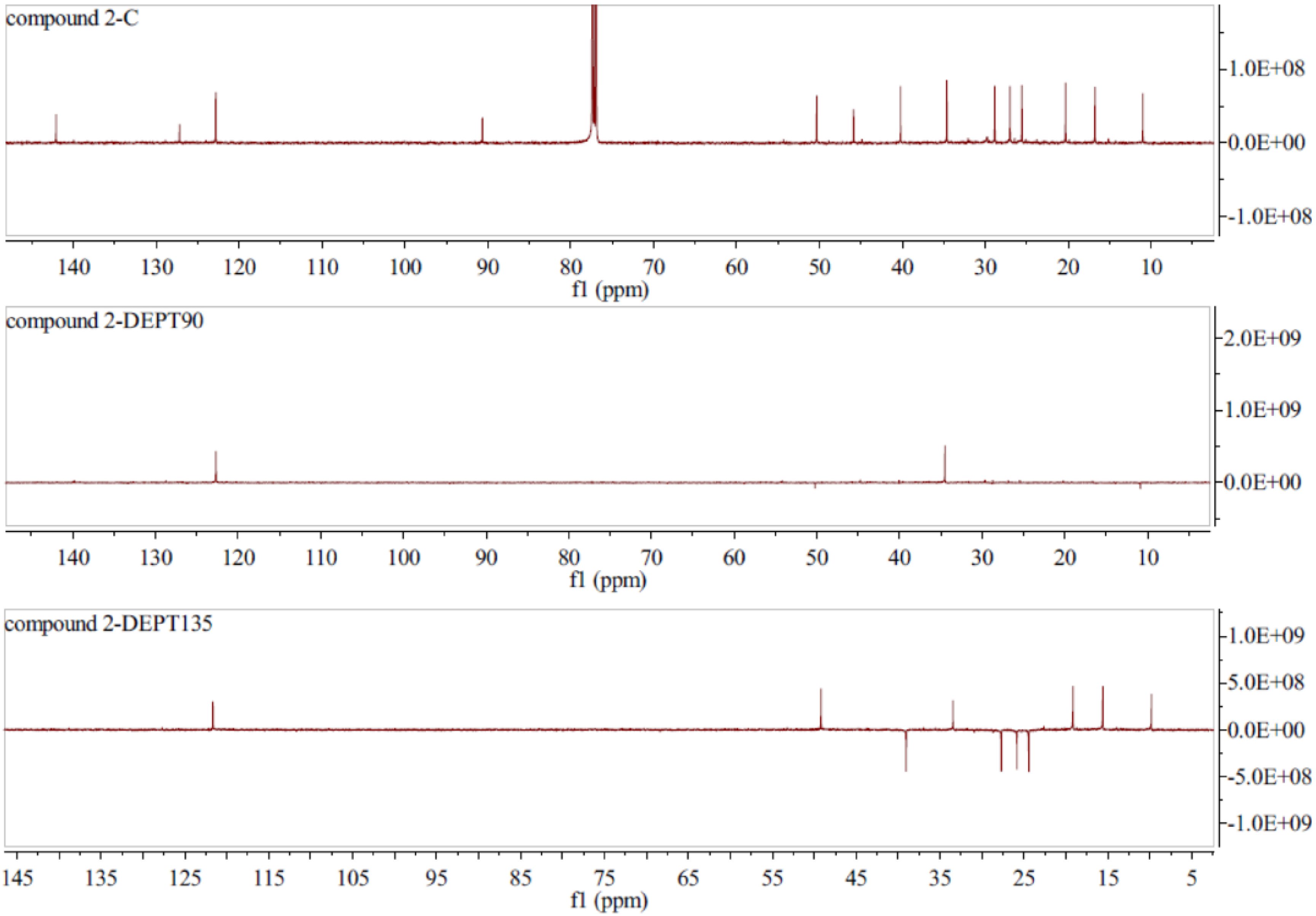

Figure S16. ^{13}C NMR and DEPT spectrum of calvukoellian H (**2**) in $CDCl_3$ (125 MHz).

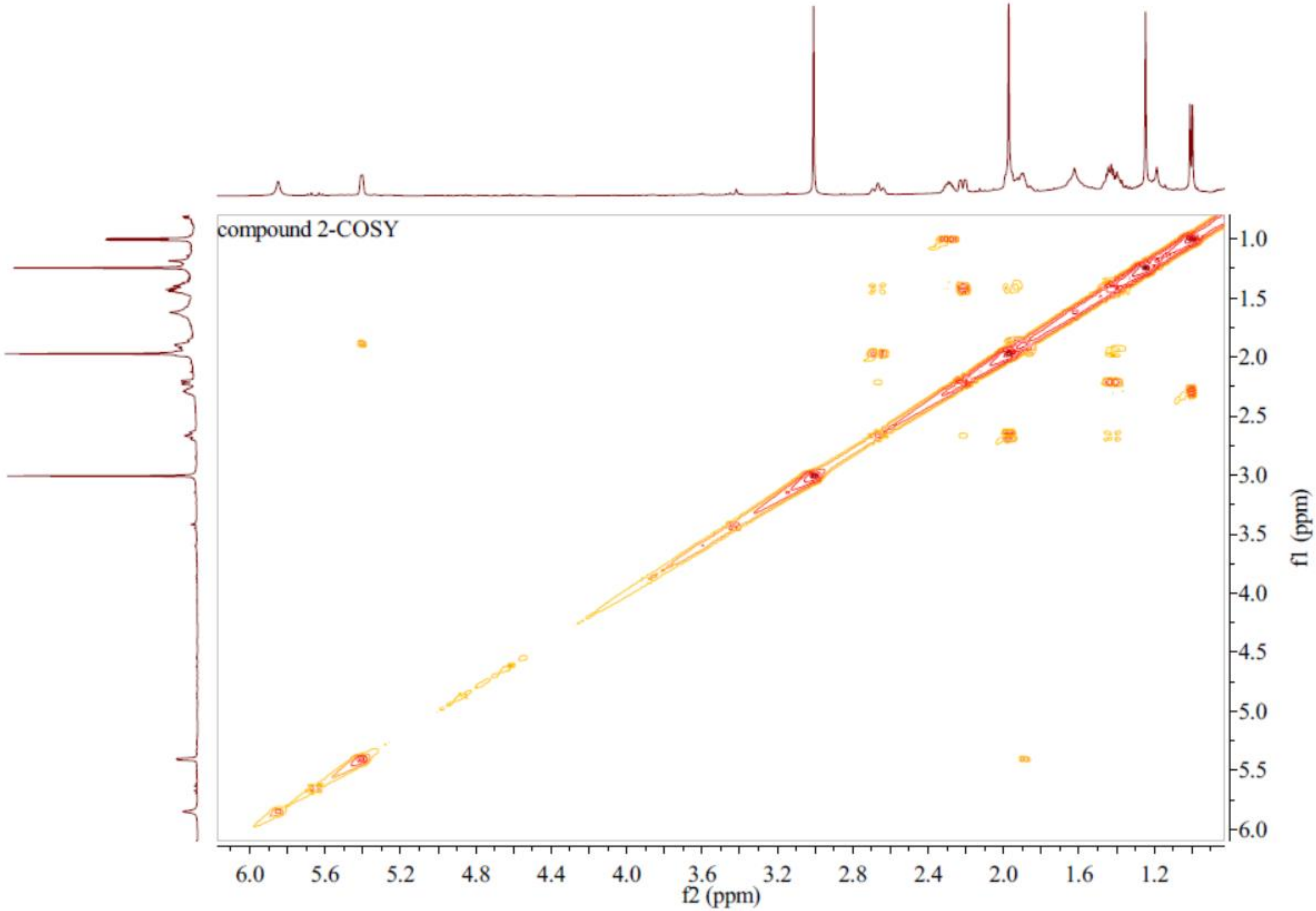

Figure S17. ^{1}H-^{1}H COSY spectrum of calvukoellian H (**2**) in $CDCl_3$ (500 MHz).

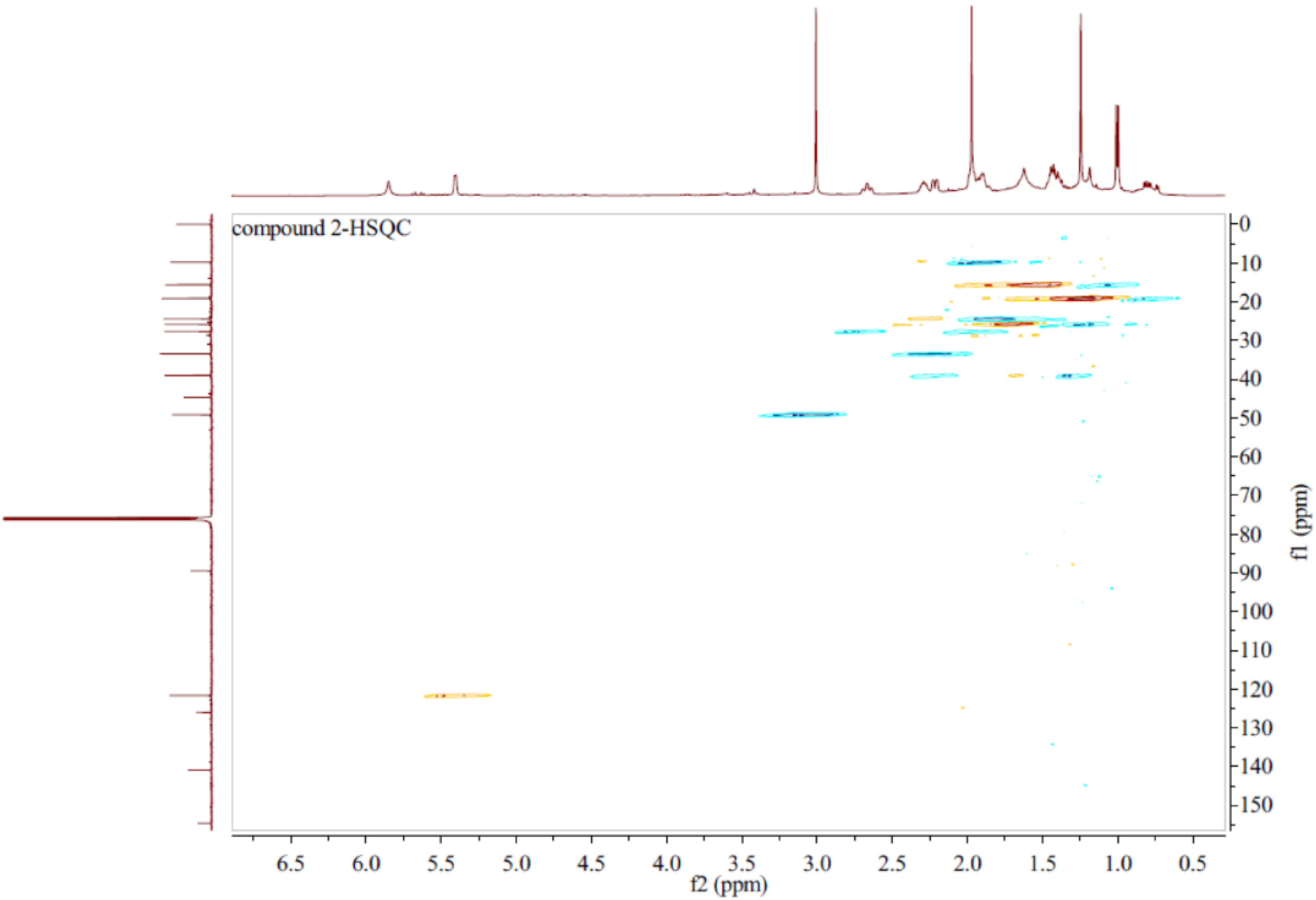

Figure S18. HSQC spectrum of calvukoellian H (**2**) in $CDCl_3$ (500 MHz).

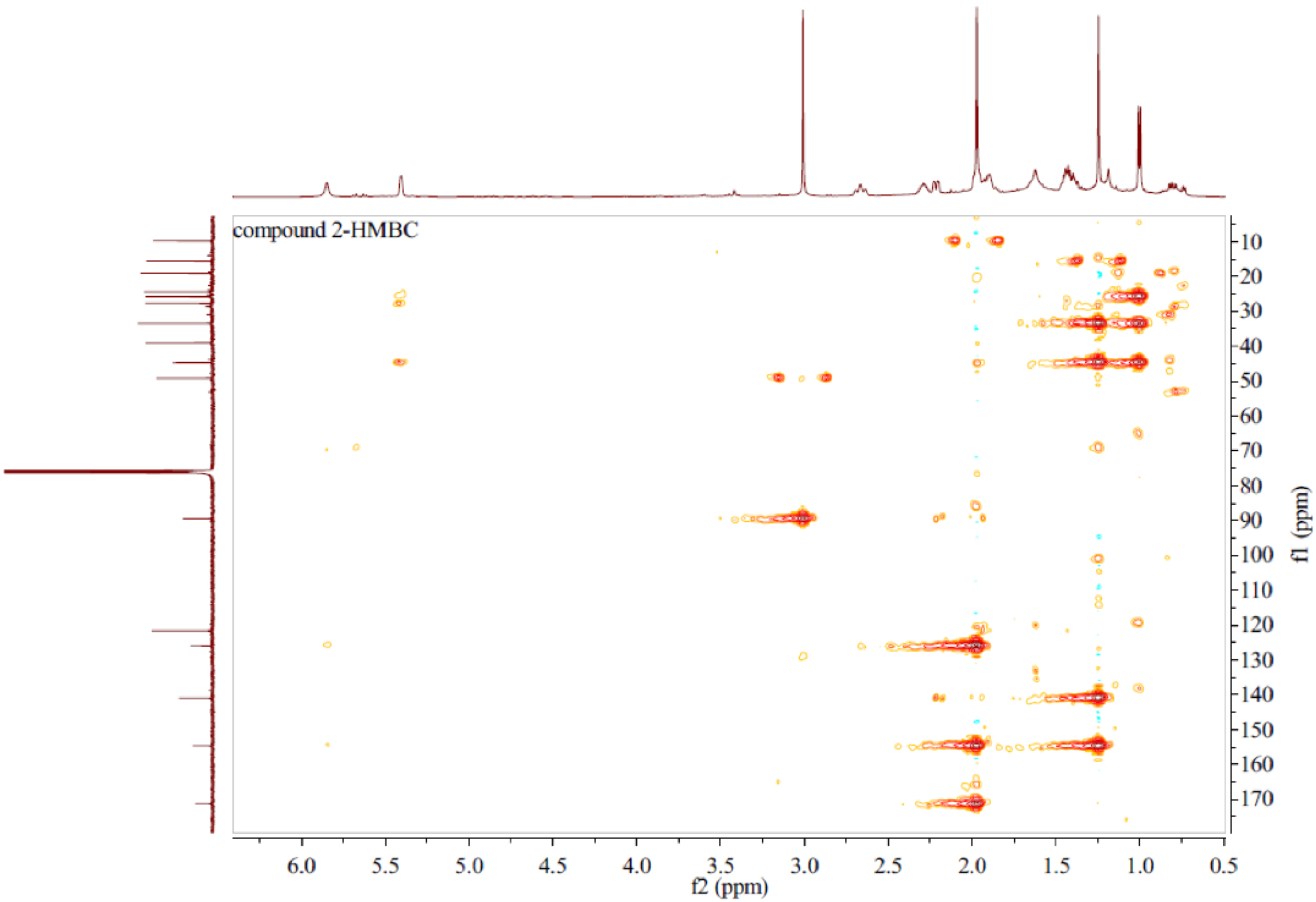

Figure S19. HMBC spectrum of calvukoellian H (**2**) in $CDCl_3$ (500 MHz).

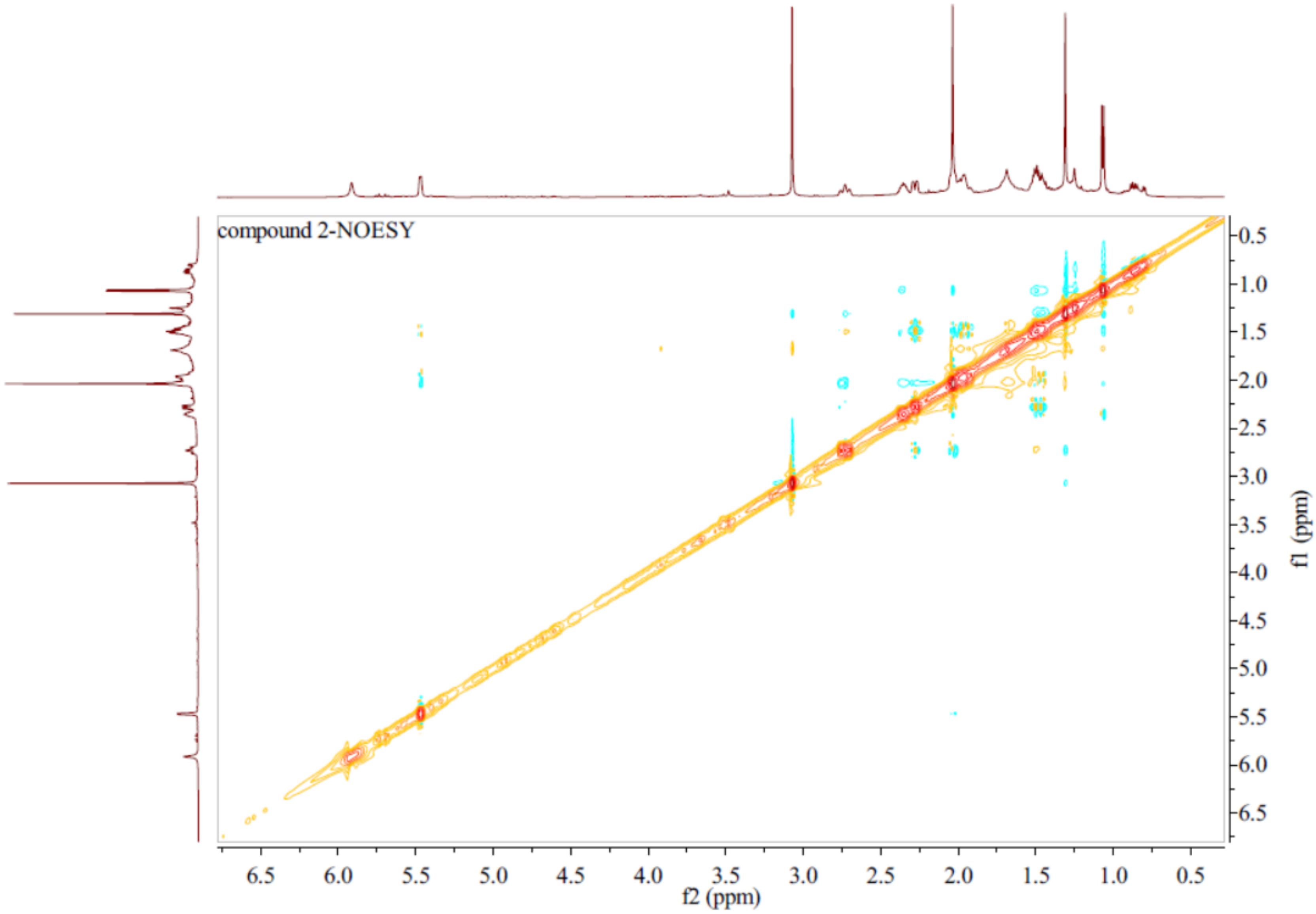

Figure S20. NOESY spectrum of calvukoellian H (**2**) in $CDCl_3$ (500 MHz).

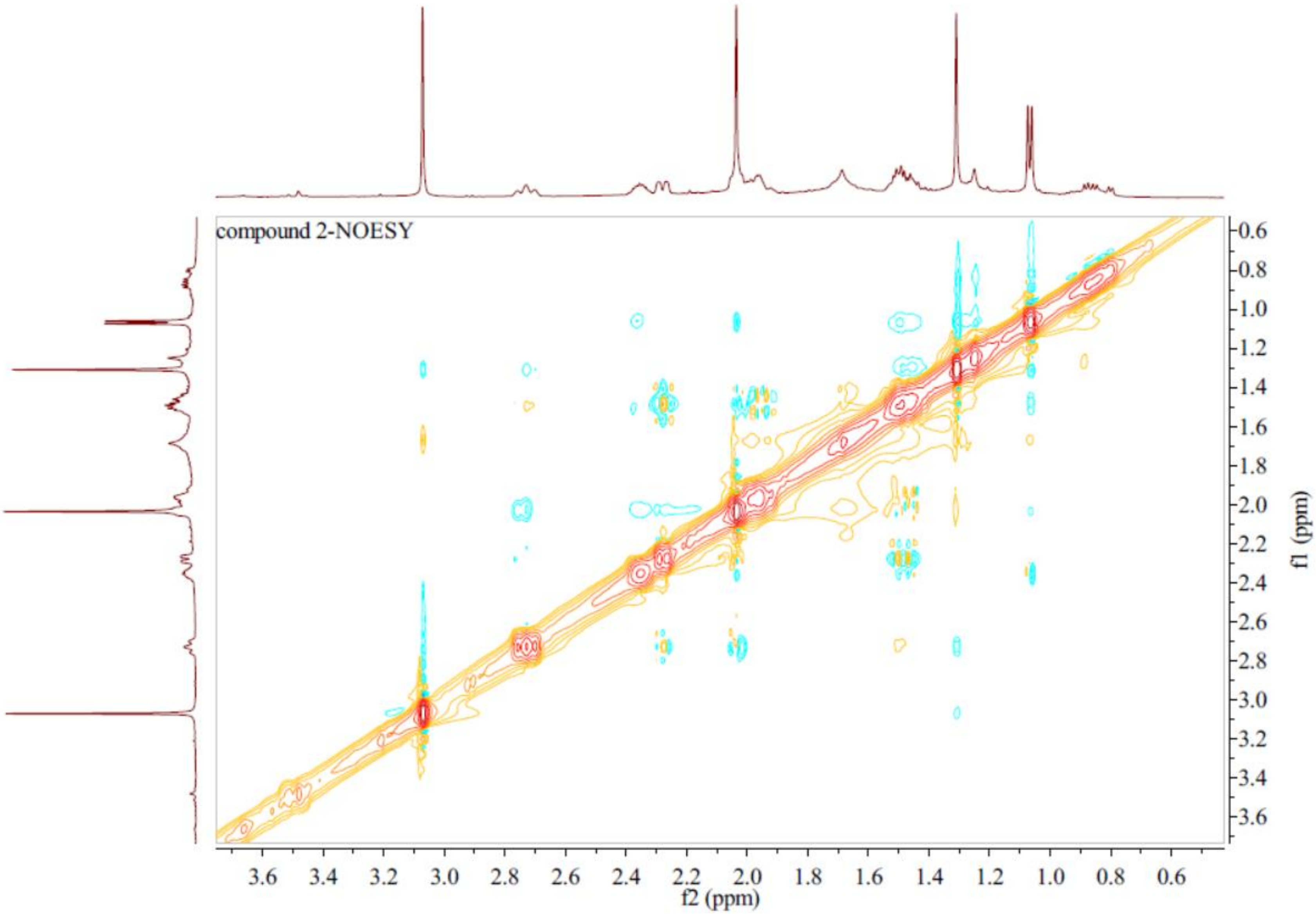

Figure S21. NOESY spectrum of calvukoellian H (**2**) in $CDCl_3$ (500 MHz).

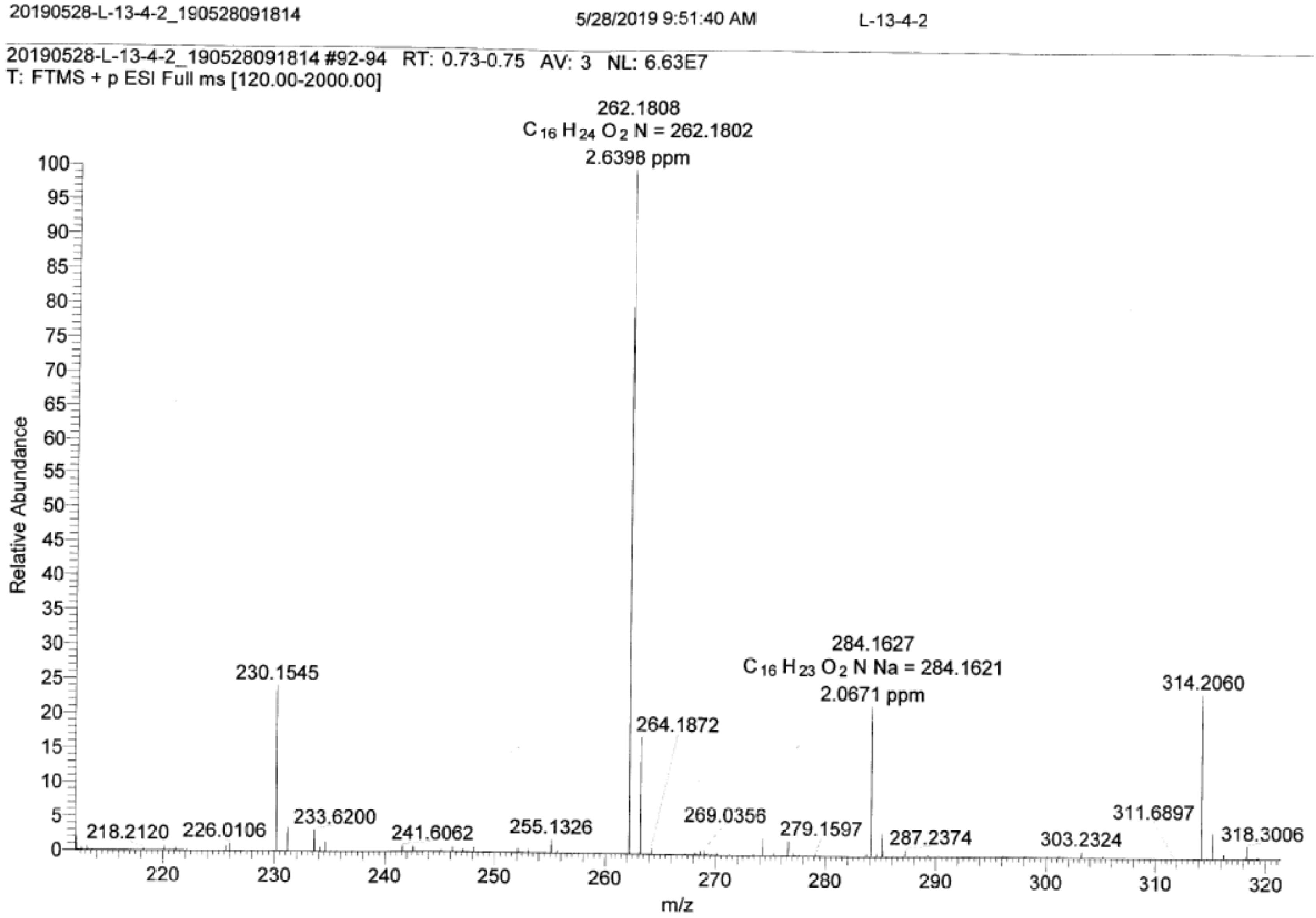

Figure S22. HRESIMS data of calvukoellian H (**2**).

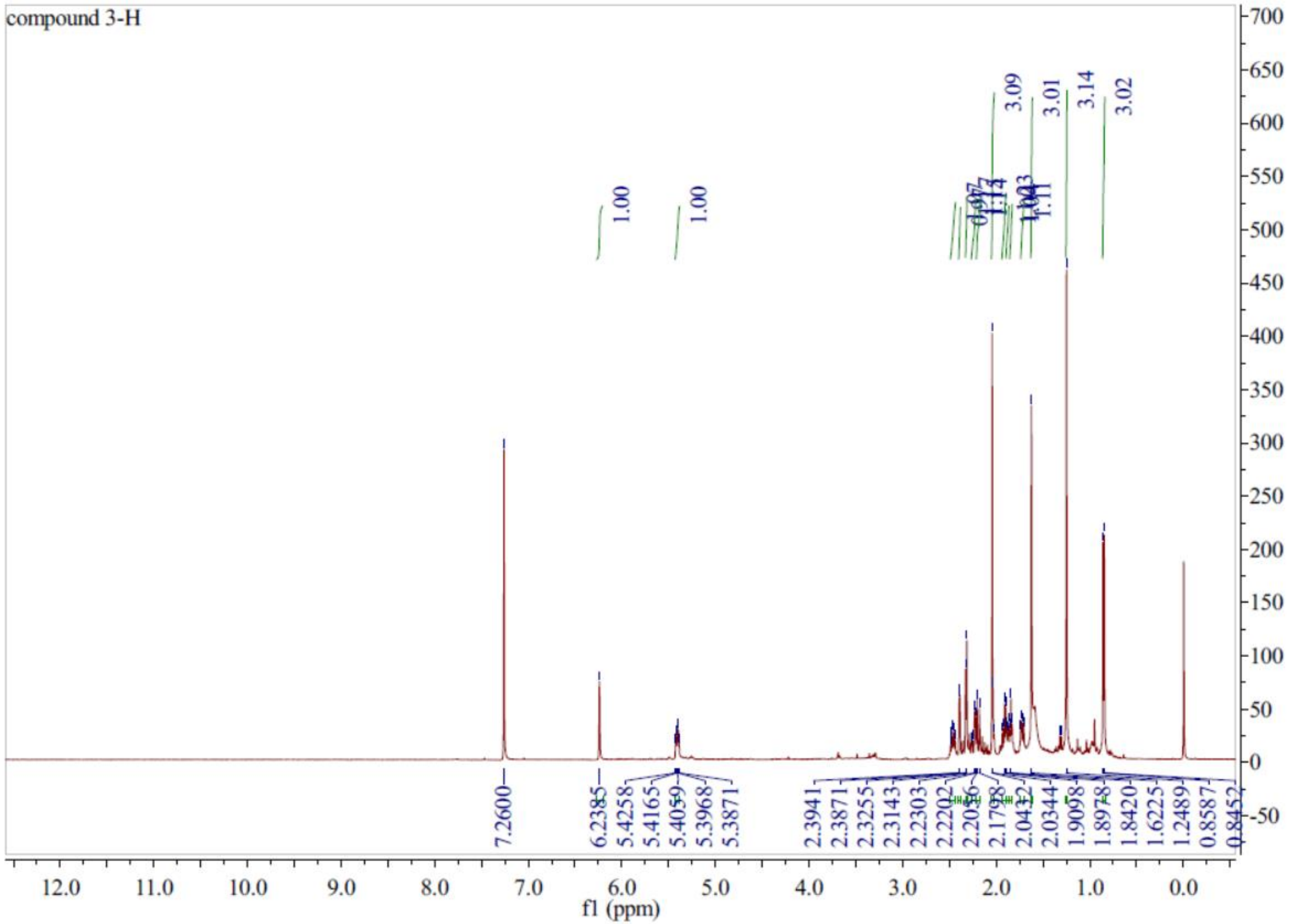

Figure S23. ^{1}H NMR spectrum of calvukoellian I (**3**) in $CDCl_3$ (500 MHz).

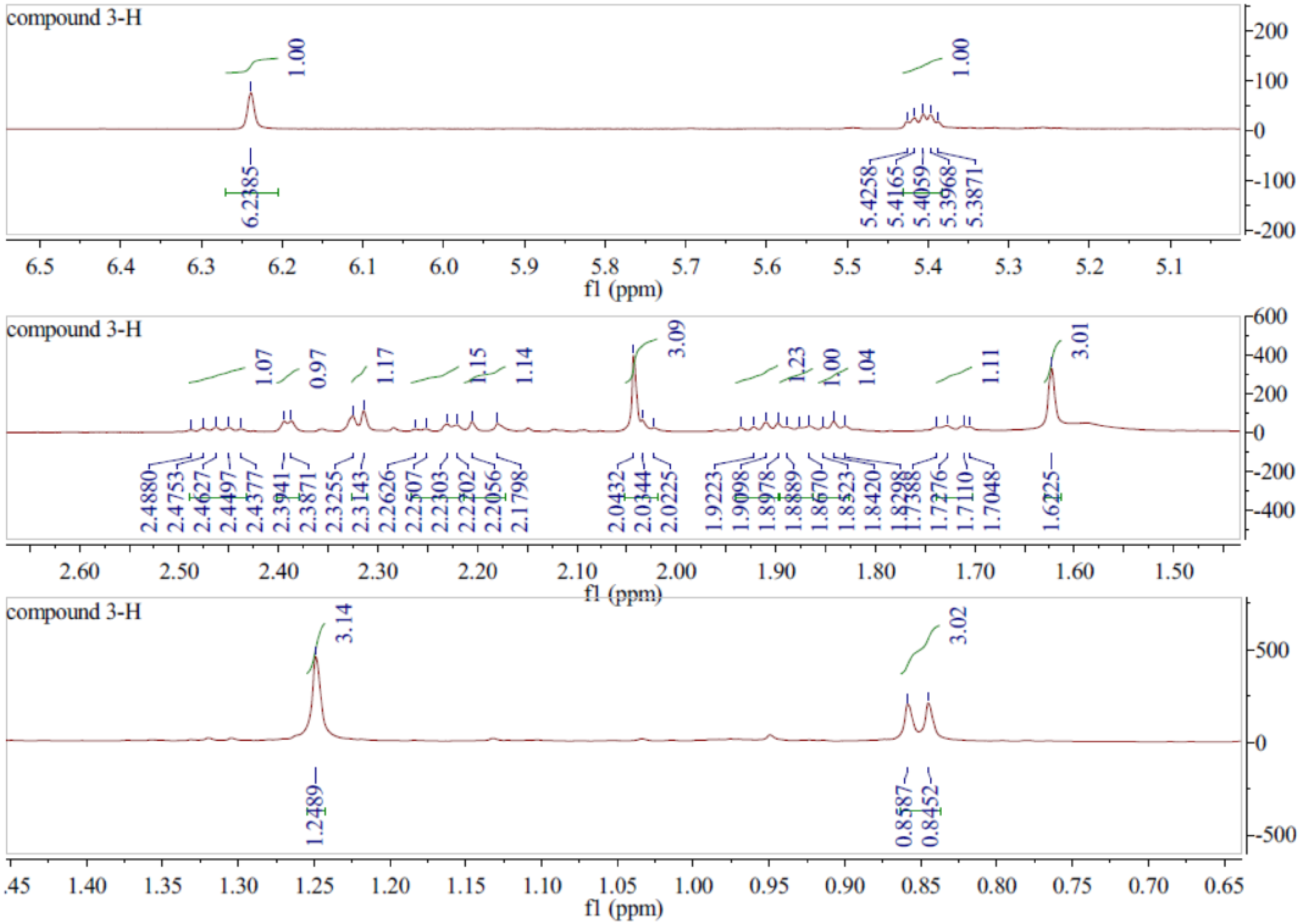

Figure S24. Enlarged ^{1}H NMR spectrum of calvukoellian I (**3**) in $CDCl_3$ (500 MHz).

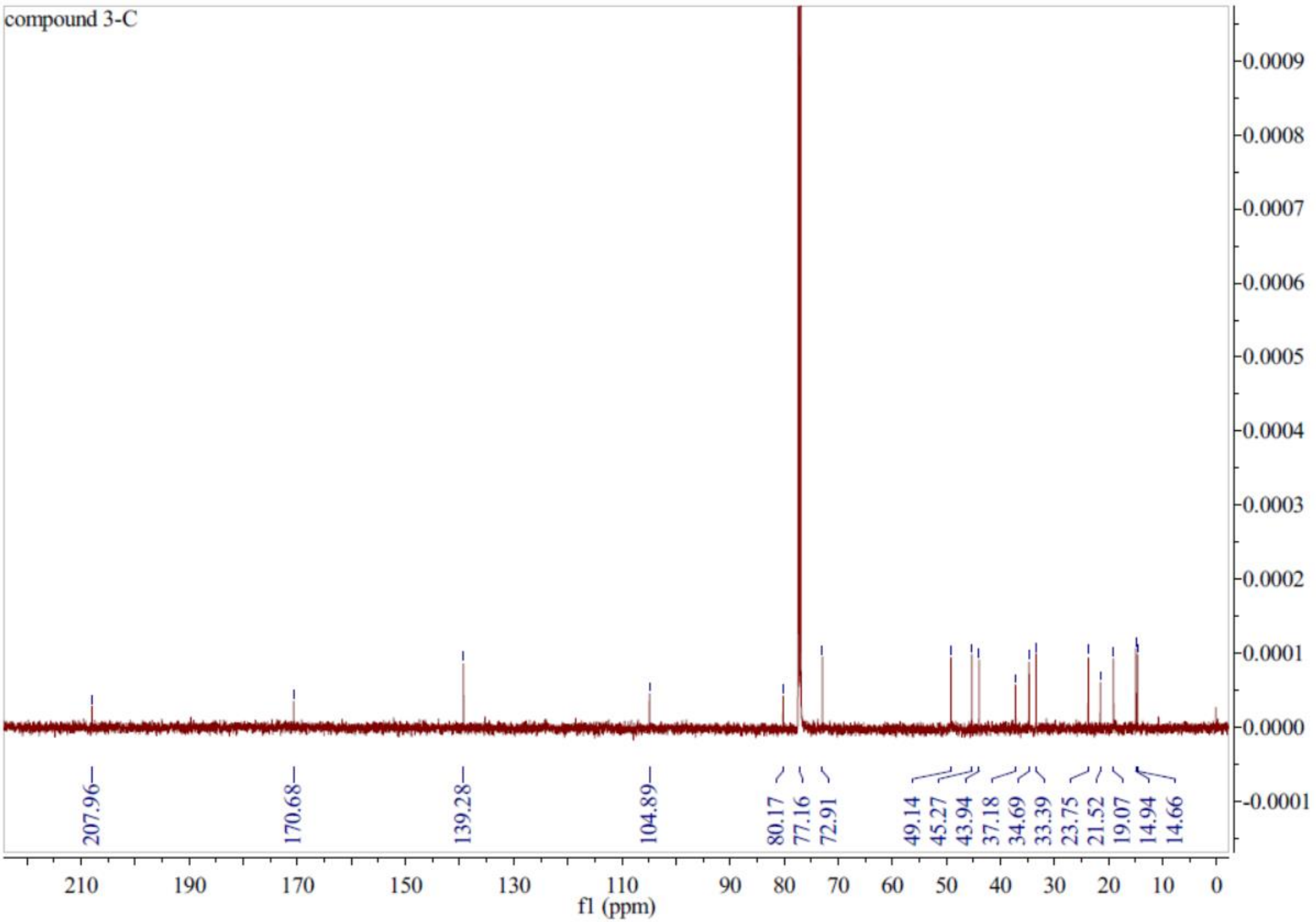

Figure S25. ^{13}C NMR spectrum of calvukoellian I (**3**) in $CDCl_3$ (125 MHz).

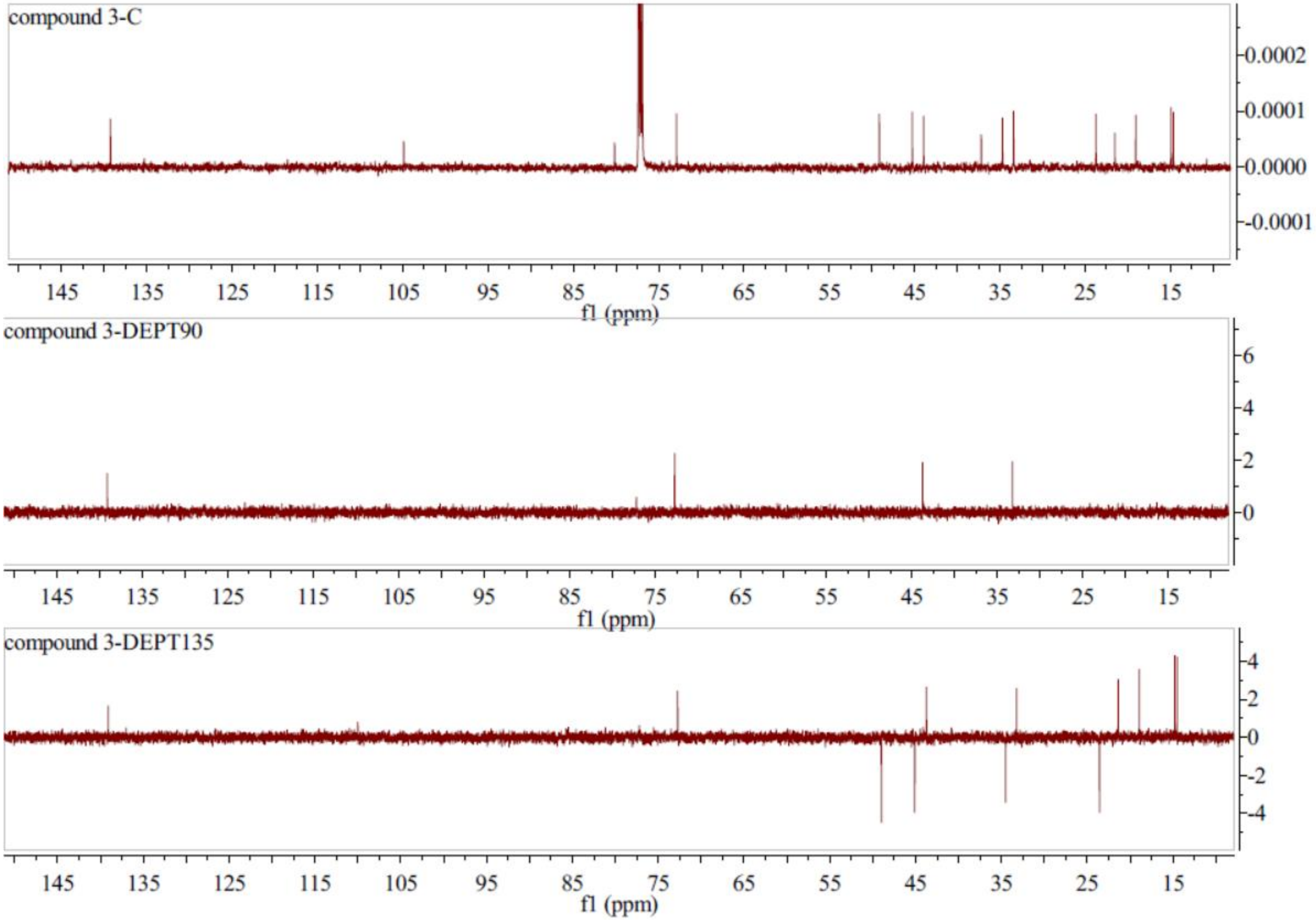

Figure S26. ^{13}C NMR and DEPT spectrum of calvukoellian I (**3**) in $CDCl_3$ (125 MHz).

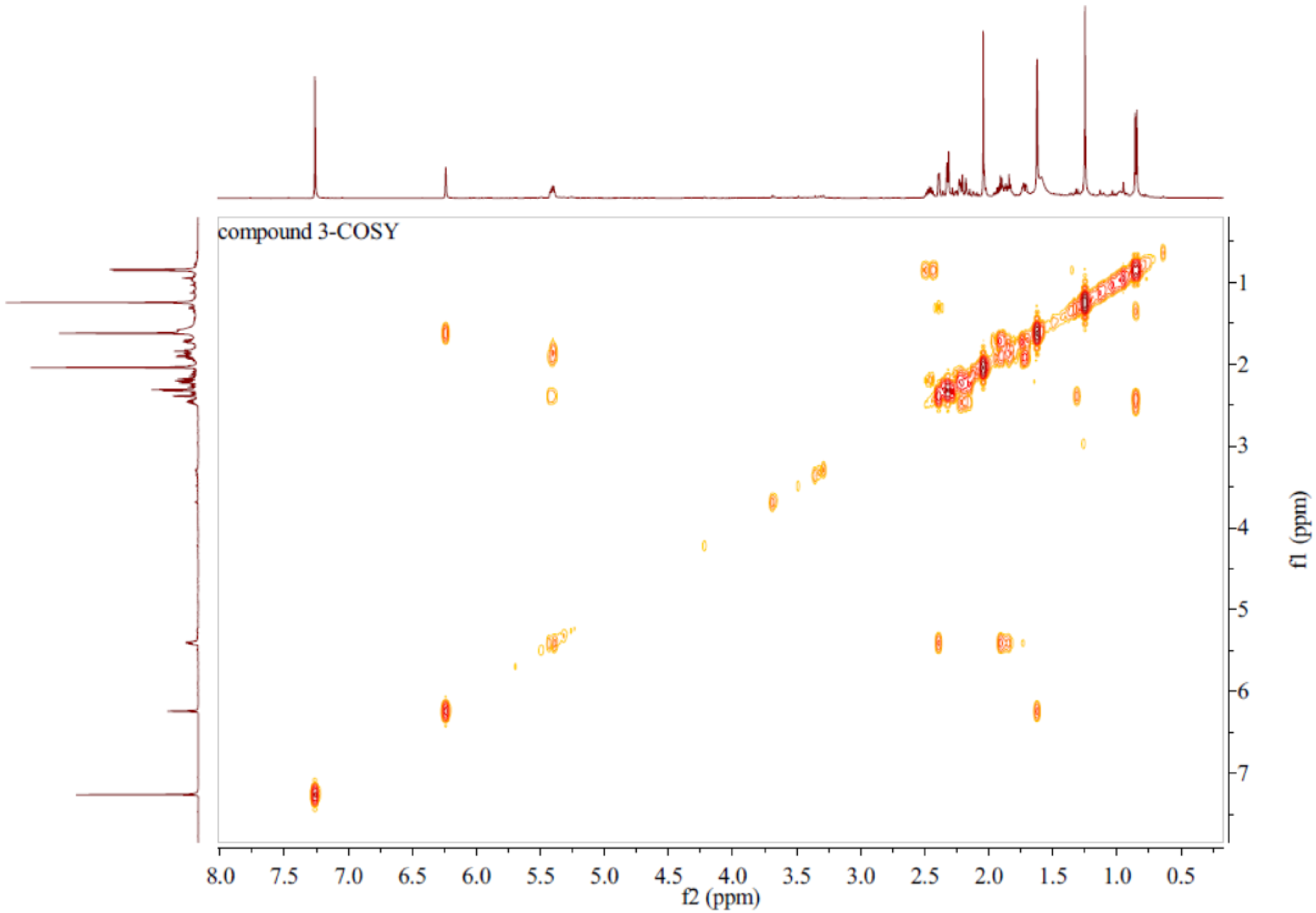

Figure S27. ^{1}H-^{1}H COSY spectrum of calvukoellian I (**3**) in $CDCl_3$ (500 MHz).

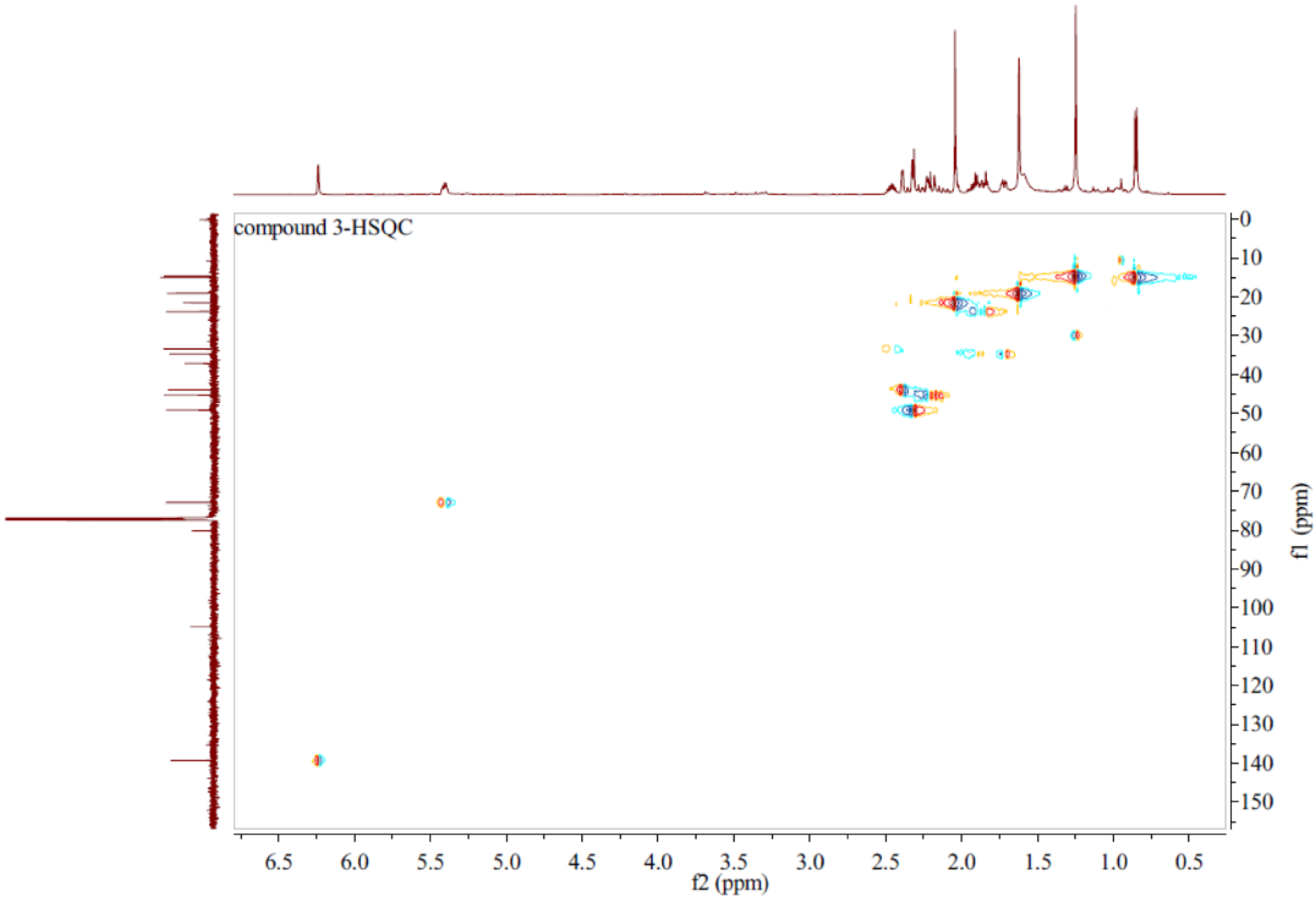

Figure S28. HSQC spectrum of calvukoellian I (**3**) in $CDCl_3$ (500 MHz).

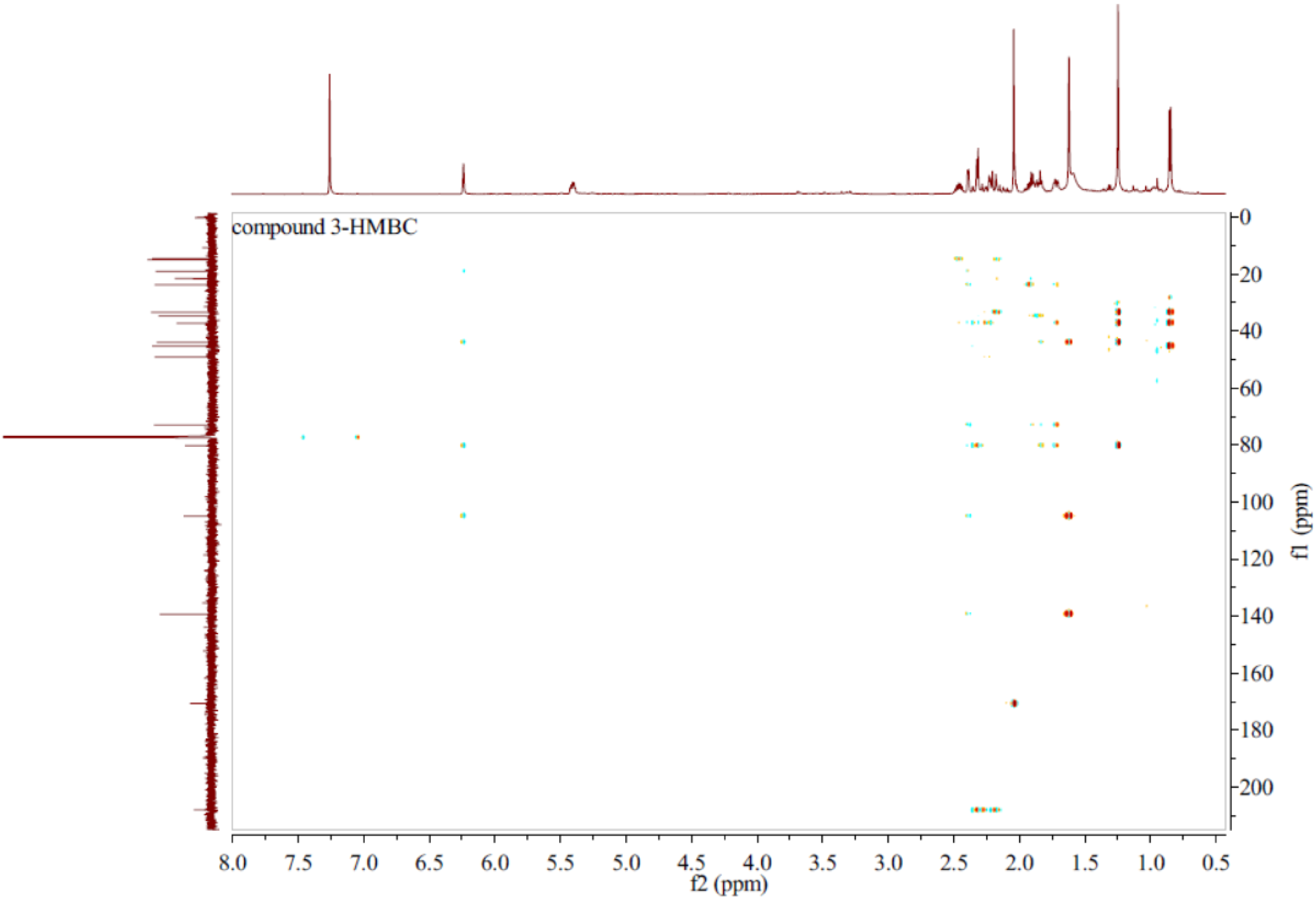

Figure S29. HMBC spectrum of calvukoellian I (**3**) in $CDCl_3$ (500 MHz).

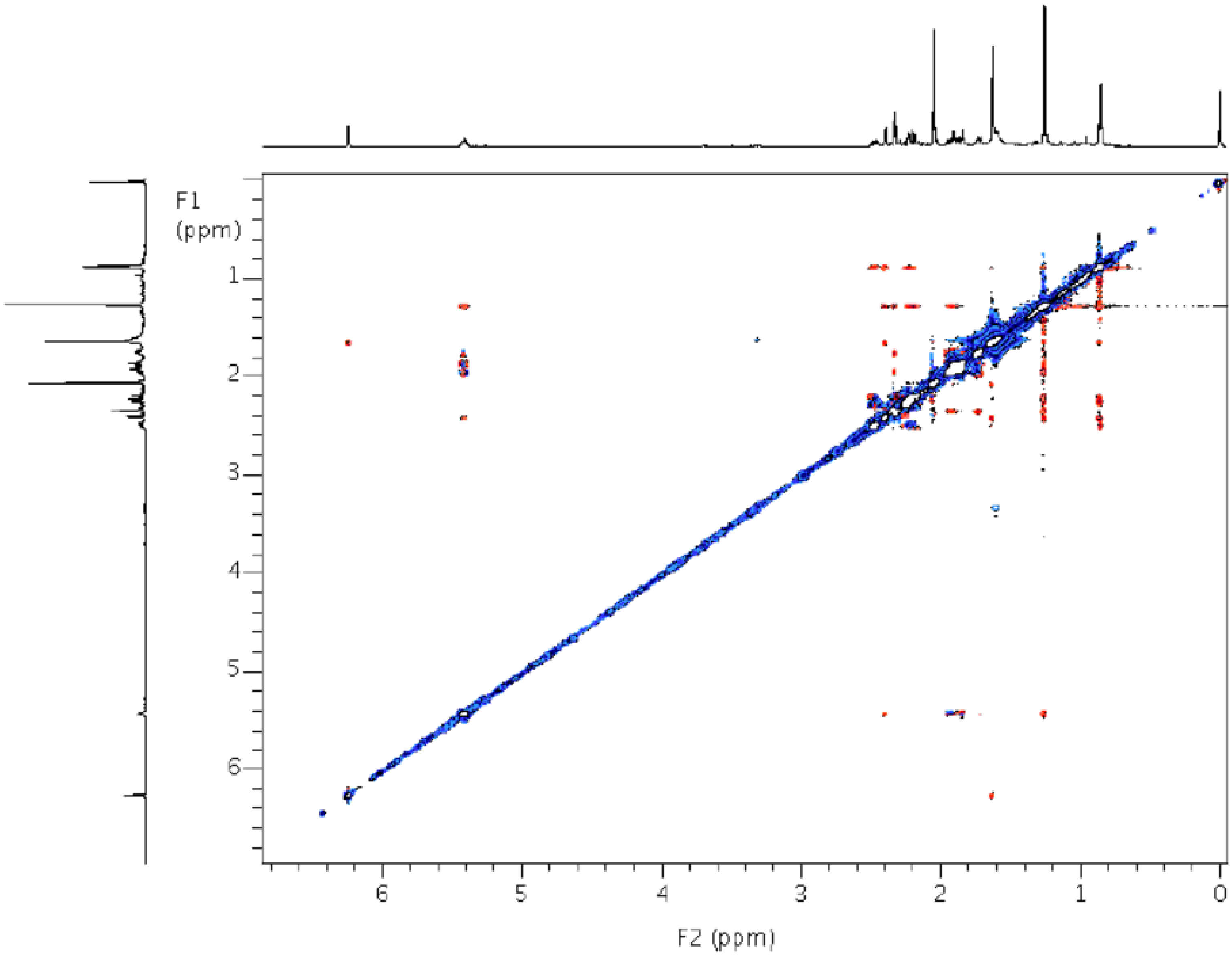

Figure S30. NOESY spectrum of calvukoellian I (**3**) in $CDCl_3$ (500 MHz).

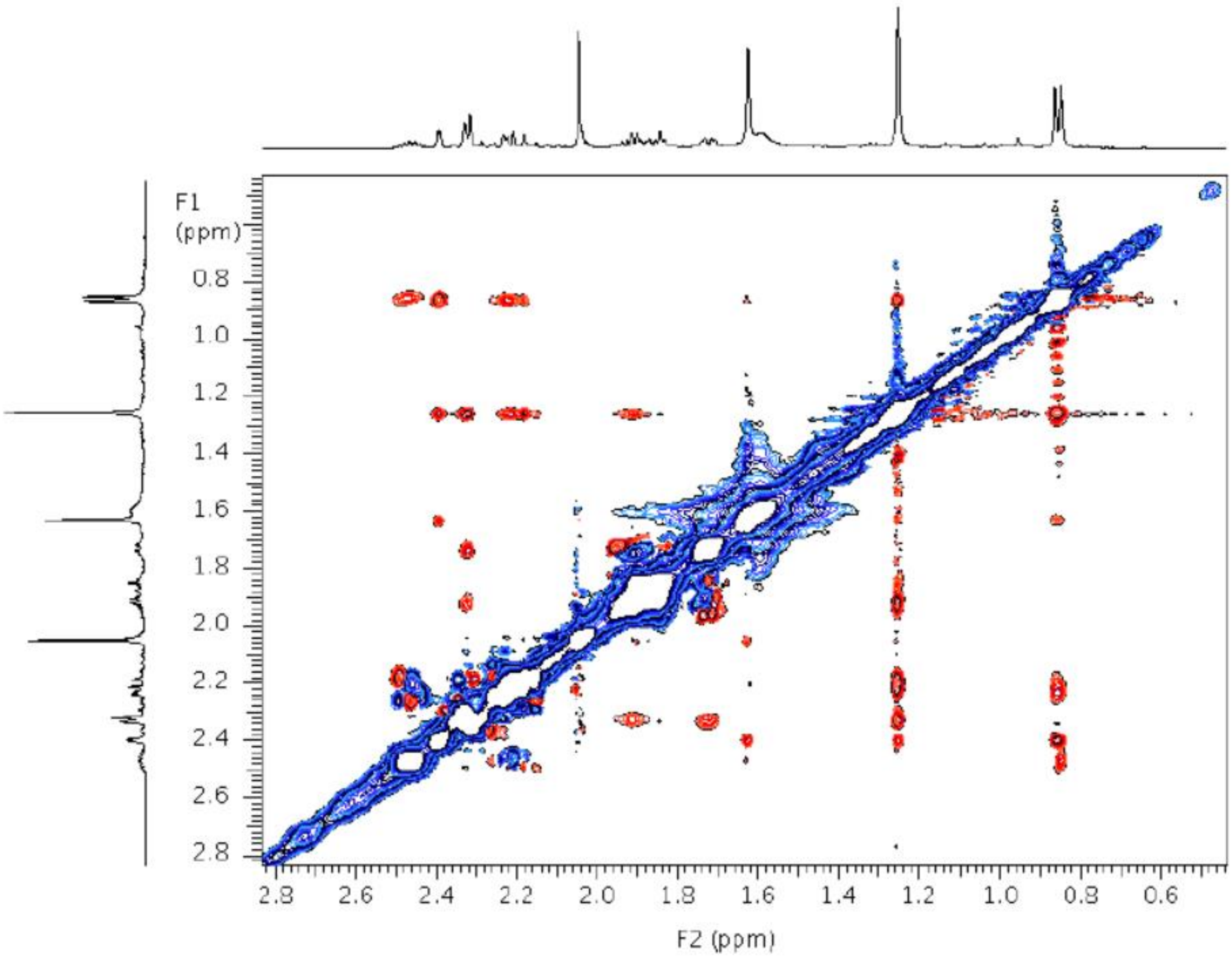

Figure S31. NOESY spectrum of calvukoellian I (**3**) in $CDCl_3$ (500 MHz).

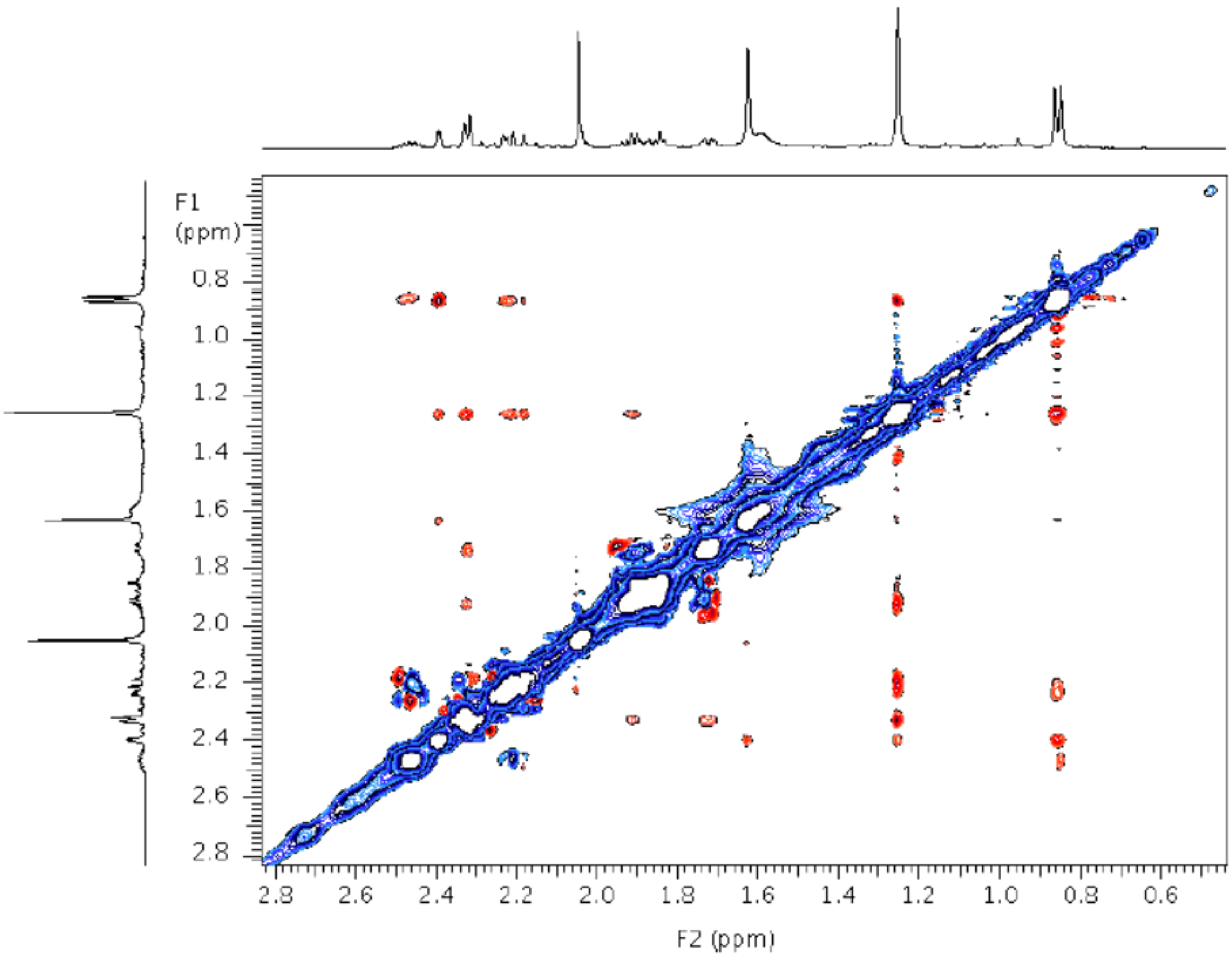

Figure S32. NOESY spectrum of calvukoellian I (**3**) in $CDCl_3$ (500 MHz).

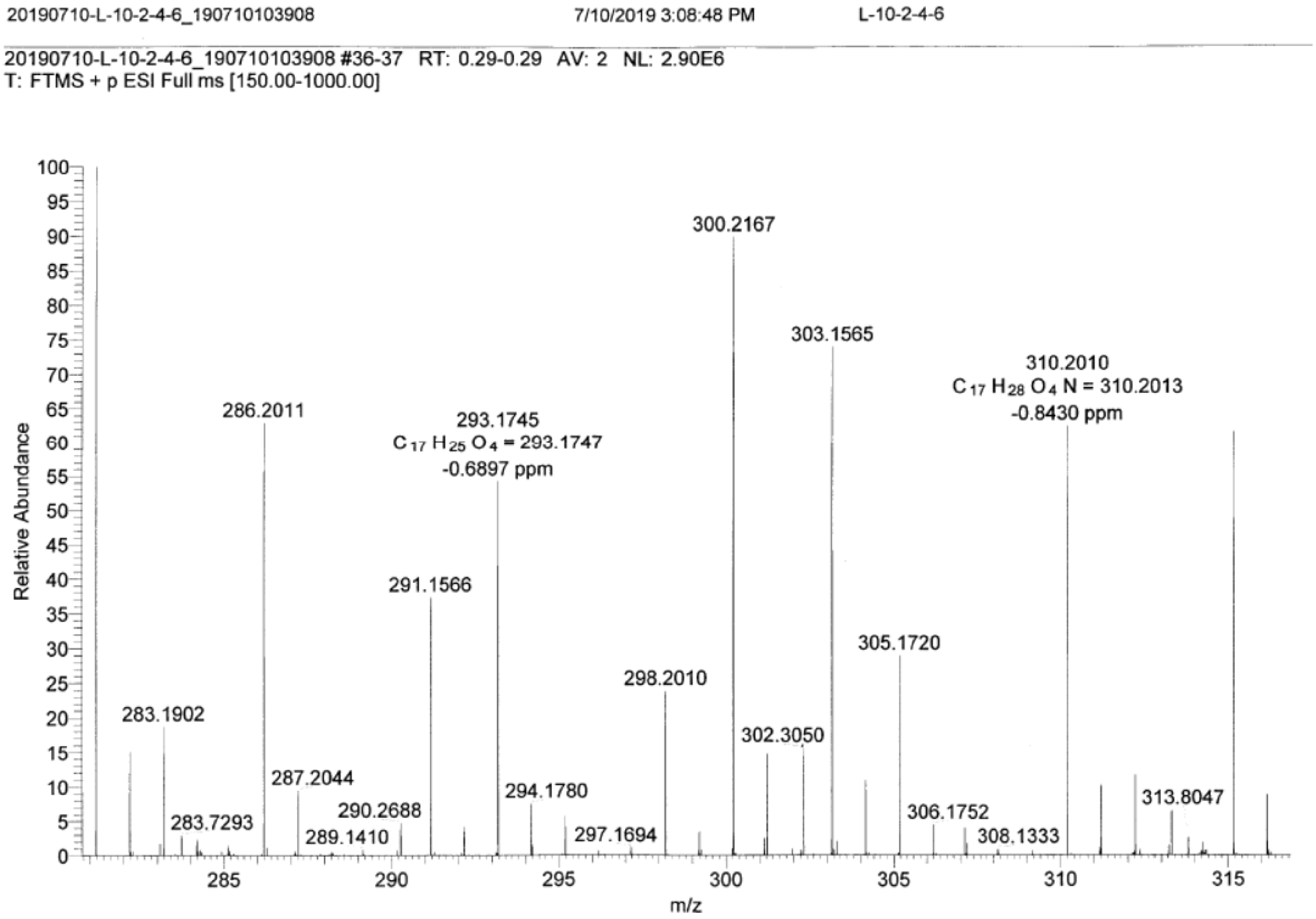

Figure S33. HRESIMS data of calvukoellian I (**3**).

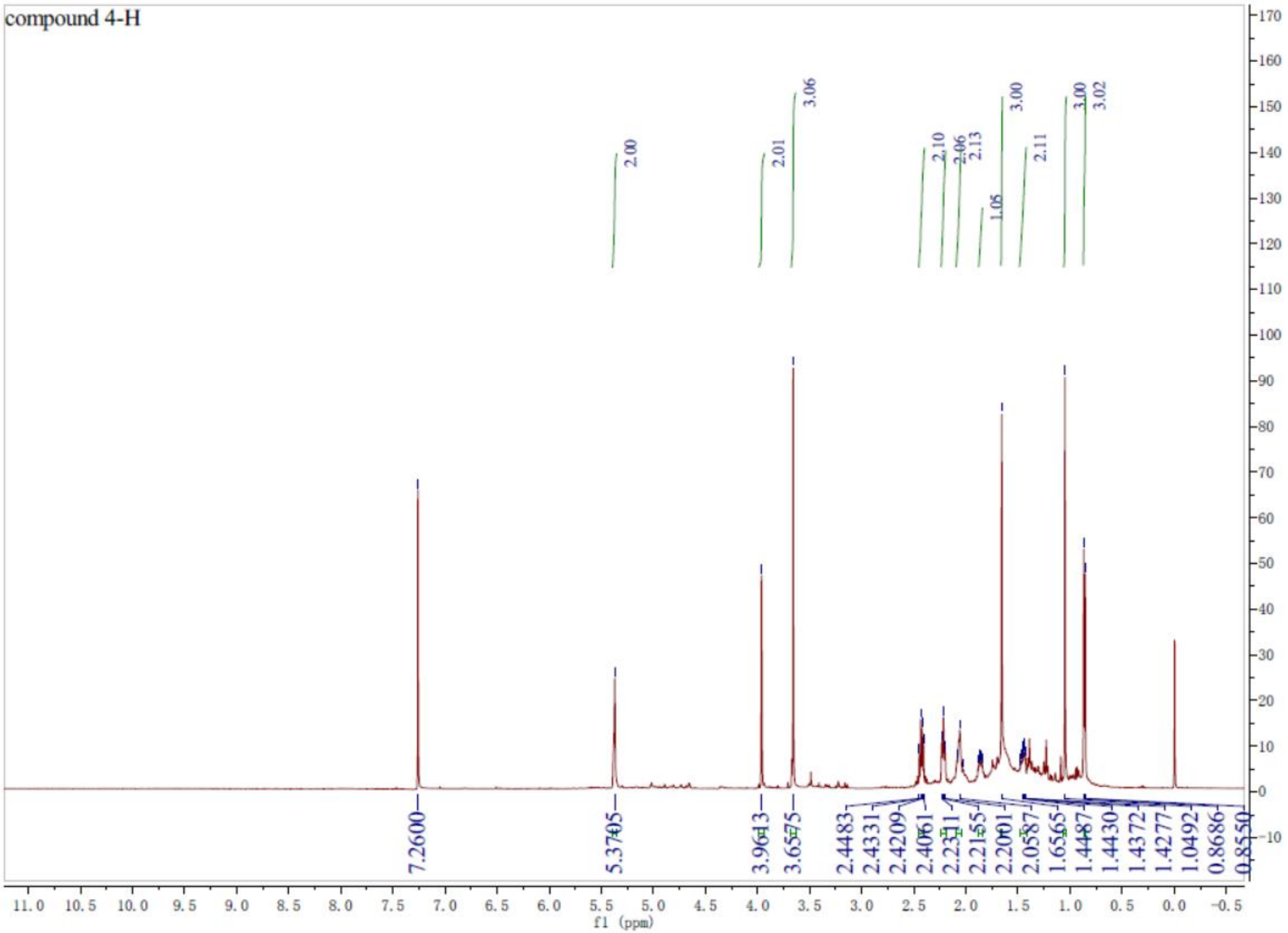

Figure S34. ^{1}H NMR spectrum of calvukoellian J (**4**) in $CDCl_3$ (500 MHz).

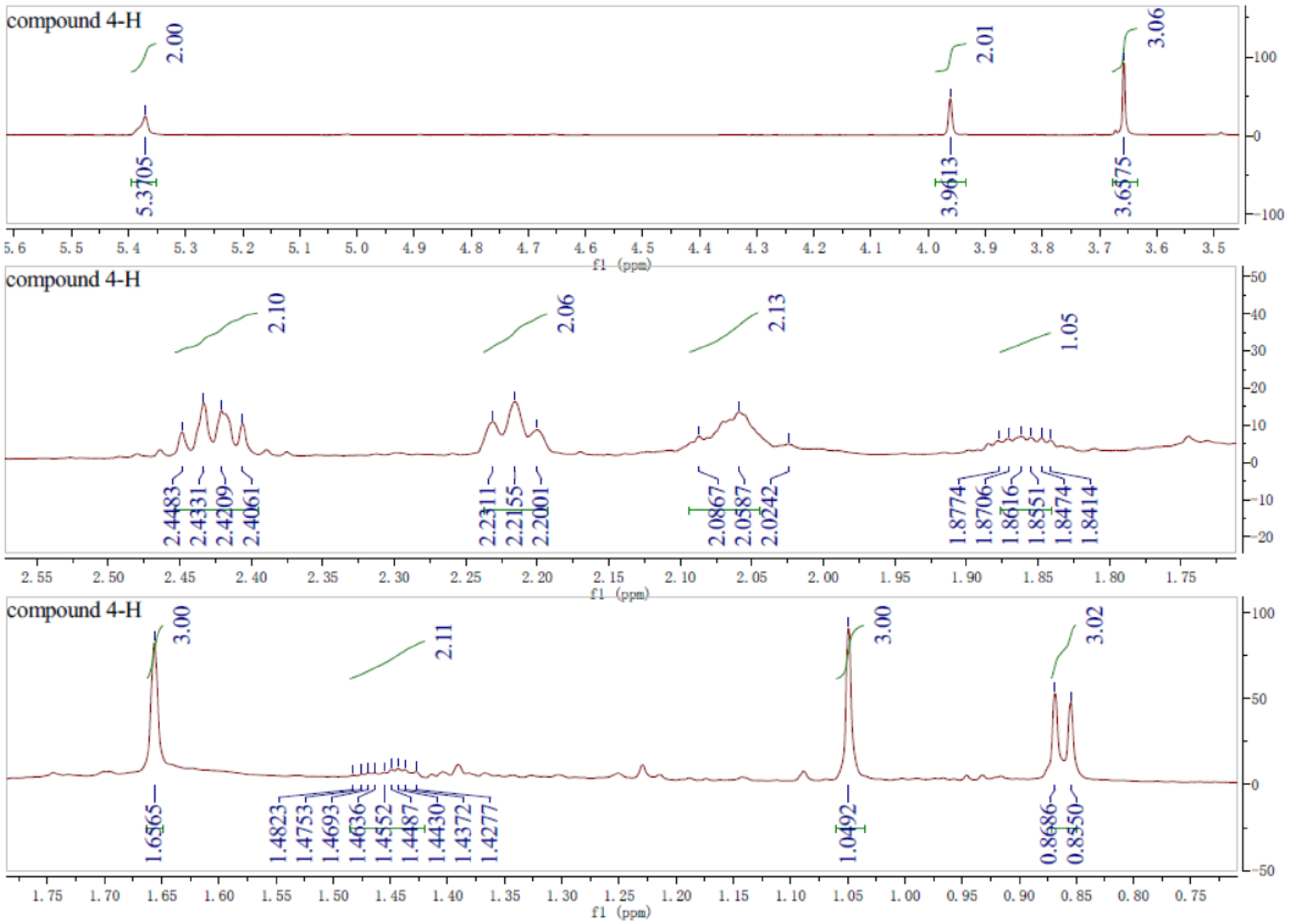

Figure S35. Enlarged ^{1}H NMR spectrum of calvukoellian J (**4**) in $CDCl_3$ (500 MHz).

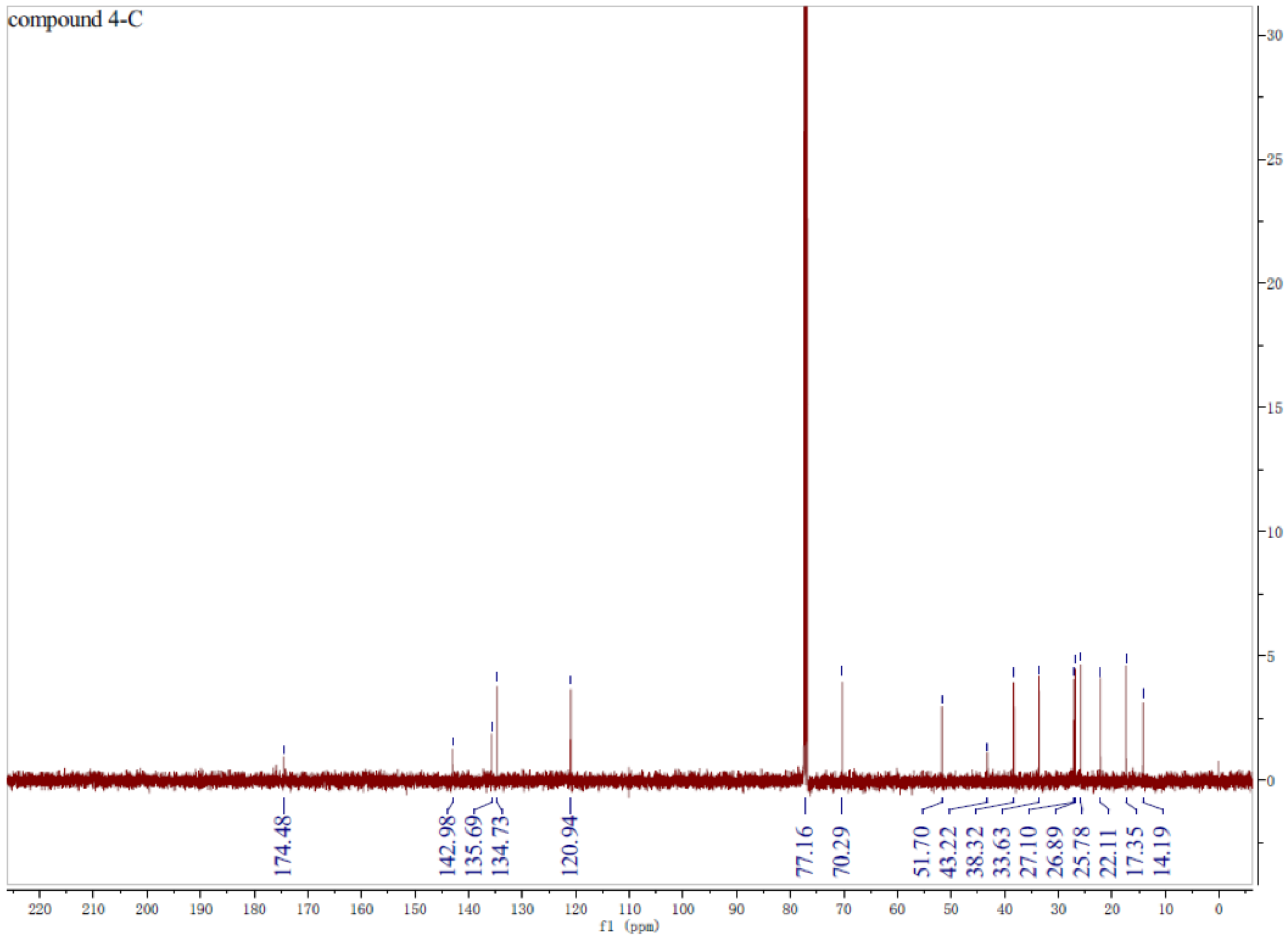

Figure S36. ^{13}C NMR spectrum of calvukoellian J (**4**) in $CDCl_3$ (500 MHz).

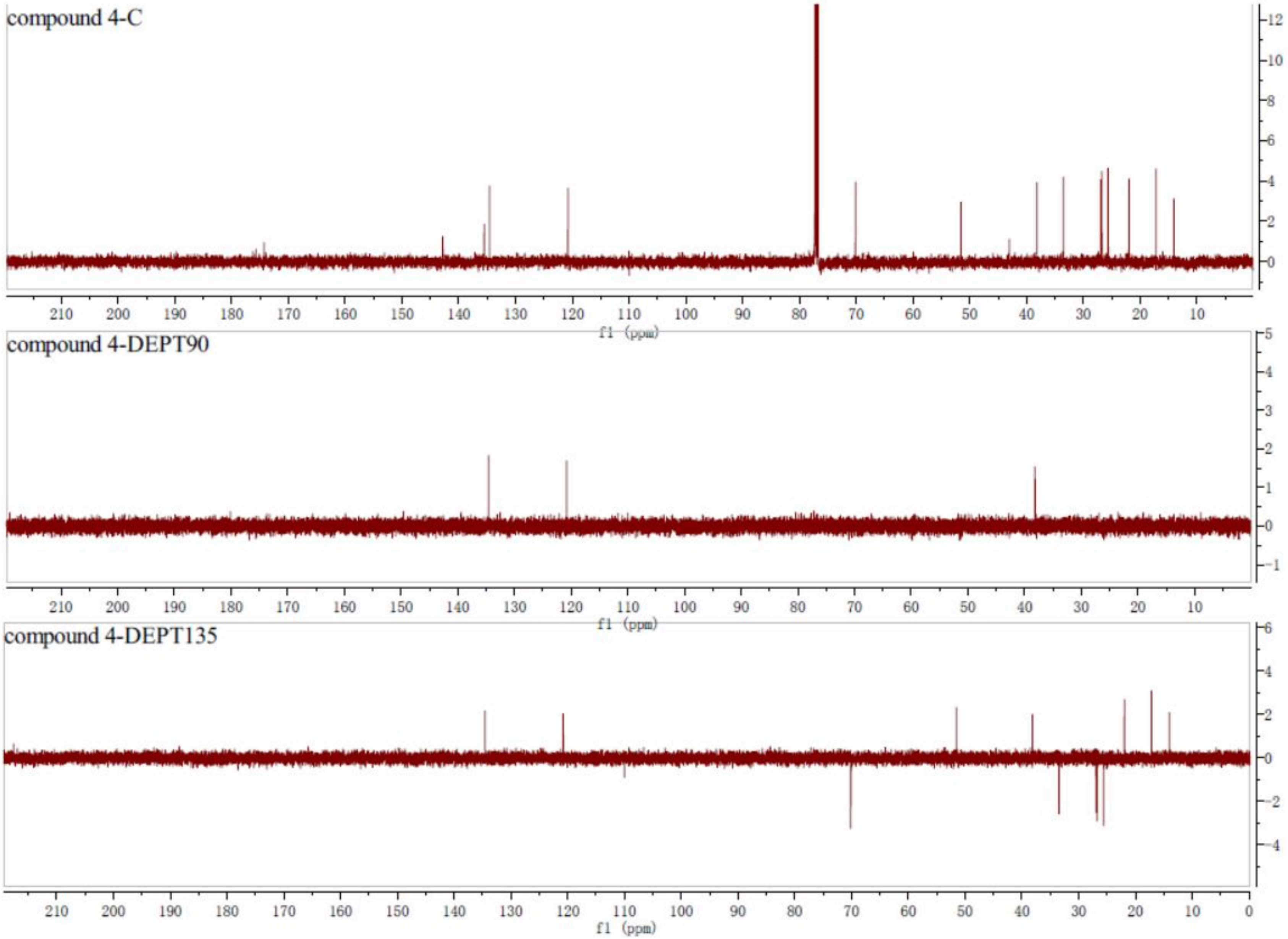

Figure S37. ^{13}C NMR and DEPT spectrum of calvukoellian J (**4**) in $CDCl_3$ (500 MHz).

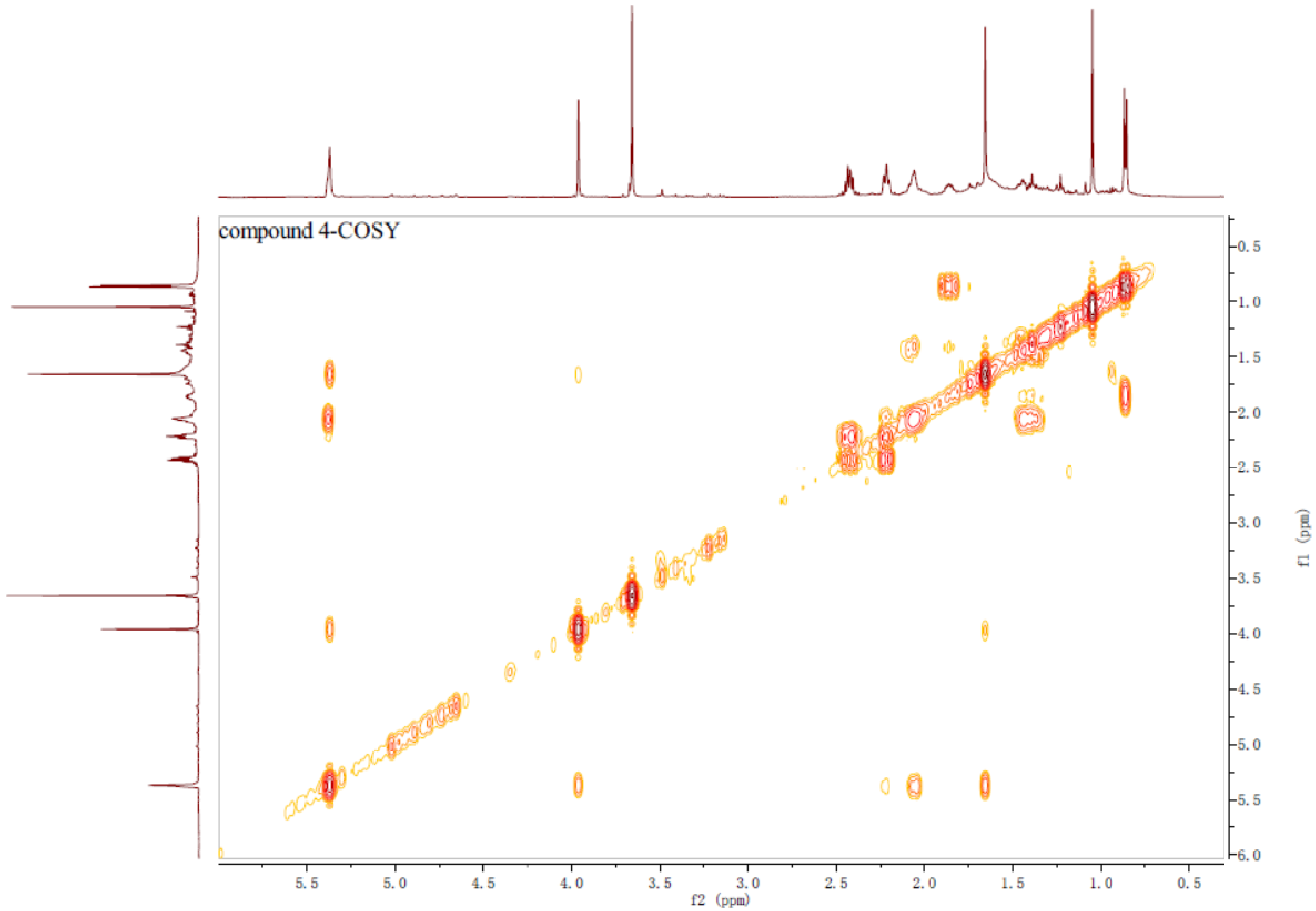

Figure S38. ¹H-¹H COSY spectrum of calvukoellian J (**4**) in $CDCl_3$ (500 MHz).

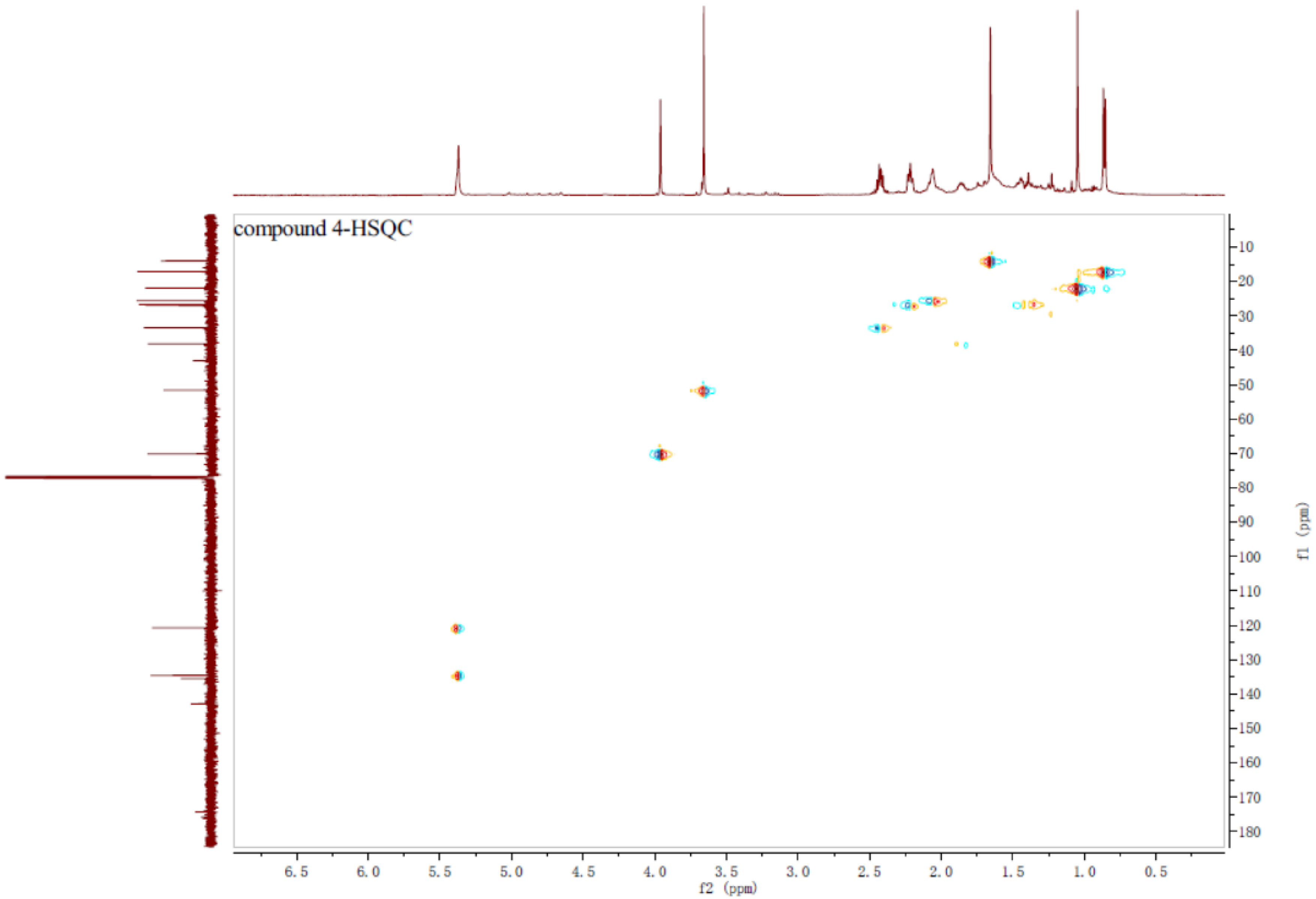
compound 4-HSQC
f2 (ppm)
f1 (ppm)

Figure S39. HSQC spectrum of calvukoellian J (**4**) in $CDCl_3$ (500 MHz).

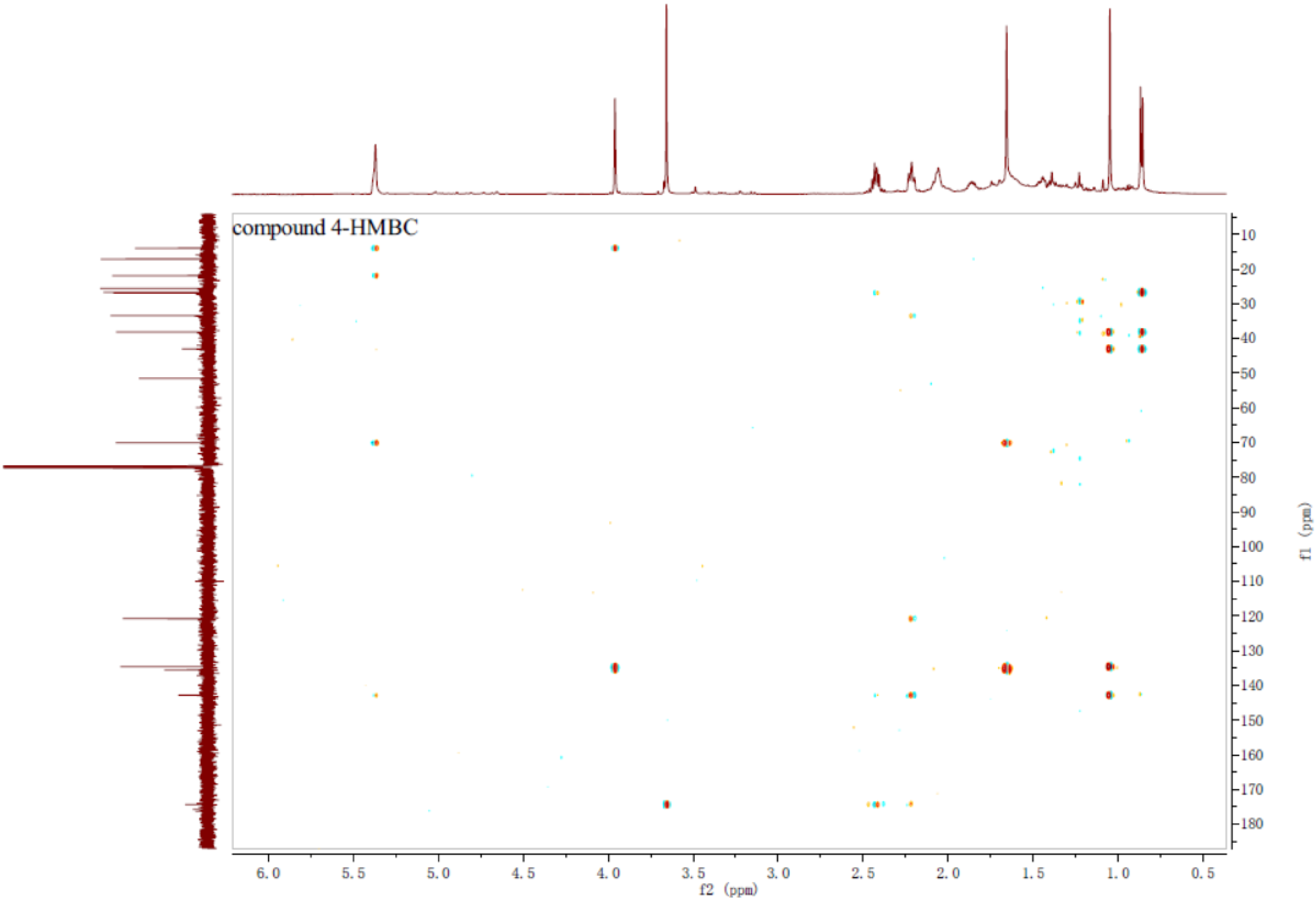

Figure S40. HMBC spectrum of calvukoellian J (**4**) in $CDCl_3$ (500 MHz).

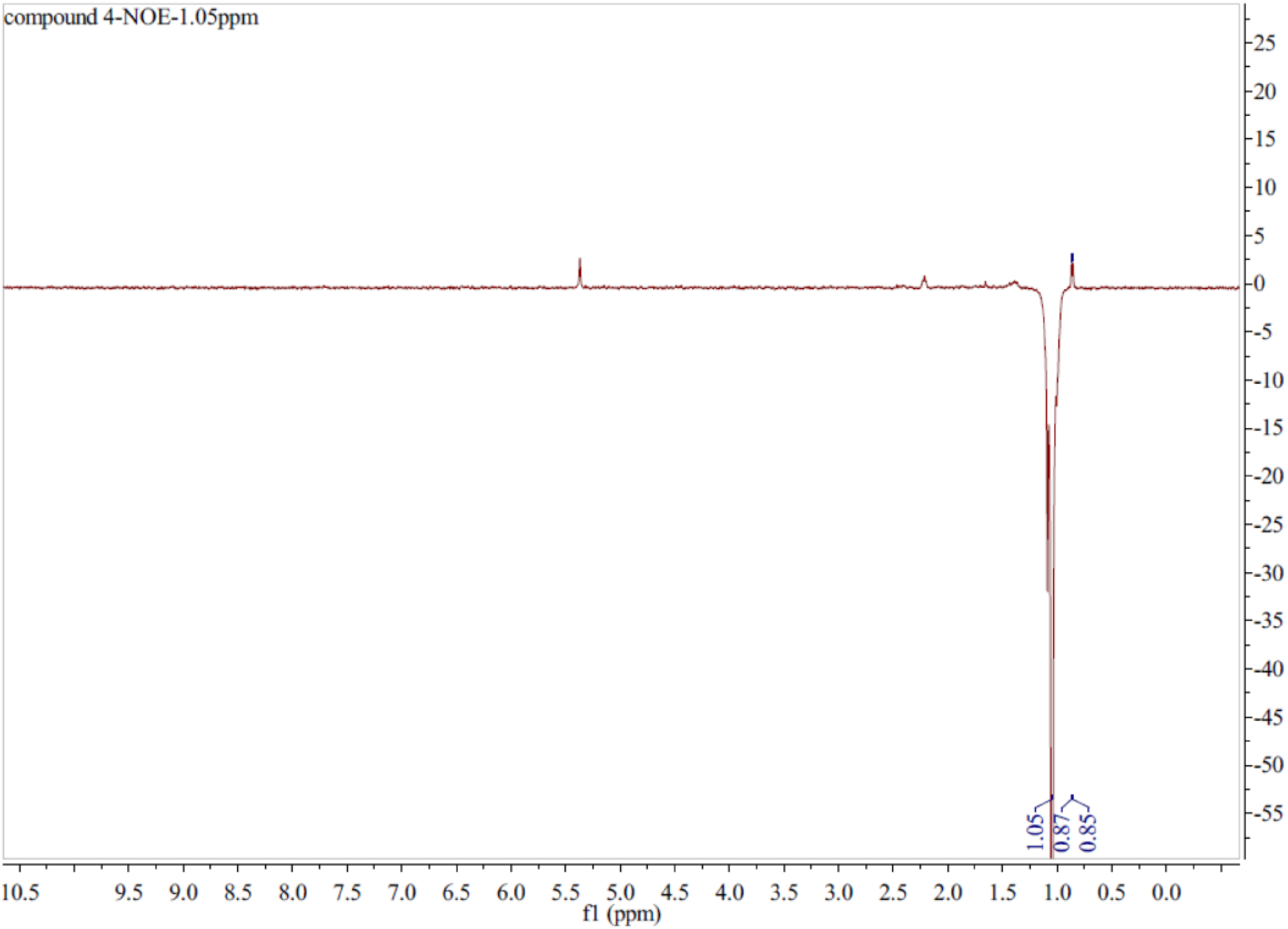

Figure S41. 1D-NOE spectrum of calvukoellian J (**4**) in $CDCl_3$ (500 MHz).

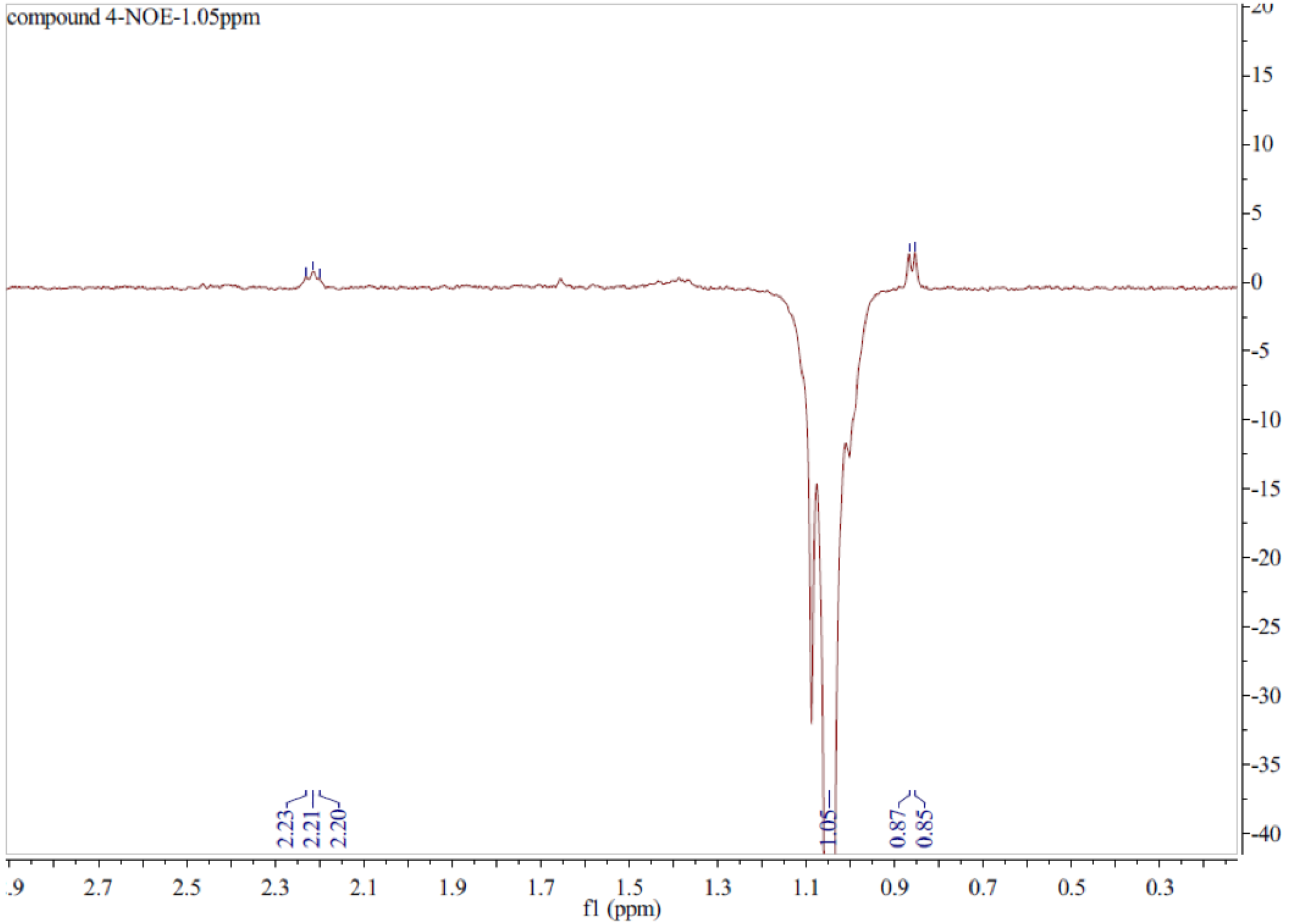

Figure S42. 1D-NOE spectrum of calvukoellian J (**4**) in $CDCl_3$ (500 MHz).

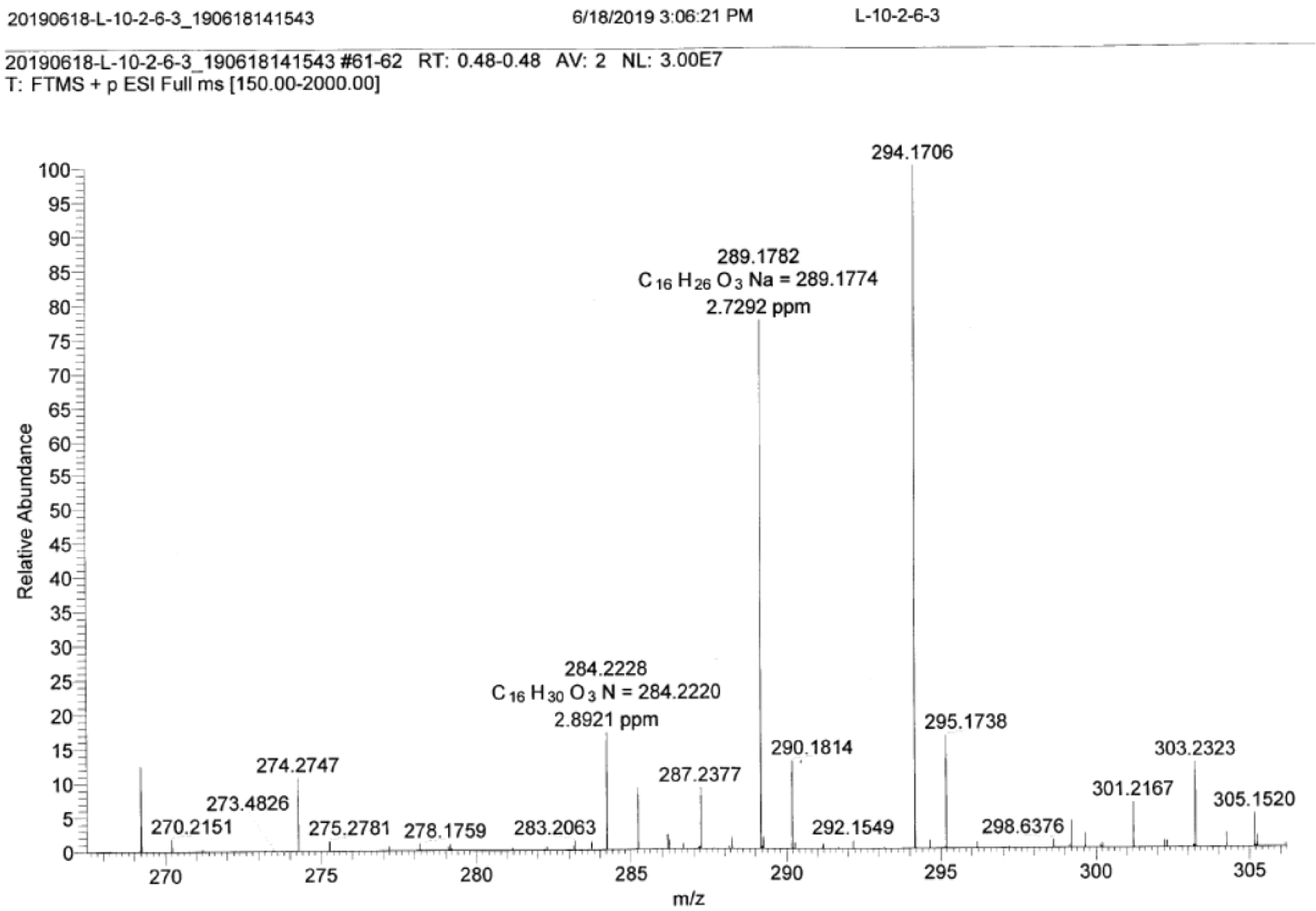

Figure S43. HRESIMS data of calvukoellian J (**4**).

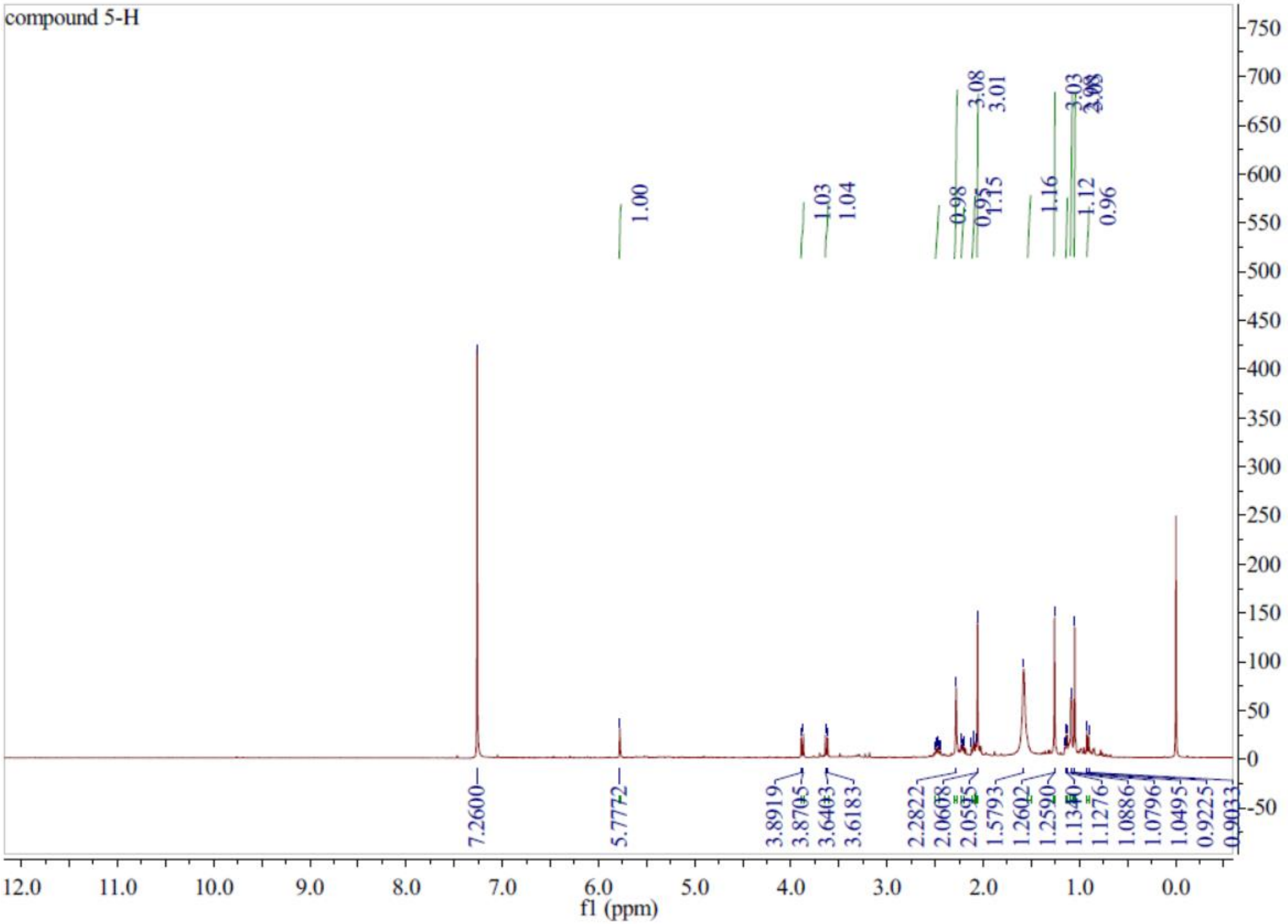

Figure S44. ^{1}H NMR spectrum of calvukoellian K (**5**) in $CDCl_3$ (500 MHz).

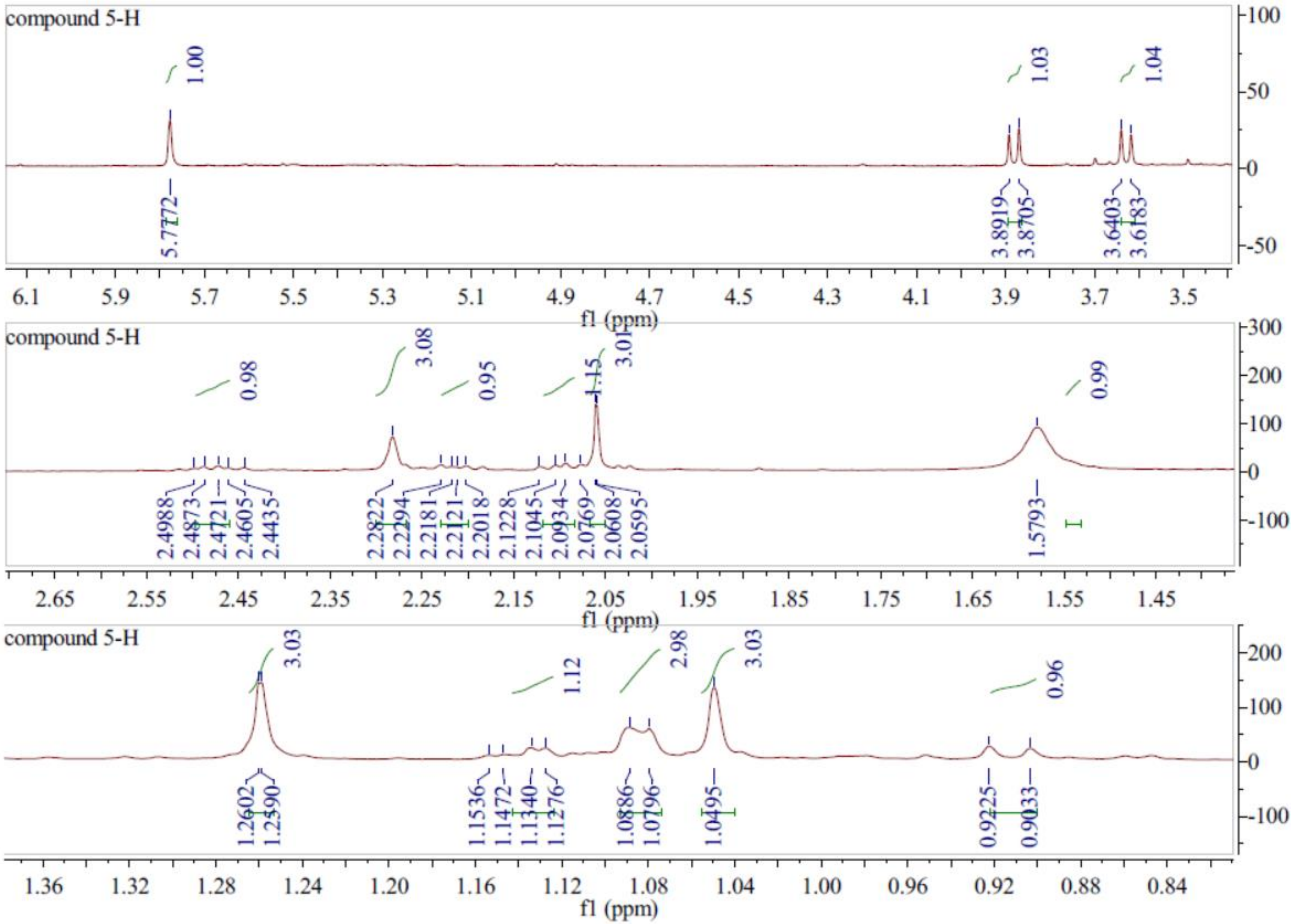

Figure S45. Enlarged ^{1}H NMR spectrum of calvukoellian K (**5**) in $CDCl_3$ (500 MHz).

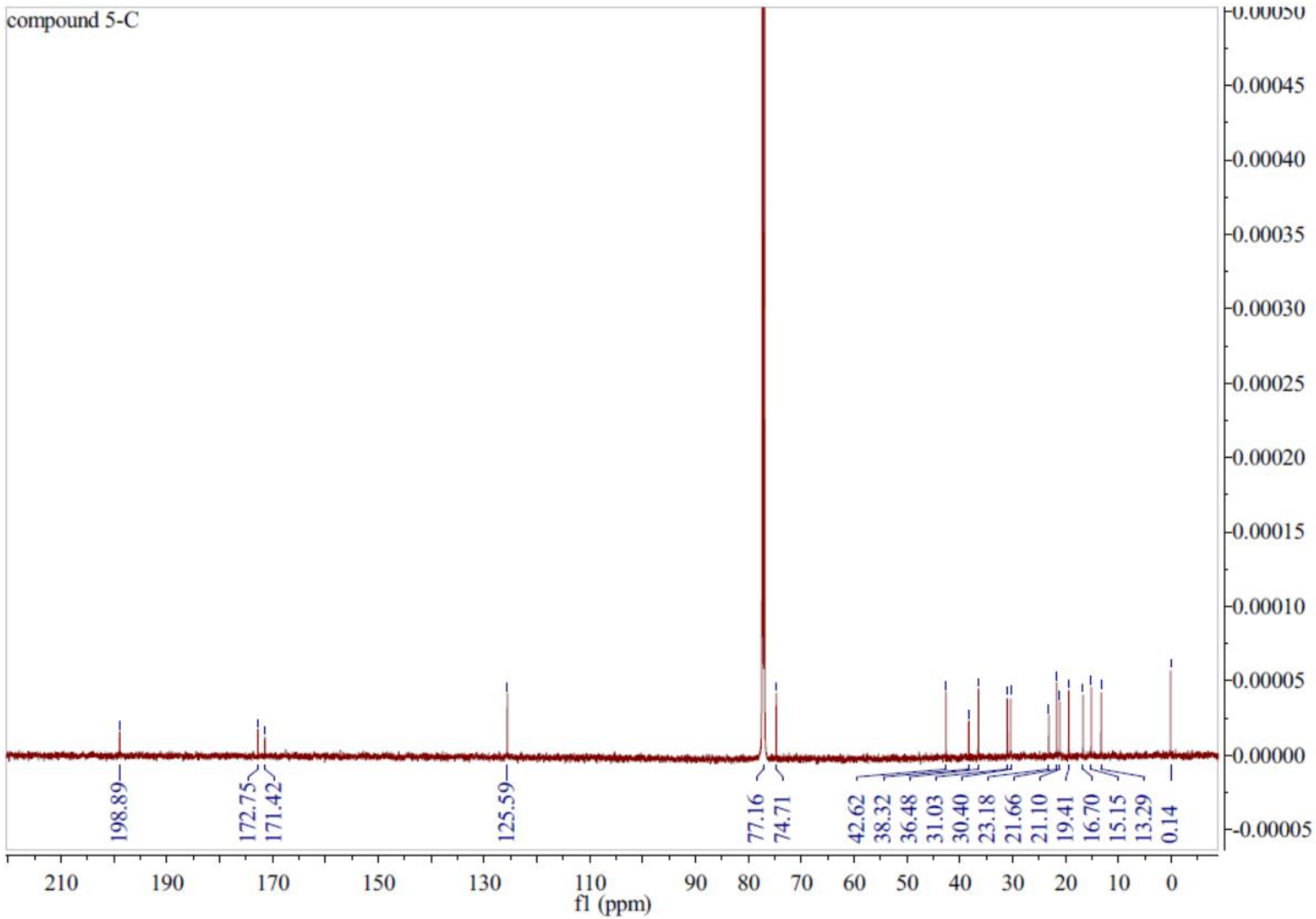

Figure S46. ^{13}C NMR spectrum of calvukoellian K (**5**) in $CDCl_3$ (125 MHz)

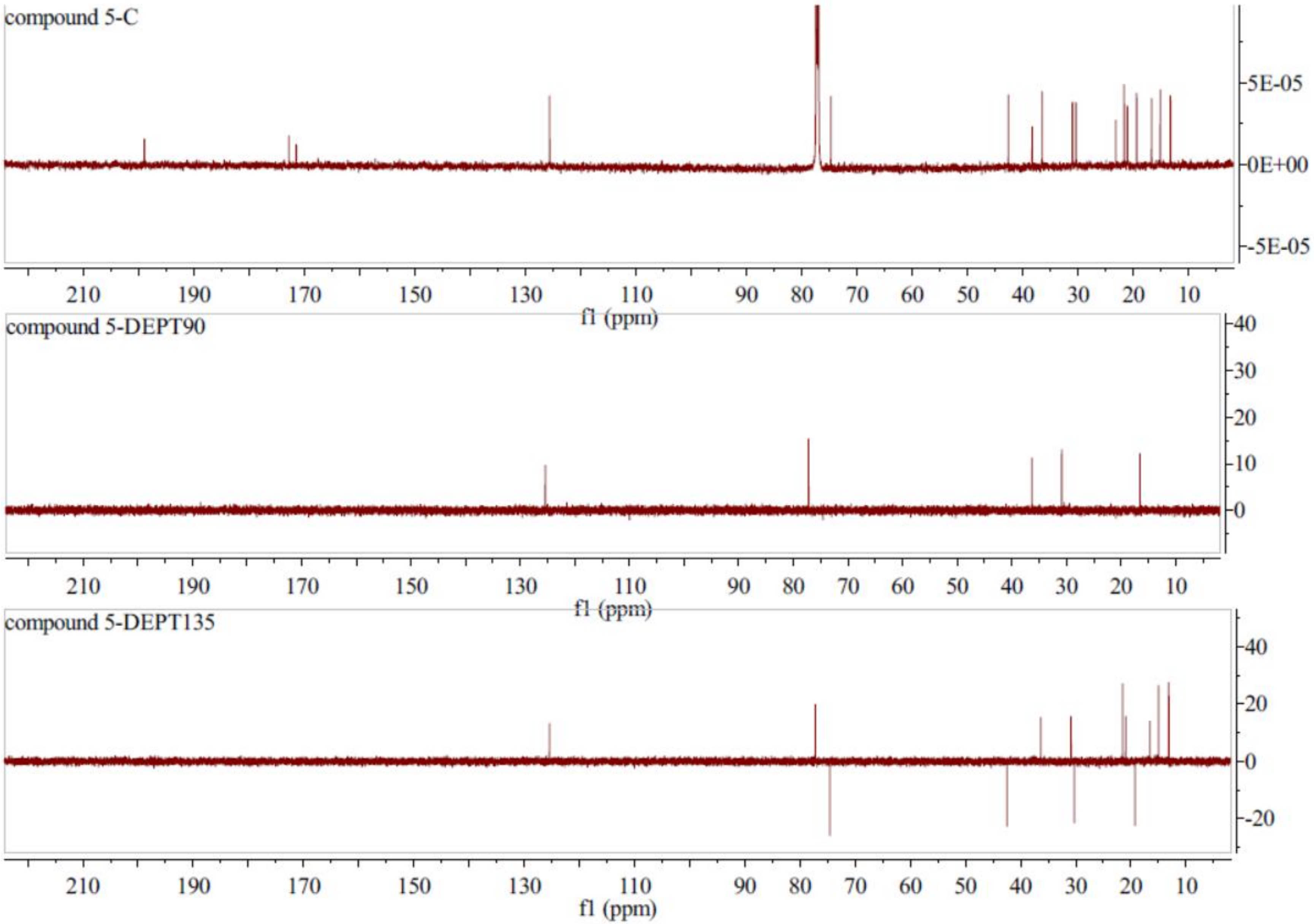

Figure S47. ^{13}C NMR and DEPT spectrum of calvukoellian K (**5**) in $CDCl_3$ (125 MHz).

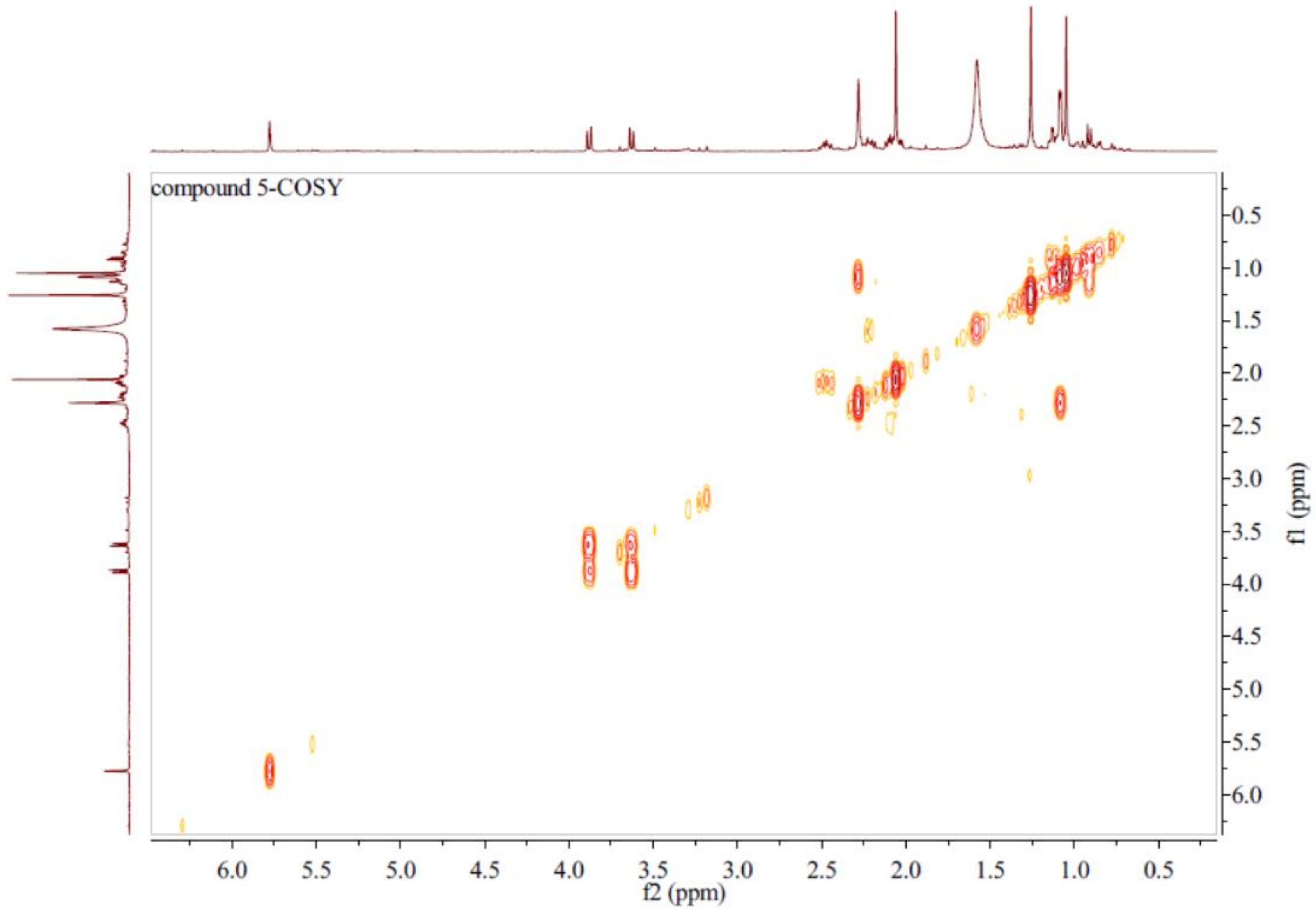

Figure S48. ^{1}H-^{1}H COSY spectrum of calvukoellian K (**5**) in $CDCl_3$ (500 MHz).

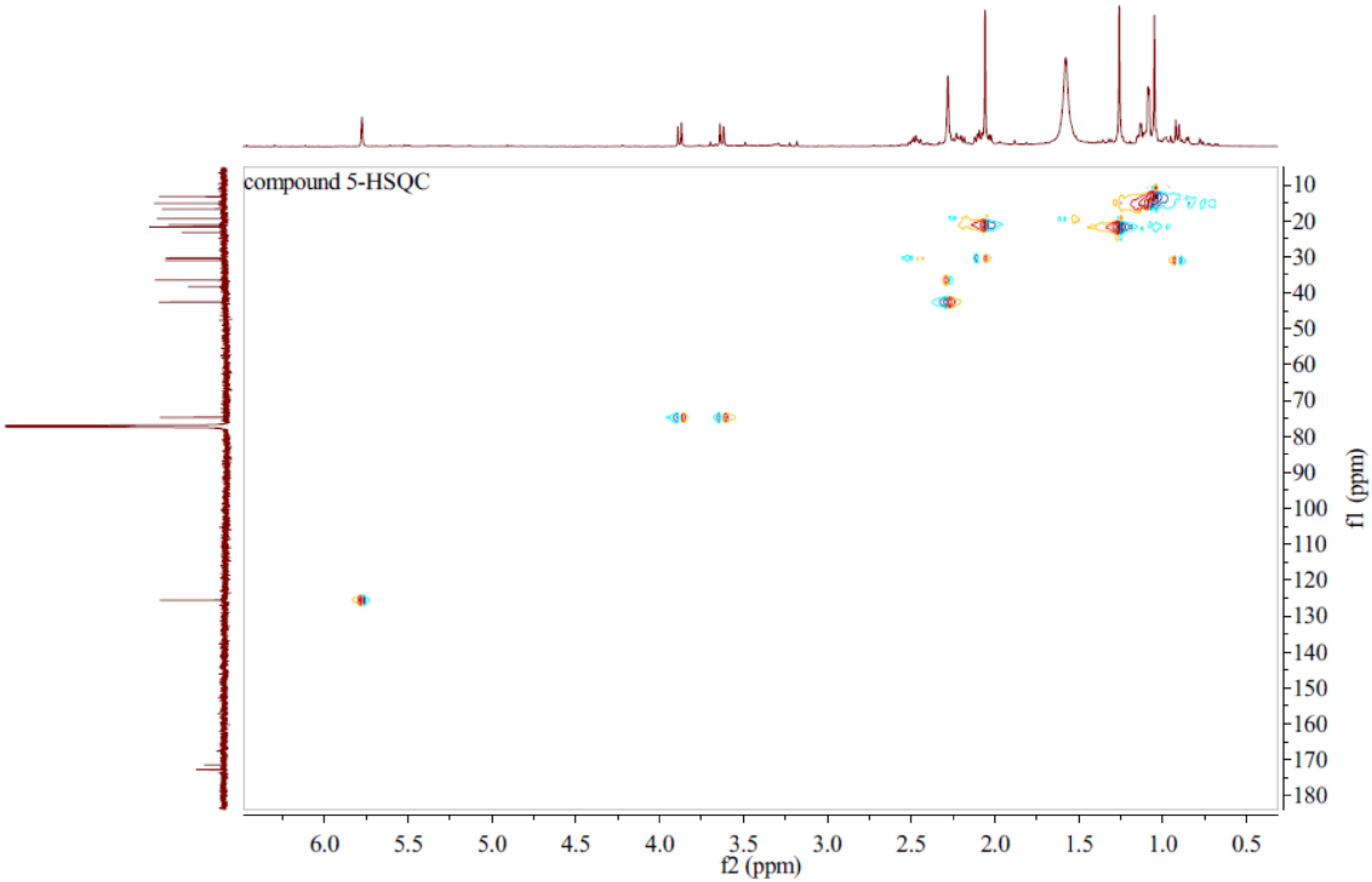

Figure S49. HSQC spectrum of calvukoellian K (**5**) in $CDCl_3$ (500 MHz).

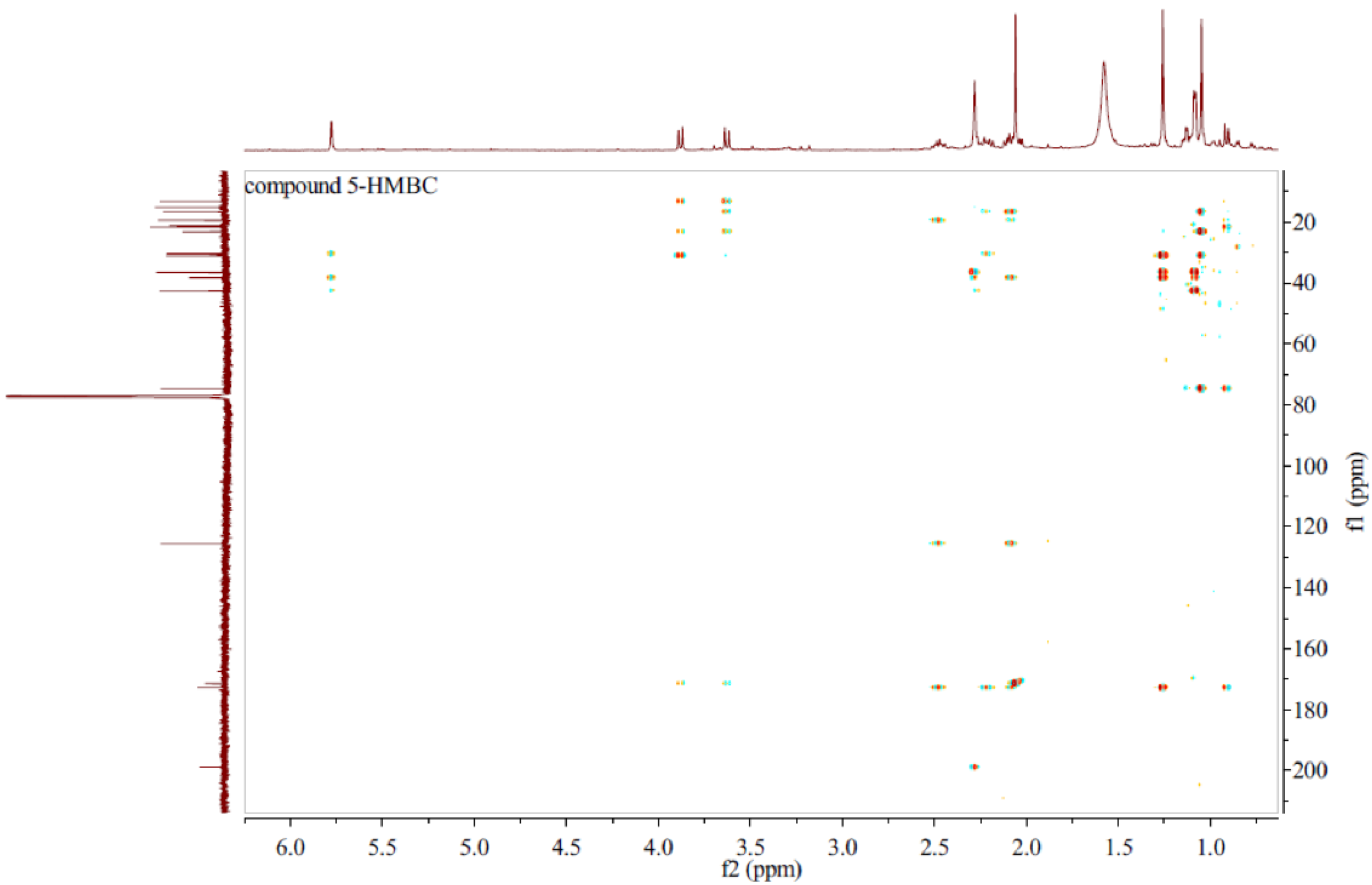

Figure S50. HMBC spectrum of calvukoellian K (**5**) in $CDCl_3$ (500 MHz).

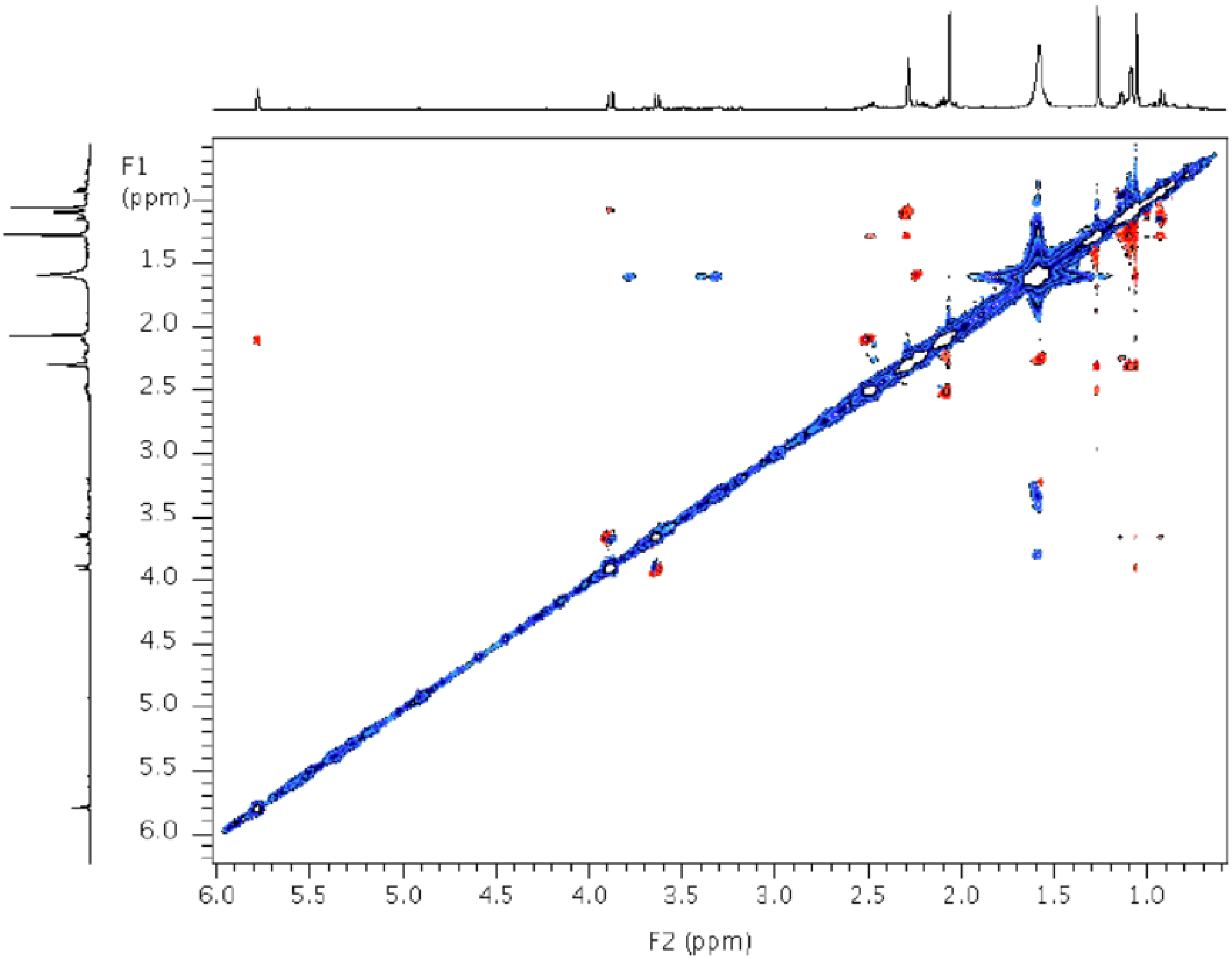

Figure S51. NOESY spectrum of calvukoellian K (**5**) in $CDCl_3$ (500 MHz).

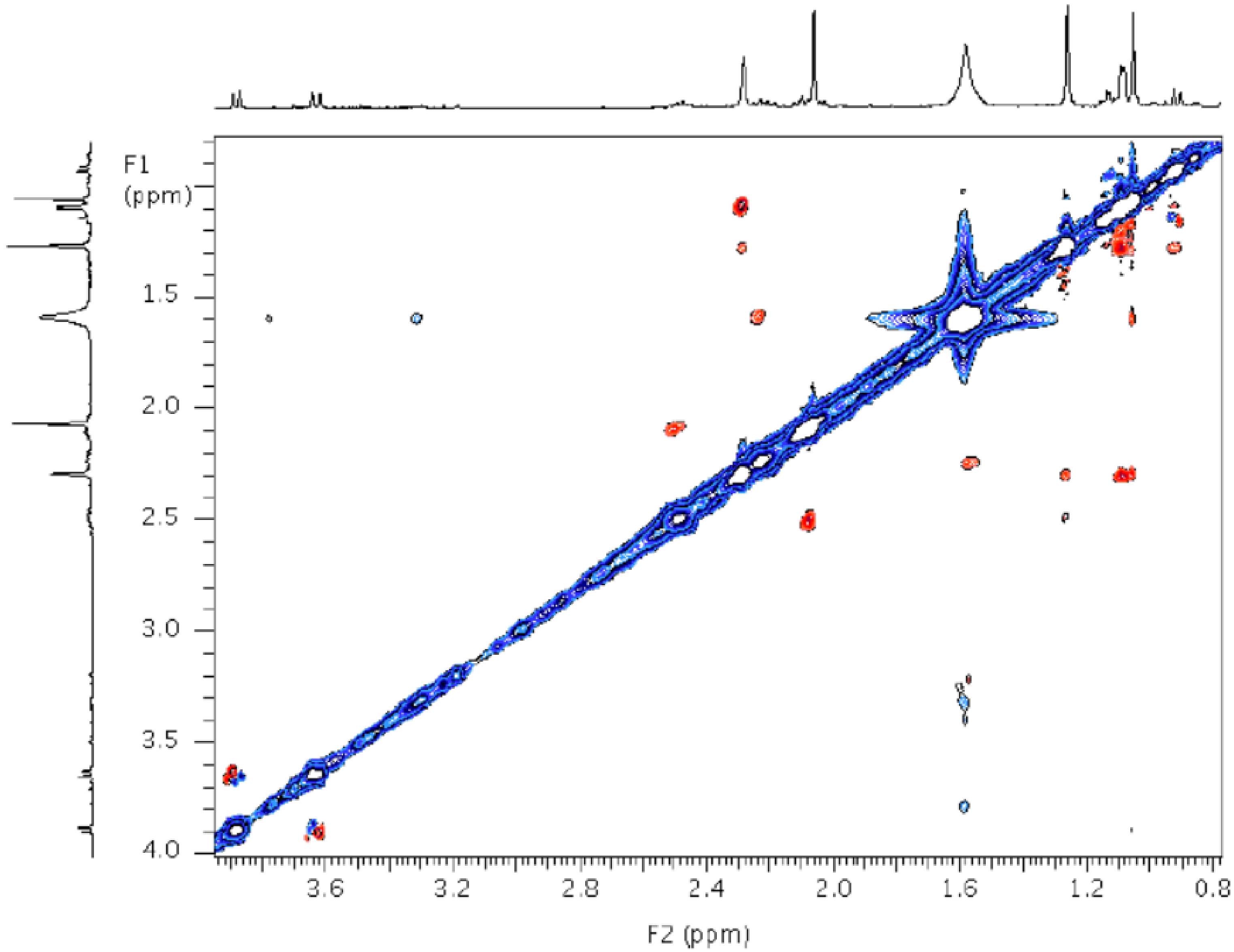

Figure S52. NOESY spectrum of calvukoellian K (**5**) in $CDCl_3$ (500 MHz).

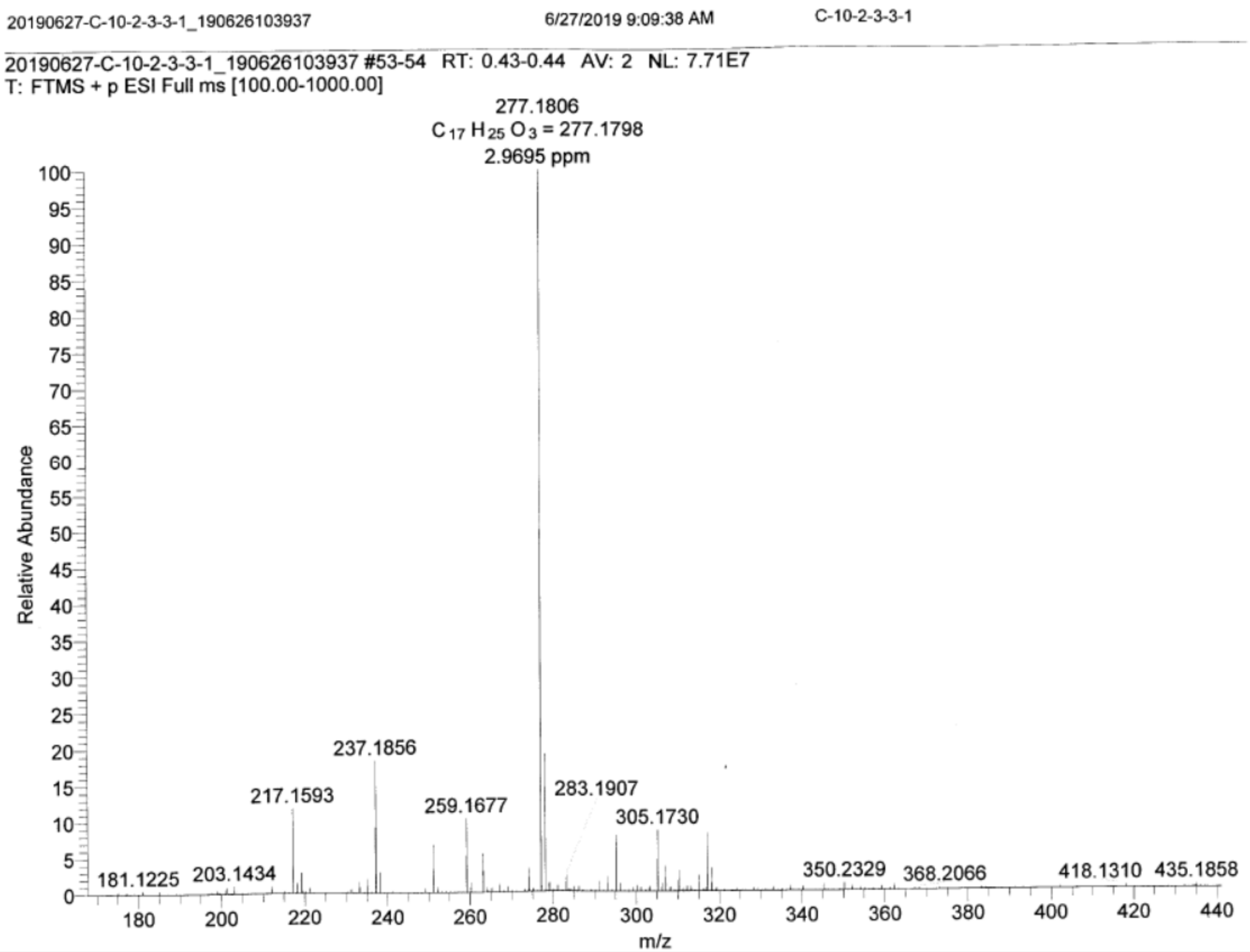

Figure S53. HRESIMS data of calvukoellian K (**5**).
